# Prognostic and Clinicopathological Significance of the Aberrant Expression of β-Catenin in Oral Squamous Cell Carcinoma: A Systematic Review and Meta-Analysis

**DOI:** 10.3390/cancers14030479

**Published:** 2022-01-18

**Authors:** Pablo Ramos-García, Miguel Á. González-Moles

**Affiliations:** 1 School of Dentistry, University of Granada, 18011 Granada, Spain; 2 Instituto de Investigación Biosanitaria ibs.GRANADA, 18012 Granada, Spain

**Keywords:** β-catenin, beta-catenin, CTNNB, Wnt signalling pathway, epithelial-mesenchymal transition, oral squamous cell carcinoma, prognosis, biomarker, systematic review, meta-analysis

## Abstract

**Simple Summary:**

β-catenin is a multifunctional protein whose physiological functions are mainly related to the maintenance of cell-cell adhesion by forming complexes with the adhesion molecule E-cadherin, both responsible for the preservation of squamous epithelia homeostasis. The loss of β-catenin expression in the cell membrane, the failure of cytoplasmic degradation mechanisms—essentially related to the activation of Wnt canonical oncogenic pathway—and/or its translocation to the nucleus—developing actions as a transcription factor of oncogenes—are aberrant mechanisms with oncogenic implications in oral carcinogenesis. In this systematic review and meta-analysis on 41 studies and 2746 oral squamous cell carcinoma (OSCC) patients we demonstrate that the aberrant expression of β-catenin—mainly the immunohistochemical analysis of its loss in the cell membrane—behaves as a prognostic biomarker, significantly associated with poor survival, essentially linked to the increased risk for the development of lymph node metastases, higher tumour size and clinical stage in these patients.

**Abstract:**

This systematic review and meta-analysis aims to evaluate the prognostic and clinicopathological significance of the aberrant expression of β-catenin (assessed through the immunohistochemical loss of membrane expression, cytoplasmic and nuclear expression) in oral squamous cell carcinoma (OSCC). We searched for primary-level studies published before October-2021 through PubMed, Embase, Web of Science, Scopus, and Google Scholar, with no limitation in regard to their publication date or language. We evaluated the methodological quality and risk of bias of the studies included using the QUIPS tool, carried out meta-analyses, explored heterogeneity and their sources across subgroups and meta-regression, and conducted sensitivity and small-study effects analyses. Forty-one studies (2746 patients) met inclusion criteria. The aberrant immunohistochemical expression of β-catenin was statistically associated with poor overall survival (HR = 1.77, 95% CI = 1.20–2.60, *p* = 0.004), disease-free survival (HR = 2.44, 95% CI = 1.10–5.50, *p* = 0.03), N+ status (OR = 2.39, 95% CI = 1.68–3.40, *p* < 0.001), higher clinical stage (OR = 2.40, 95% CI = 1.58–3.63, *p* < 0.001), higher tumour size (OR = 1.76, 95% CI = 1.23–2.53, *p* = 0.004), and moderately-poorly differentiated OSCC (OR = 1.57, 95% CI = 1.09–2.25, *p* = 0.02). The loss of β-catenin in the cell membrane showed the largest effect size in most of meta-analyses (singularly for poor overall survival [HR = 2.37, 95% CI = 1.55–3.62, *p* < 0.001], N+ status [OR = 3.44, 95% CI = 2.40–4.93, *p* < 0.001] and higher clinical stage [OR = 2.51, 95% CI = 1.17–5.35, *p* = 0.02]). In conclusion, our findings indicate that immunohistochemical assessment of the aberrant expression of β-catenin could be incorporated as an additional and complementary routine prognostic biomarker for the assessment of patients with OSCC.

## 1. Introduction

Oral cancer is a growing worldwide public health problem, presenting an incidence of 377,713 new cases and 177,757 deaths per year (GLOBOCAN, IARC, WHO) [1]. Oral squamous cell carcinoma (OSCC) accounts for approximately 90% of oral malignancies and has a 5-year mortality rate of close to 50% [1,2]. Prediction of the prognosis is of major importance in this tumour, currently based on clinicopathological parameters (i.e., Tumour Node Metastasis [TNM] staging system), the most influential prognostic factors being the development of lymph node metastases and the presence of extracapsular extension [2]. OSCC is a complex and heterogeneous disease in molecular terms [1,2], being accepted in recent years that at least two genetic subclasses should be distinguished, determined by their association with human papillomavirus (HPV) infection: HPV-positive tumours and HPV-negative tumours, with differential risk profile patterns [2]. In both subgroups, a male predilection is currently accepted. Nevertheless, HPV-negative tumours are more frequently associated with heavy tobacco use and alcohol consumption. On the other hand, HPV-positive tumours preferentially develop within the oropharynx and are more likely to occur in younger patients, higher socioeconomic status, and an increased number of lifetime sexual partners with oral sex behaviours [2]. Recent evidence also support differences in molecular genetic profiles related to HPV infection status. HPV-positive tumours harbour an active transcription of the major viral oncoproteins E6 and E7 and frequent losses of chromosomes 9p, 3p, and 17p. The tumour suppressor genes *TP53*—which encodes p53—and *CDKN2A*—which encodes p16—are located at 17p13 and 9p21, respectively. Consequently, p53 and p16 mutations are frequent in HPV-negative OSCC. Molecular alterations seem different in HPV-positive OSCC, usually lacking such chromosomal losses, presenting the decreased expression of wild-type p53 (due to the inactivation and degradation by E6), and exhibiting increased p16 expression (due to the inactivation of retinoblastoma protein [pRb] by E7, with cell cycle arrest and p16 accumulation) [2]. The prognostic value of molecular biomarkers is attracting considerable research interest and, in this sense, recent advances are suggesting a potential oncogenic and prognostic role for β-catenin in OSCC [3].

β-catenin is a multifunctional protein that belongs to the Armadillo family, localized in its physiological form in the cell membrane [3]. The main function of this protein is related to the maintenance of cell-cell adhesion, for which the formation of complexes between β-catenin and E-cadherin, another key membrane molecule in the maintenance of cell adhesion, is essential. β-catenin/E-cadherin complexes are responsible for the preservation of the structure and normal function of squamous epithelia [3]. The research focused on β-catenin has shown in premalignant and malignant squamous epithelia also its cytoplasmic and nuclear expression [3]. Cytoplasmic β-catenin is related to the failure in the mechanisms involved in its degradation, which is physiologically carried out by a multiprotein complex composed of axin, Adenomatous Polyposis Coli protein (APC), casein protein kinase 1 (CK1), and glycogen synthetase 3 (GSK3) [4]. Cytoplasmic degradation of β-catenin is highly dependent on the actions of axin, which is tasked with coordinating the sequential phosphorylation of β-catenin, first at serine 45 -to enable β-catenin to interact with CK1-, and subsequently at threonine 41 and serine 37 and 33 -to enable β-catenin to interact with GSK3-. These phosphorylation events create a binding site on β-catenin for β-trcp E3 ubiquitin ligase that in turn catalyses the proteosomal degradation of β-catenin by ubiquitinylation, removing it from the cytoplasm [5]. The failure of physiological degradation of β-catenin enables its cytoplasmic accumulation, which is essentially related to the activation of the Wnt canonical oncogenic pathway. In this context, Wnt proteins, upon binding to their membrane receptors (Fz, LRP5, and LRP6) form complexes (Wnt/Fz/LRP5/LRP6) that recruit the dishevelled protein, which induces phosphorylation of LRP5/6, resulting in axin sequestration from the protein complex responsible for the physiological degradation of β-catenin (axin/APC/CK1/GSK3). Therefore, activation of the Wnt canonical pathway results in the accumulation of β-catenin in the cytoplasm due to a failure of its degradation, which allows its translocation to the nucleus and the development of its actions as a transcription factor of oncogenes involved in processes associated with tumour development [3,6]. Moreover, regardless of the function as an oncogene transcription factor, β-catenin also exerts oncogenic actions related to the loss of its membrane expression which leads to cell adhesion failure, for which the concomitant loss of E-cadherin is necessary. The oncogenic mechanisms linked to the loss of membranous expression of E-Cadherin/β-catenin are primarily related to the development of the epithelial-mesenchymal transition phenomenon (EMT) which, among other consequences, induces an increase in the invasiveness of tumour cells [7,8]. Oncogenic actions linked to alterations in β-catenin function have been demonstrated in primary level studies in some premalignant epithelia and tumours, including Barret’s oesophagus [9], colon adenocarcinoma [10], laryngeal carcinoma [11], and also in oral premalignant epithelia and OSCC [3,12,13,14,15,16]. Our research group has recently focused on the oncogenic effect of β-catenin in the development of oral and lip cancer [3,17,18], concluding that the main oncogenic function of β-catenin is related to the loss of membranous expression and the consequently increased invasiveness of tumour cells.

However, despite the attention that β-catenin has received as an oncogenic protein, to date, there is no study designed to provide high scientific evidence on the implications of this protein in oral carcinogenesis. On this background, the aim of our study was to present and interpret the results of a systematic review and meta-analysis on the implications of aberrant expression of β-catenin (loss of membrane expression, cytoplasmic expression, and nuclear expression) on the development and prognosis of OSCC.

## 2. Materials and Methods

This systematic review and meta-analysis complied with PRISMA and MOOSE reporting guidelines, and closely followed the criteria of Cochrane Prognosis Methods Group [19] and Cochrane Handbook for Systematic Reviews of Interventions [20], and was conducted and validated in accordance with AMSTAR2 guidelines [21].

### 2.1. Protocol

In order to minimize the risk of bias, improve the transparency, precision, and integrity of our systematic review and meta-analysis, a protocol on its methodology has been a priori designed and submitted to PROSPERO International Prospective Register of Systematic Reviews (www.crd.york.ac.uk/PROSPERO, accessed on 27 December 2021) (ID300438 was assigned; a copy of the protocol was included in the Supplementary Materials). The protocol complied with the PRISMA-P statement in order to ensure a rigorous approach [22].

### 2.2. Search Strategy

We searched PubMed, Embase, Web of Science, and Scopus databases for studies published before October-2021 (upper limit), with no lower date limit. Searches were conducted by combining the thesaurus terms used by the databases (i.e., MeSH and EMTREE) with free terms (Table S1), designed and built to maximize sensitivity. An additional screening was performed by hand-searching the reference lists of retrieved included studies and using Google Scholar. All references were managed using Mendeley v.1.19.8 (Elsevier, Amsterdam, The Netherlands); the process of eliminating duplicate references was also driven with this software.

### 2.3. Eligibility Criteria

Inclusion criteria: (1) Original primary-level studies, without language, publication date, follow up periods, study design, geographical area, sex or age restrictions; (2) Evaluation of β-catenin protein expression in samples from OSCC; (3) Analysis of the association of β-catenin with at least one of the following prognostic and/or clinicopathological variables: overall survival (OS), disease-free survival (DFS), T status, N status, clinical stage, or histological grade. OS was defined as the time elapsed from date of diagnosis/surgery to date of death by any cause. DFS was defined as the time elapsed from diagnosis/surgery to the detection of locoregional or distant recurrence or to death without recurrence. Given the lack of international consensus standards to define survival endpoints, any study using the terms OS/DFS was included, or by using other terms in compliance with our preceding definitions.

Exclusion criteria were: (1) Retracted articles, reviews, meta-analyses, case reports, editorials, letters, meeting abstracts, personal opinions, comments, or book chapters; (2) In vitro research or in vivo animal experimentation; (3) Squamous cell carcinomas of anatomic areas distinct to the oral cavity, and/or tumours of different histopathological lineage; (4) Evaluation of β-catenin/*CTNNB* genomic alterations (e.g., mutations, gene amplification or deletion, polymorphisms, etc.); (5) No analysis of the main prognostic or clinicopathological variables of interest; (6) Lack or insufficient data for the estimation of statistical effect size measures with confidence intervals; (7) Inter-study overlapping populations, determined by verifying the name and affiliation of authors, source of patients, and recruitment period. When results were derived from the same study population, the reports providing more complete datasets were included.

### 2.4. Study Selection Process

Eligibility criteria were blinded independently applied by both authors (PRG and MAGM). Any discrepancies were resolved by consensus. Articles were selected in two phases, first screening titles and abstracts for those apparently meeting inclusion criteria, and then reading the full text of selected articles, excluding those that did not meet the review eligibility criteria. Evaluators were first jointly trained and calibrated for the process of identification and selection of studies performing an initial screening round (50 papers each). An optimal inter-agreement proportional score (relative frequency of agreement = 97.94%) was finally obtained. The inter-rater reliability was also measured by calculating Cohen’s kappa statistic, obtaining an almost perfect agreement (κ = 0.96).

### 2.5. Data Extraction

Both authors (PRG and MAGM) independently extracted data from the selected articles after full-text reading, completing a data collection form in a standardized manner using the software Excel (v.16/2018, Microsoft, Redmond, WA, USA). Datasets extracted were secondarily jointly cross-checked, solving discrepancies by consensus. Data expressed as order statistics (i.e., median, interquartile range, and/or maximum-minimum values) were computed and transformed, if possible, into means ± standard deviation (SD) using the methods proposed by Luo et al., (2018) and Wan et al., (2014) [23,24]. If it was desirable to combine two or more different datasets expressed as means ±SD from subgroups into a single group, the Cochrane Handbook formula was applied [20]. Data were gathered on the first author, publication language, year, country and continent, sample size, anatomical site and subsites affected, sex and age of patients, tobacco and alcohol consumption, treatment modality, recruitment and follow up period, study design, methodology, and the frequency of proteins expression, immunohistochemical methods (i.e., anti-β-catenin antibody, dilution, incubation time and temperature), cut-off point, scoring system, subcellular β-catenin location and the relative frequency of cases presenting β-catenin aberrant expression (sub-categorized as loss of cell membrane, cytoplasmic-nuclear expression, or not defined in primary-level studies), expressed as proportions. Finally, the data required to analyse the outcomes were also recorded for clinicopathological (T status [T3/T4 vs. T1/T2], N status [N+ vs. N−], clinical-stage [III/IV vs. I/II] histological grade [II/III vs. I]) and prognostic main variables (OS and DFS). Furthermore, clinicopathological variables rarely reported in primary level studies were also ad hoc screened and categorized. We identified and extracted data on the relationships between β-catenin aberrant expression and the number of metastatic lymph nodes (multiple vs. single), extracapsular spread (extracapsular vs. intracapsular), tumour growth pattern (endophytic vs. exophytic), mode of invasion in tumour front (grades 3/4 vs. 1/2), perineural and lymphatic invasion (positive vs. negative).

### 2.6. Evaluation of Quality and Risk of Bias

Two authors (PRG and MAGM) critically appraised the methodological quality and risk of bias of primary-level studies using the Quality in Prognosis Studies (QUIPS) tool (developed by members of the Cochrane Prognosis Methods Group [25,26]). The following six potential bias domains were explored: (1) Study participation; (2) Study attrition; (3) Prognostic factor measurement; (4) Outcome measurement; (5) Study confounding; (6) Statistical analysis/reporting. The risk of bias was considered low, moderate, or high for each domain. Finally, an overall score was also estimated based on a method previously described by our research group [27]. In brief, low, moderate, or high overall risk of bias was assigned for each study—based on domains no. 3, and no. 5, considered as critical domains—with the purpose of statistically analysing the influence of the methodological quality of primary-level studies and impact on our meta-analytical results. Therefore, the quality of the evidence was also assessed and adjusted for the risk of bias.

### 2.7. Statistical Analysis

β-catenin aberrant expression was analysed as a dichotomous categorical variable according to the cut-off values adopted by primary-level studies. Odds ratios (OR) with their corresponding 95% confidence intervals (CI) were estimated and used as an effect size measure for the meta-analyses of the clinicopathological variables. Hazard ratios (HR) and 95%CI were used for the meta-analysis of prognostic variables due to their time-to-event nature [28]. When authors reported effect size metrics in their survival analyses, these were directly extracted from the primary-level studies. If HR and/or 95%CI were not explicitly provided by the authors, we calculated them using the methods described by Parmar et al. [29] and Tierney and colleagues [28]. When a study only reported survival curves, we extracted the data from Kaplan-Meier curves with Engauge Digitizer 4.1 software (open-source digitizing software developed by M. Mitchell). When HRs were determined in both univariable and multivariable models, data were extracted from the multivariable model, which reflects a greater adjustment for potentially confounding factors. All meta-analyses were conducted using the inverse-variance method under a random-effects model (based on the DerSimonian and Laird method). This approach was planned a priori in our study protocol, in order to account for the possibility that are different underlying results among study subpopulations (e.g., differences among the wide range of experimental immunohistochemical methods, differential β-catenin aberrant expression across subcellular locations, etc.). Forest plots were constructed to graphically represent the effect sizes and for subsequent visual inspection analysis (*p* < 0.05 was considered significant).

Heterogeneity between studies was assessed using the χ^2^-based Cochran’s Q test. Given the low statistical power of this test, *p* < 0.10 was considered significant. We also used Higgins I^2^ statistic to estimate what proportion of the variance in observed effects reflects variation in true effects, rather than sampling error. The percentage of inter-study heterogeneity was quantified considering values of 50–75% as a moderate-to-high degree of inconsistency [30,31]. Preplanned subgroup meta-analyses (by subcellular distribution, geographical area, immunohistochemical methods, and risk of bias) were performed to identify potential sources of heterogeneity and to potential study subpopulations. Furthermore, additional univariable random-effect meta-regression analyses were conducted, using the restricted maximum likelihood (REML) method, to explore the potential effect of additional study covariates (i.e., follow-up period, age, sex, and clinical stage) [32]. Considering the low number of studies with data available for meta-regression analyses, the *p*-values were re-calculated using a permutation test based on Monte Carlo simulations [33]. To obtain sufficient precision, the number of permutations was 10,000 [34]. Weighted bubble plots were also constructed to graphically represent the fitted meta-regression lines.

Furthermore, two additional analyses were carried out to test the stability and reliability of our meta-analytical results. First, sensitivity analyses were carried out to explore the influence of each primary-level study on the pooled overall estimates [35], repeating sequentially the meta-analyses, omitting one study at a time (“leave-one-out” method). Second, small-study effects analyses were carried out to identify potential biases, such as publication bias, constructing funnel plots, and using the Egger regression test (performing a linear regression of the effect estimates on their standard errors, weighting by 1/[variance of the effect estimate], considering a *p*_Egger_-value < 0.10 as significant) [36].

Finally, the meta-analysis of secondary clinicopathological parameters (i.e., number of metastatic lymph nodes, extracapsular spread, tumour growth pattern, mode of invasion in tumour front, perineural and lymphatic invasion) could not be performed due to the low number of observations extracted and a considerable degree of clinical and methodological heterogeneity. However, due to their potential prognostic implications, an albatross plot was constructed to graphically represent them [37], allowing an approximate examination of their underlying magnitudes of effect. Stata software was used for all statistical analyses (v.16.1, Stata Corp, College Station, TX, USA).

### 2.8. Validation of Methodological Quality

The methodology followed in this systematic review and meta-analysis was critically appraised and validated using “A MeaSurement Tool to Assess Systematic Reviews” AMSTAR2 checklist [21], created as an instrument to develop, evaluate, and validate high-quality systematic reviews and meta-analyses through 16 items [21]. An overall rating is obtained based on weaknesses in critical domains (i.e., items: 2, 4, 7, 9, 11, 13, and 15) and non-critical domains. The overall confidence on the methodology of a systematic review is rated in the following levels: “High”, “Moderate”, “Low”, and “Critically low” (the checklist and full explanation were included in Figure S28).

## 3. Results

### 3.1. Results of the Literature Search

**Figure 1:**
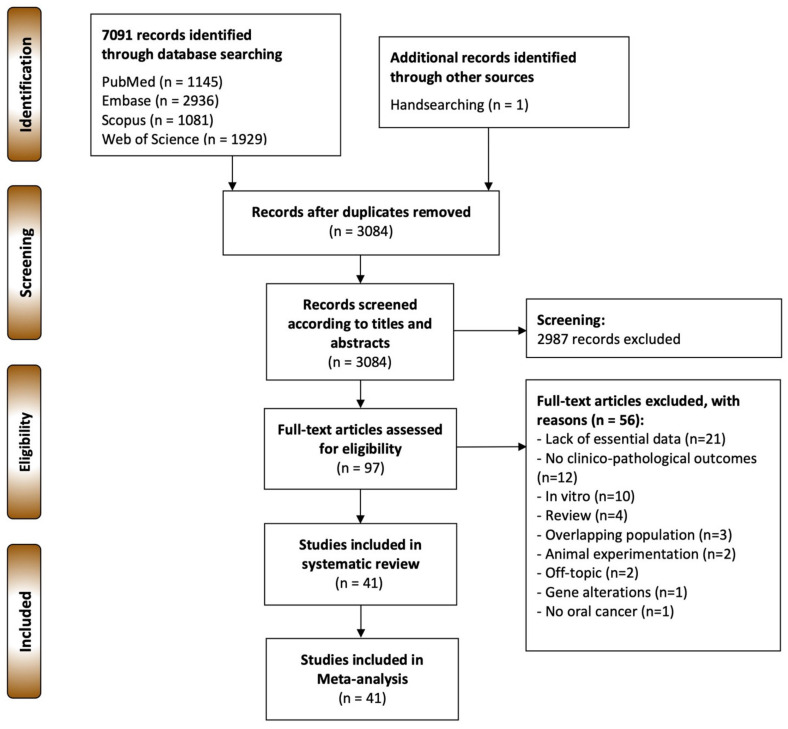
Flow diagram showing the identification and selection process of relevant studies, analysing the prognostic and clinicopathological significance of the aberrant expression of β-catenin in OSCC.

The flow diagram in Figure 1 depicts the study selection process and the results obtained. A total of 7091 publications were retrieved: 2936 from Embase, 1929 from Web of Science, 1145 from PubMed, 1081 from Scopus, and one from a screening of the reference lists. After duplicate removal, 3084 records were considered potentially eligible and their titles and abstracts were screened, leaving a sample of 97 papers for full-text evaluation (the studies excluded and their exclusion criteria were listed in the Supplementary Materials). Finally, 41 studies meeting all eligibility criteria were included for qualitative evaluation and meta-analysis [12,38,39,40,41,42,43,44,45,46,47,48,49,50,51,52,53,54,55,56,57,58,59,60,61,62,63,64,65,66,67,68,69,70,71,72,73,74,75,76,77].

### 3.2. Study Characteristics

Table 1 summarizes the main characteristics of our study sample, and Table S2 exhibits in detail the variables gathered from primary-level studies. These 41 studies recruited a total of 2746 patients, ranging between 12 and 290 patients. The prognostic value of the aberrant loss of β-catenin in the cell membrane was reported by 11 studies, the aberrant cytoplasmic-nuclear expression by 21 studies, and nine studies did not define the subcellular location investigated. All studies were observational retrospective cohorts (*n* = 41). In relation to the experimental methods used for the determination of β-catenin protein expression, all studies applied immunohistochemistry (*n* = 41), and Clone 14 was the anti-β-catenin antibody most frequently used (*n* = 6). Most studies processed their antibodies at dilutions equal or lower to 1:250 (*n* = 22), 15 studies incubated overnight (13 of them at 4 °C), while 13 studies 1 h at room temperature or higher. Finally, cut-off points were heterogeneous and varied widely across studies.

Table S2 exhibits in detail the characteristics of each primary-level study included in this systematic review and meta-analysis.

### 3.3. Qualitative Evaluation

**Figure 2:**
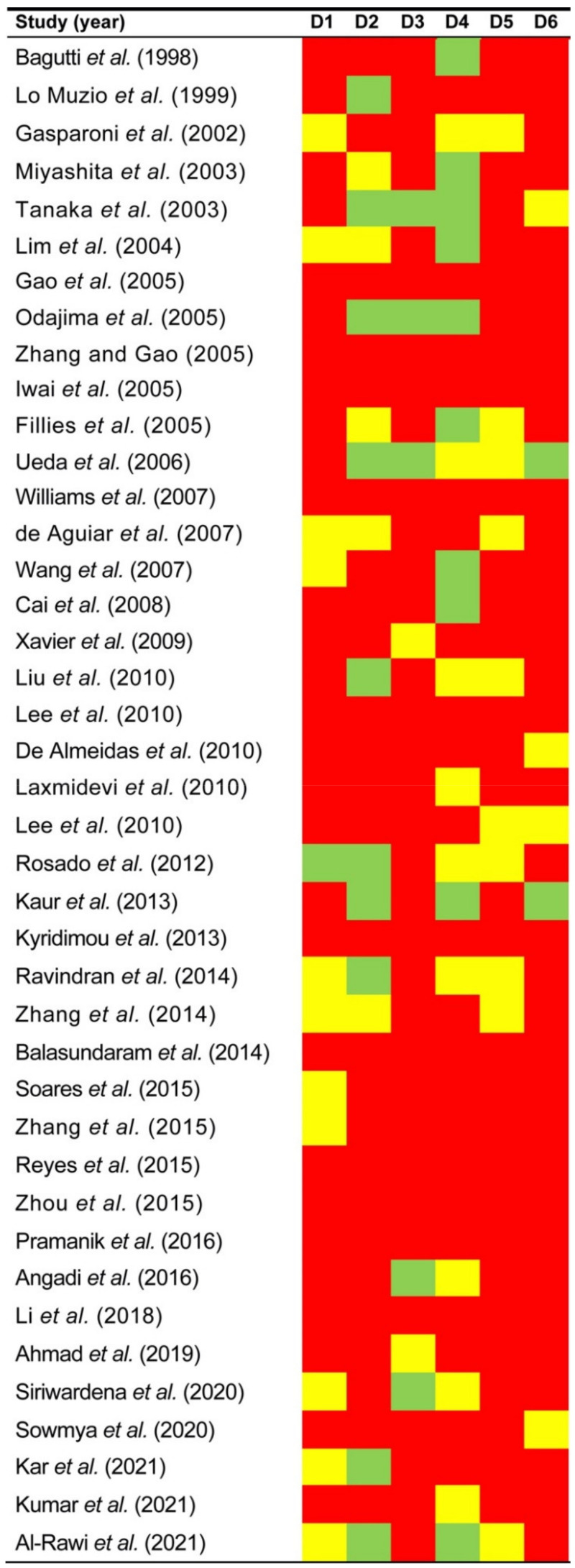
Evaluation of the risk of bias of primary-level studies [12,38,39,40,41,42,43,44,45,46,47,48,49,50,51,52,53,54,55,56,57,58,59,60,61,62,63,64,65,66,67,68,69,70,71,72,73,74,75,76,77] using the Quality in Prognosis Studies (QUIPS) tool. Green, low risk of potential bias; yellow, moderate; red, high.

The qualitative analysis was conducted using the QUIPS tool (Figure 2), which evaluates potential sources of bias in six domains.

#### 3.3.1. Study Participation

The risk of this bias was high in 70.73% of the reviewed studies, moderate in 26.83%, and low in 2.44%. Studies offering an inadequate description of their samples (sex and age of patients, oral cancer subsites, etc.) or setting (recruitment place and period) were considered as potentially biased.

#### 3.3.2. Study Attrition

The risk of this bias was high in 63.41% of the studies, moderate in 12.20%, and low in 24.39%. Some studies did not report essential information on the follow-up period (i.e., mean ± SD, median, IQR, and/or range). Only one study reported data on the patients’ drop-out rate [41], and none reported the attempt to collect information and reasons for patients lost to follow-up, or the description of their characteristics, which is essential to assess any differences with the characteristics of the final study sample.

#### 3.3.3. Prognostic Factor Measurement

The risk of this bias was high in 82.92% of the studies, moderate in 4.88%, and low in 12.20%. The most relevant potential bias was the lack of consideration of the β-catenin differential expression according to cell pattern distribution. It is essential to evaluate its prognostic value due to the well-known differential oncogenic roles linked to its translocation from membrane to cytoplasm and nucleus in cancer cells. Inappropriate design of cut points and scoring systems were also considered as serious sources of potential bias.

#### 3.3.4. Outcome Measurement

The risk of this bias was high in 53.66% of the studies, moderate in 21.95%, and low in 24.39%. The most frequent potential biases were the non-definition of survival parameters -relevant due to the lack of international consensus on survival endpoints in cancer research- and the failure to correctly report the classification system used (e.g., the edition of the AJCC/UICC TNM staging system, subject to periodic changes).

#### 3.3.5. Study Confounding

The risk of this bias was high in 75.61% of the studies, and moderate in 24.39%. The most frequent potential biases were the failure to consider confounders in the study design or to measure all potential confounders (e.g., tobacco or alcohol consumption). Unfortunately, as is often the case in studies on prognostic factors, no study defined a priori potential confounders or subsequently discussed their potential biological interactions between these covariates, β-catenin overexpression, and prognostic variables.

#### 3.3.6. Statistical Analysis and Reporting

The risk of this bias was high in 85.36% of the studies, moderate in 9.76%, and low in 4.88%. The most serious potential biases detected were inappropriate statistical analyses and obvious reporting errors, offering misleading results and conclusions. The most frequent biases were selective outcome reporting and the failure to estimate effect size measures with their corresponding 95%CI. Effect sizes (in this context, odds ratios, and hazard ratios) are much more informative than simple *p*-values, giving information on the magnitude, precision, and direction of the effect.

### 3.4. Quantitative Evaluation (Meta-Analysis)

All the variables considered for meta-analysis were graphically represented constructing forest plots (Figure 3 and Figure 4, Figures S1–S6 and S11–S15) and their results were listed in Table 2.

#### 3.4.1. Association between the Aberrant Expression of β-Catenin and Prognostic Variables

##### Overall Survival (OS)

Significant results were found for the aberrant expression of β-catenin and poor OS (HR = 1.77, 95% CI = 1.20–2.60, *p* = 0.004), although heterogeneity was present (*p* = 0.004, I^2^ = 62.4.3%). After the stratified meta-analysis by differential subcellular location, only the loss of cell membrane preserved the statistical association, showing a large effect size (HR = 2.37, 95% CI = 1.55–3.62, *p* < 0.001). Furthermore, the groups were more homogeneous in all the meta-analyses performed and the statistical heterogeneity was well-controlled, losing significance in cellular compartments (OS: *p* > 0.10, respectively).

##### Disease-Free Survival (DFS)

Significant results were found for the aberrant expression of β-catenin and poor DFS (HR = 2.44, 95% CI = 1.10–5.50, *p* = 0.03), although a considerable degree of heterogeneity was observed (*p* < 0.001, I^2^ = 88.6%). This result derived from a small sample size (*n* = 5 studies) and no subgroup meta-analysis was run, needing further investigation.

#### 3.4.2. Association between the Aberrant Expression of β-Catenin and Clinicopathological Variables

##### T Status

Significant results were found for the aberrant expression of β-catenin and T3/4-OSCCs (OR = 1.76, 95% CI = 1.23–2.53, *p* = 0.004). After the stratified meta-analysis by differential subcellular location, only the loss of cell membrane preserved again the statistical association (OR = 1.81, 95% CI = 1.05–3.11, *p* = 0.03).

##### N Status

A significant association was found among the aberrant expression of β-catenin and positive-lymph node metastasis (OR = 2.39, 95% CI = 1.68–3.40, *p* < 0.001). After the stratified meta-analysis by differential subcellular location, once again the loss of cell membrane preserved the statistical association, also showing a large effect size (OR = 3.44, 95% CI = 2.40–4.93, *p* < 0.001).

##### Clinical Stage

A significant association was found among the aberrant expression of β-catenin and advanced stage OSCCs (OR = 2.40, 95% CI = 1.58–3.63, *p* < 0.001). Both the loss of cell membrane (OR = 2.51, 95% CI = 1.17–5.35, *p* = 0.02) and the cytoplasmic-nuclear expression (OR = 3.12, 95% CI = 1.81–5.40, *p* < 0.001) preserved the statistical association.

##### Histological Grade

Although a significant association was for was found among the aberrant expression of β-catenin and moderately-poorly differentiated OSCCs, a reduced effect size was observed (OR = 1.57, 95% CI = 1.09–2.25, *p* = 0.02), and only the cytoplasmic-nuclear subgroup maintained this significant association (OR = 1.76, 95% CI = 1.23–2.53, *p* = 0.002).

### 3.5. Quantitative Evaluation (Variables Not Included in Meta-Analysis)

**Figure 5:**
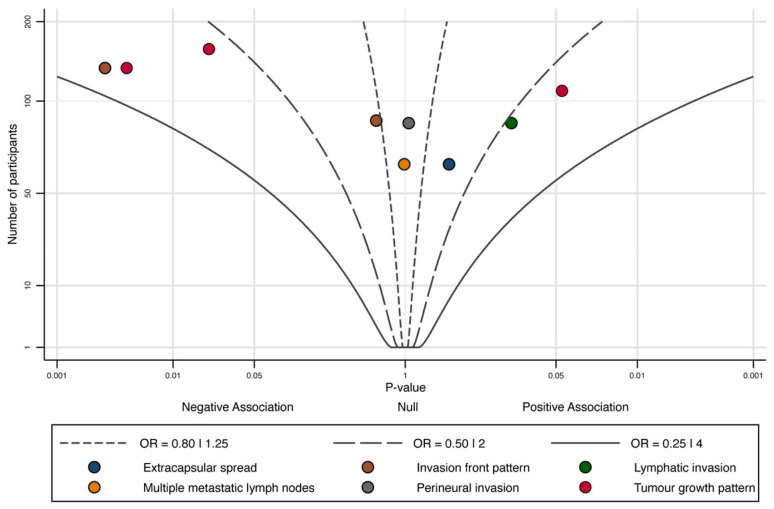
Albatross plot graphically representing the association between the aberrant expression of β-catenin and secondary clinicopathological parameters rarely reported in primary-level studies, but harbouring relevant prognostic implications. Every single study is represented by a circle of different colour, according to the clinicopathological parameter investigated (see legend). Two-sides *p*-values (horizontal x-axis) with results separated according to positive/negative association (i.e., the observed direction of effect) were plotted against the number of participants included within each study (vertical y-axis). The albatross plot allows a better interpretation of *p*-values from the variables that did not enter in the meta-analysis, in the context of the study sample sizes. Small studies lie toward the bottom of the plot and large studies toward the top. Effect contours (black continuous and intermittent lines) were drawn on the plot showing the ranges of the magnitudes of effect, using odds ratios (OR). A *p*-value < 0.05 was considered significant.

Meta-analysis was not performed for the association between the aberrant expression of β-catenin and the additional secondary variables (number of metastatic lymph nodes, extracapsular spread, tumour growth pattern, mode of invasion in tumour front, perineural and lymphatic invasion). However, all were included in an albatross plot (Figure 5) and considered separately in the narrative synthesis. All these variables were evaluated by a very low number of primary-level studies (*n* ≤ 3), showing imprecise and heterogeneous results. Only two variables showed a significant -but heterogeneous- statistical association with the aberrant expression of β-catenin (i.e., grade I,II-invasion pattern [*p* = 0.003; *n* = 1 study], endophytic- [*p* = 0.04; *n* = 1 study] and exophytic-tumour growth patterns [*p* = 0.02]). More investigation is needed to obtain a better quality of evidence on these parameters and their relationships with the aberrant expression of β-catenin.

### 3.6. Quantitative Evaluation (Secondary Analyses)

#### 3.6.1. Analysis of Subgroups

Some additional subgroups maintained the precedent significant association between the aberrant expression of β-catenin and OS after stratify by anti-β-catenin antibody (Clone-14: HR = 3.57, 95% CI = 2.03–6.29, *p* < 0.001), by anti-β-catenin antibody dilution (<1:250: HR = 2.81, 95% CI = 1.87–4.22, *p* < 0.001; 1:500–1000: HR = 1.65, 95% CI = 1.10–2.47, *p* = 0.02), by incubation time and temperature (overnight at 4 °C: respectively, HR = 2.31, 95% CI = 1.65–3.23, *p* < 0.001) and by overall RoB (low RoB: HR = 3.28, 95% CI = 2.05–5.24, *p* < 0.001) (Table 2, Figures S1–S6).

#### 3.6.2. Meta-Regression Analysis

Meta-regression was also performed to explore the potential effect of the study covariates sex, age, clinical stage and follow up on the relationships between the aberrant expression of β-catenin and OS (Table 2, Figures S7–S10, Supplementary Materials). Only one significant association was found (i.e., older patients presenting aberrant β-catenin expression showed the worst prognosis, *p* = 0.02), based on a low number of observations with imprecise results, reaching the statistical significance after recalculating the *p*-value through Monte Carlo simulations (10,000 permutations). More investigation is also needed to obtain a better quality of evidence on this result.

#### 3.6.3. Sensitivity Analysis

In general, the overall results did not substantially vary after the sequential repetition of meta-analyses, omitting one study each time (“leave-one-out” method) (Figures S22–S27). This suggests that the pooled ratio metrics (i.e., HRs and ORs) reported in this meta-analysis do not depend on the influence of a particular individual primary-level study, reaffirming the stability of our results.

#### 3.6.4. Analysis of Small-Study Effects

Visual inspection analysis of the asymmetry of the funnel plots constructed and the statistical tests conducted for the same purpose confirm the absence of small-study effects on the variables T status (p_Egger_ = 0.72), clinical-stage (p_Egger_ = 0.44), and histological grade (p_Egger_ = 0.40) (Figures S18, S20 and S21). On the other hand, small-study effects were present on the variables OS (p_Egger_ = 0.05), DFS (p_Egger_ = 0.02), and N status (p_Egger_ = 0.05) (Figures S16, S17 and S19), for which biases, e.g., publication bias, could not be ruled out.

### 3.7. Validation of Methodological Quality

The methods applied in this systematic review and meta-analysis were implemented, critically appraised, and validated using AMSTAR2 (Figure S28) [21], obtaining an overall rating of “high” (16 points) (the scoring table was included in Table S3).

## 4. Discussion

The results of our systematic review and meta-analysis of 41 studies and 2746 patients with OSCC show that aberrant expression of β-catenin (loss of membrane expression, cytoplasmic expression, and/or nuclear expression) is a poor prognostic factor in OSCC associated with a significant decrease in overall survival (*p* = 0.004) and disease-free survival (*p* = 0.03). The detailed analysis of these results interestingly points out that the determining fact affecting survival in this tumour is the loss of β-catenin membrane expression (*p* < 0.001), presenting also a high magnitude of the effect (HR > 2) (Table 2). Our meta-analysis confirms the observation of our previous primary level study on OSCC patients in which we also reported that the oncogenic actions of β-catenin developed essentially as a consequence of the loss of its membrane expression [17]. The loss of β-catenin expression in the membrane of oral squamous epithelial cells implies a loss of cell adhesion and an invasiveness gain, being necessary for the loss of E-cadherin membrane expression, its natural partner. Different processes can result in the β-catenin membrane loss expression. As previously discussed, activation of the canonical Wnt oncogenic pathway not only leads to the loss of β-catenin in the membrane but also promotes its cytoplasmic accumulation enabling the translocation to the nucleus where it acts as an oncogene transcription factor. However, it has also been documented that the state of β-catenin membrane expression may be independent of the activation of the Wnt canonical pathway [3]. Loss of E-cadherin expression by mutations of its gene, CDH1, very rare [78], or by epigenetic mechanisms linked to CDH1 promoter methylation, probably more relevant [79,80], also involve membranous loss of β-catenin. It should be emphasized that the alterations E-cadherin/β-catenin complex in the cell membrane are a key fact for the EMT phenomenon development, whereby oral epithelial cells change their polygonal squamous morphology and acquire fibroblast or myofibroblast-like appearance with an expression of mesenchymal markers (vimentin, α-SMA, and FSP1), increase their motility and invasive capacity, and acquire cancer stem cell characteristics [3,8]. In addition to EMT, two related concepts –partial EMT and anaplastic transition- could also be explained by the alterations of E-cadherin/β-catenin complexes in the cell membrane of the epithelial surface and the poor prognosis of the patients with OSCC. It has recently been proposed that, rather than being a binary process, EMT occurs through distinct intermediate states, and cancer cells may acquire one or more hybrid epithelial/mesenchymal phenotypes during EMT, a phenomenon also known as “partial EMT”, exhibiting a mixture of epithelial and mesenchymal characteristics at molecular and morphological level [81]. This phenomenon has also been demonstrated in squamous cell carcinomas by several studies including OSCCs [82,83], where the immunohistochemical detection of E-cadherin expression has also been assessed to confirm the presence and prognostic implications of partial EMT [83]. On the other hand, a novel concept referred to as anaplastic transition has been proposed in OSCC, also associated with poor prognosis [84]. In the anaplastic transition, the epithelial cancer cells seem to dedifferentiate into more primitive states concurrently presenting epithelial and mesenchymal features. In this phenomenon, the loss of E-cadherin was also a frequent finding, together with other molecular mechanisms not associated with the EMT phenomenon, such as the concomitant presentation of cytokeratin 14 [84]. Future studies should elucidate the implications of β-catenin in the context of partial EMT and anaplastic transition in OSCC. Some results of our meta-analysis underline that indeed, as a consequence of the gain in cell motility and invasiveness linked to the membrane loss of β-catenin, clinicopathologic events of OSCC occur that decisively affect the prognosis. It is relevant to highlight the association we have found between the loss of membranous β-catenin and lymph node involvement by the tumour (N status) (*p* < 0.001) with the magnitude of the effect being the highest of those found in our meta-analysis (OR = 3.44). In other words, the main negative effect on the survival of patients with OSCC exerted by the loss of β-catenin expression in the cell membrane came from the increased chances of developing lymph node metastases as a consequence of the gain in motility and invasiveness acquired by the malignant cells. Likewise, the clinical tumour stage is also significantly increased in those cases with the absence of β-catenin in the membrane (*p* = 0.02). Finally, we observed that OSCC with loss of β-catenin in membrane also had larger sizes (T status) (*p* = 0.03) which is probably related to an increase in cell proliferative activity linked to the activation of the Wnt canonical pathway and to the consequent functions on β-catenin which then behaves as an oncogenic transcription factor inducing increased cell proliferative activity [3,85,86,87]. However, it is striking that nuclear/cytoplasmic expression of β-catenin has not been shown by itself in this meta-analysis to be significantly associated with poor survival, although a marked trend of effect is shown (*p* = 0.07); this result is probably due to the paucity of primary studies addressing this question (3 studies, 255 patients). A conclusion of our work should therefore be that more primary-level studies are needed to allow, based on evidence, to clarify the prognostic value of cytoplasmic/nuclear β-catenin overexpression.

Our meta-analysis also shows that the way in which the immunohistochemical technique is developed also affects survival outcomes. This seems relevant since the value acquired by the aberrant expression of β-catenin as a marker of survival in patients with OSCC is achieved with efficiency if certain principles are followed in the development of the technique which has been, according to the results of our study, those that have shown the greatest productivity. In this sense, the monoclonal antibody that has proved to be the most efficient was Clone-14, with a dilution <1:250, incubated overnight at 4 °C (*p* < 0.001, respectively).

Many other molecular biomarkers (such as SCC antigen, CYFRA 21-1, CEA, TPS, or *TP53*/p53, EGFR, etc.) have also been reported in head and neck carcinogenesis showing promising diagnostic, prognostic, and therapeutic implications [88,89,90]. Nevertheless, through a systematic review and meta-analysis (a study design to assess and synthetize the quality of evidence, providing a higher knowledge) there is a more limited set of prognostic biomarkers in OSCC (e.g., *CCND1*/cyclin D1 [91], survivin [92], podoplanin [93], *CTTN*/cortactin [94], PD-L1 [95], tyrosine kinase receptors like ErbB2 [96], or members of PI3K signalling pathway like Akt or mTor [97]). Several of these biomarkers are closely related to β-catenin oncogenic roles, for example, the activation of Wnt canonical pathway is considered as one of the main mechanisms of cyclin D1 overexpression in OSCC [3,85]; while surviving [98], podoplanin [99], and cortactin [100] also seem to play important roles during EMT phenomenon in OSCC, mainly in the context of cytoskeleton oncogenic dysregulation [101,102]. Nevertheless, the relationship between β-catenin and these biomarkers is not well supported by primary-level studies offering a high quality of evidence, and these biomarkers should be better investigated jointly in patients with OSCC. Hypothetically, their combined effect sizes could be synergistic, even reaching a better prognostic value than as individual tools. Therefore, future observational studies should be conducted for this purpose, preferably prospective cohorts, by estimating hazard ratios with their corresponding confidence intervals and multivariable-adjusted, accounting for potentially confounding factors.

According to our qualitative evaluation using the QUIPS tool, we also should point out that the studies included in this systematic review and meta-analysis have not been conducted with the same methodological rigor, most of them presenting a high risk of potential bias across several domains. After applying a subgroup meta-analysis to assess the influence of these studies on the overall results, only the subset of studies with lower risk of bias (i.e., higher methodological quality and internal validity) preserved the statistically significant association between aberrant β-catenin expression and poor survival, showing an even higher magnitude of effect (HR > 3, *p* < 0.001). This result indicates that the effect size reported –association between the aberrant expression of β-catenin and survival- is probably underestimated, which should be demonstrated through the publication of new studies carefully designed which should consider the potential biases and recommendations reported in this systematic review and meta-analysis, to improve and standardize future research.

Some potential limitations of our study should also be discussed. First, some primary-level studies included in our sample did not clearly define the subcellular location of β-catenin of the cases included in their cohorts (9 out of 41, 21.95%). Consequently, this was the only subgroup that did not preserve the statistical significance in the prognostic variables investigated. This fact could hypothetically be due to the fact that these studies may have also considered as a positive expression the overexpression of β-catenin in the cell membrane, where it exerts a physiological function, safeguarding cell-cell adhesions and homeostasis in squamous epithelia. It could logically contaminate the results of studies that aim to evaluate the value of this adhesion molecule as a prognostic biomarker in oral cancer. A challenging alternative in the design of this meta-analysis might have been the exclusion of these studies that do not clearly define the subcellular location of β-catenin. Nevertheless, this would not have allowed us to recognize that the lack of clarification on the cellular location of β-catenin affects the prognostic value that this protein exerts on oral cancer. Preserving this subset of studies in which the subcellular location of β-catenin is not determined has allowed us to make the recommendation for future studies to be strict during the reporting of the aberrant differential expression of β-catenin (i.e., separately, the cell membrane loss, cytoplasmic and nuclear expression). Furthermore, these datasets should preferably be reported through individual patient data, instead of aggregated summary data, which would allow for a more in-depth and detailed adjusted analysis. A second potential limitation that should be discussed is the presence of inter-study heterogeneity in the overall results of our meta-analysis on survival. We a priori designed the implementation of random-effects models, as methodological and clinical heterogeneity was expected. Nevertheless, statistical heterogeneity completely disappeared after the stratified meta-analysis in more homogeneous subgroups by β-catenin expression in subcellular locations. Therefore, heterogeneity should not be truly considered as a limitation of the present meta-analysis. Third, a meta-analysis could not be performed for several relevant clinicopathological variables that are rarely reported in primary level studies (e.g., number of metastatic lymph nodes, extracapsular spread, invasion front behaviour, perineural and lymphatic invasion, etc.). Although we tried to consider them separately in an albatross plot and through narrative synthesis, the results were sparse and very imprecise. Future studies should also make a greater effort in the collection of these secondary clinicopathological variables of interest, where the aberrant expression of β-catenin should also be better analysed as a potential prognostic biomarker. A final potential limitation frequently encountered in the literature on prognostic biomarkers in cancer is the tendency to publish only positive results [103], and our statistical analyses confirmed the presence of funnel plot asymmetry, not allowing us to rule out publication bias. Despite the above limitations, our study is robust, presenting the first meta-analysis on this topic, reporting relevant and powerful results derived from the largest sample size analysed to date (*n* = 41 studies/2746 patients), as well as raising important methodological recommendations for the design of future studies.

## 5. Conclusions

In conclusion, our systematic review and meta-analysis demonstrates on the basis of evidence that loss of β-catenin expression in the membrane of tumour cells behaves as a marker of poor survival in OSCC, which is essentially linked to the increased risk for the development of lymph node metastases in these patients. In our opinion, the assessment of membranous expression of β-catenin could be incorporated as an additional and complementary routine marker for the prognostic assessment of patients with OSCC. Likewise, we believe that more primary-level studies are needed to evaluate on the basis of evidence what is the prognostic value of cytoplasmic/nuclear overexpression of β-catenin in OSCC patients.

## Figures and Tables

**Figure 3:**
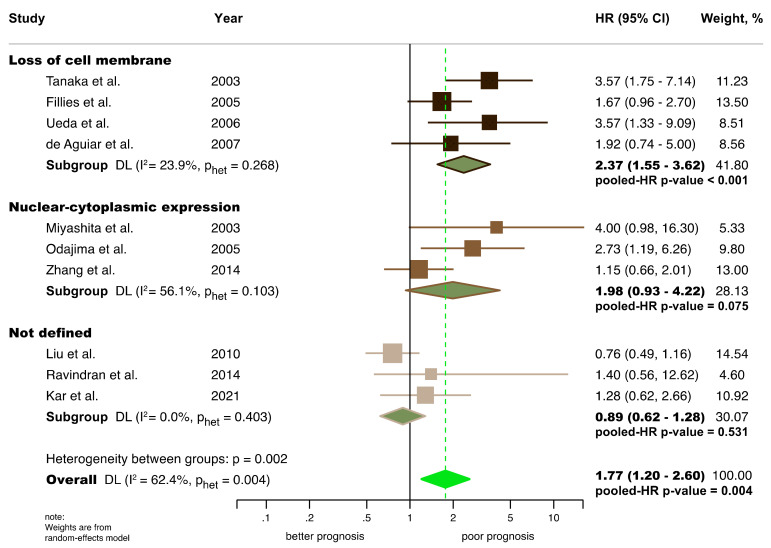
Forest plot of the association between the aberrant expression of β-catenin and overall survival in OSCC (random-effect model meta-analysis, inverse-variance weighting based on the DerSimonian and Laird method), stratified by subcellular location (dark brown, loss of cell membrane; medium brown, nuclear-cytoplasmic expression; light brown, not defined in primary level studies; green, overall pooled estimates). Ten primary-level studies were meta-analysed for this variable [40,41,42,46,55,56,69,71,73,76]. A HR > 1 suggests that the aberrant expression of β-catenin is associated with poor overall survival. Diamonds indicate overall HR with their corresponding 95% CIs. HR, hazard ratio; CI, confidence intervals.

**Figure 4:**
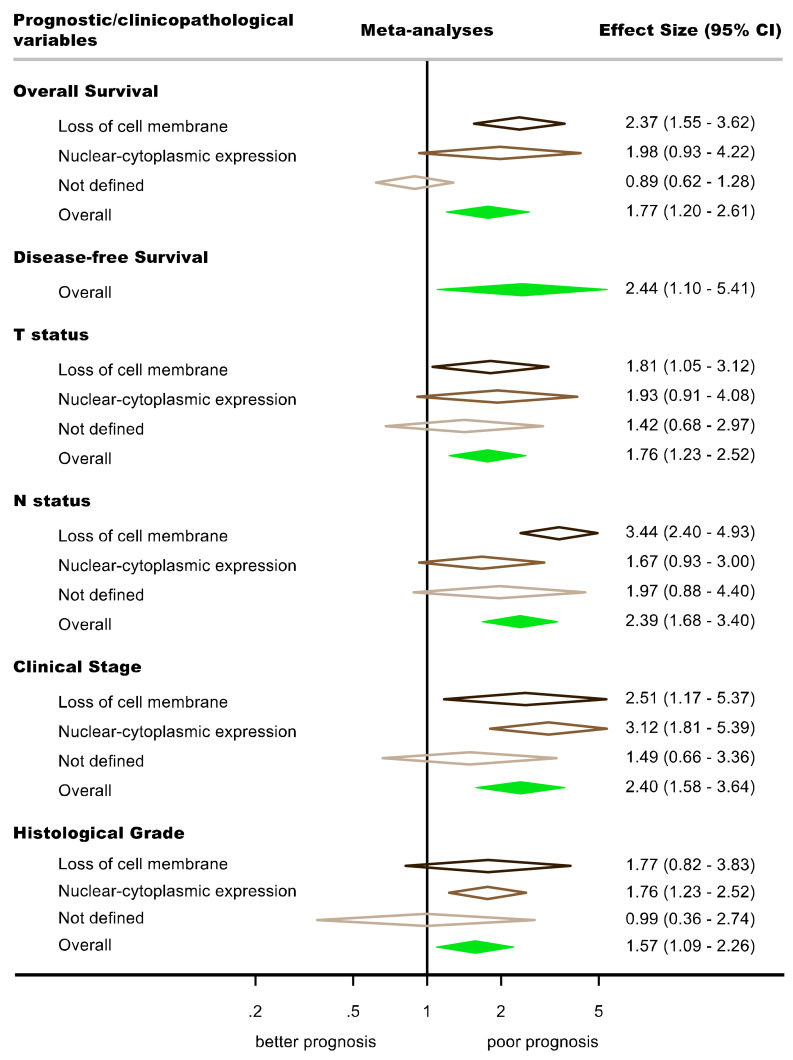
Summary Forest Plot (aka forest top plot) graphically representing the effect sizes (measured as ratio metrics, i.e., hazard ratios for prognostic survival variables, and odds ratios for clinicopathological parameters) of the aberrant expression of β-catenin in oral squamous cell carcinoma. Each row displays the different meta-analytical results (*n* = 21), performed in this study, stratified by subcellular location (dark brown, loss of cell membrane; medium brown, nuclear-cytoplasmic expression; light brown, not defined in primary level studies; green, overall pooled estimates). Random-effects model meta-analyses, inverse-variance weighting based on the DerSimonian and Laird method. An effect size >1 suggests that the aberrant expression of β-catenin is associated with a poor prognosis. Diamonds graphically represent pooled effect sizes with their corresponding 95% confidence intervals (CI).

**Table 1:** Summarized characteristics of the study sample.

Total	41 Studies
Year of publication	1998–2021
Total patients (range)	2746 (12–290)
β-catenin aberrant subcellular location	
Cell membrane loss	11 studies
Cytoplasmic-nuclear expression	21 studies
Not defined in primary-level studies	9 studies
Study design	
Retrospective cohort	41 studies
Experimental methods for β-catenin expression determination	
Immunohistochemistry	41 studies
Anti-β-catenin antibody	
Clone 14	6 studies
sc-7963	2 studies
C19220	2 studies
Other	10 studies
Not reported	21 studies
Anti-β-catenin antibody dilution	
<1:250	22 studies
1:500–1000	7 studies
Not reported	12 studies
Anti- β-catenin antibody incubation time	
Overnight	15 studies
1 h	13 studies
Not reported	13 studies
Anti-β-catenin antibody incubation temperature	
4 °C	13 studies
Room temperature or higher	13 studies
Not reported	15 studies
Geographical region	
Asian countries	29 studies
Non-Asian countries	12 studies

**Table 2:** Meta-analyses of the prognostic and clinicopathological significance of aberrant β-catenin expression in OSCC.

Meta-Analyses	No. of Studies	No. of Patients	Stat. Model	Wt	Pooled Data		Heterogeneity		Supplementary Materials ^a^
					ES (95% CI)	*p*-Value	*P_het_*	I^2^ (%)	
Survival Parameters									
**Overall Survival**									
Aberrant β-catenin expression (all) ^b^	10	938	REM	DL	HR = 1.77 (1.20–2.60)	0.004	0.004	62.4	Manuscript, Figure 3
Subgroup analysis by differential subcellular location ^c^									Figure 3
Loss of cell membrane	4	460	REM	DL	HR = 2.37 (1.55–3.62)	<0.001	0.27	23.9	
Nuclear-cytoplasmic expression	3	255	REM	DL	HR = 1.98 (0.93–4.22)	0.07	0.10	56.1	
Not defined in primary-level studies	3	223	REM	DL	HR = 0.89 (0.62–1.28)	0.53	0.40	0.0	
Subgroup analysis by geographical area ^c^									Figure S1
Asian	8	772	REM	DL	HR = 1.81 (1.10–3.00)	0.02	0.001	70.1	
Non-Asian	2	166	REM	DL	HR = 1.72 (1.09–2.71)	0.02	0.80	0.0	
Subgroup analysis by anti-β-catenin antibody ^c^									Figure S2
Clone-14	2	294	REM	DL	HR = 3.57 (2.03–6.29)	<0.001	1.00	0.0	
Sc-7963	2	143	REM	DL	HR = 0.79 (0.52–1.20)	0.27	0.46	0.0	
Other	2	190	REM	DL	HR = 1.82 (0.87–3.81)	0.11	0.18	44.7	
Not reported	4	311	REM	DL	HR = 1.56 (1.10–2.22)	0.01	0.37	3.9	
Subgroup analysis by anti-β-catenin antibody dilution ^c^									Figure S3
<1:250	5	545	REM	DL	HR = 2.81 (1.87–4.22)	<0.001	0.72	0.0	
1:500–1000	3	230	REM	DL	HR = 1.65 (1.10–2.47)	0.02	0.37	0.0	
Not reported	2	163	REM	DL	HR = 0.90 (0.60–1.34)	0.60	0.25	26.0	
Subgroup analysis by anti-β-catenin antibody incubation time ^c^									Figure S4
1 h	3	273	REM	DL	HR = 1.23 (0.64–2.36)	0.53	0.03	72.9	
Overnight	6	585	REM	DL	HR = 2.31 (1.65–3.23)	<0.001	0.43	0.0	
Not reported	1	80	-	-	HR = 1.28 (0.62–2.65)	0.51	-	-	
Subgroup analysis by anti-β-catenin antibody incubation temperature ^c^									Figure S5
4 °C	6	585	REM	DL	HR = 2.31 (1.65–3.23)	<0.001	0.43	0.0	
Room temperature or higher	3	273	REM	DL	HR = 1.23 (0.64–2.36)	0.53	0.03	72.9	
Not reported	1	80	-	-	HR = 1.28 (0.62–2.65)	0.51	-	-	
Subgroup analysis by overall risk of bias in primary-level studies ^c^									Figure S6
Low RoB	3	404	REM	DL	HR = 3.28 (2.05–5.24)	<0.001	0.87	0.0	
Moderate-High RoB	7	534	REM	DL	HR = 1.29 (0.91–1.83)	0.15	0.14	38.5	
Univariable meta-regressions by study design and patients’ characteristics ^d^									
Follow up (months, mean)	7	618	random-effects meta-regression		Coef = 0.016 (−0.033 to 0.065)	0.47 ± 0.005 ^e^	het_explained_ = −1.02% ^f^		Figure S7
Sex (proportion of males, %)	10	938	random-effects meta-regression		Coef = −0.005 (−0.035 to 0.024)	0.69 ± 0.005 ^e^	het_explained_ = −17.95% ^f^		Figure S8
Age (years, mean)	9	858	random-effects meta-regression		Coef = 0.132 (0.034 to 0.229)	0.02 ± 0.002 ^e^	het_explained_ = 79.62% ^f^		Figure S9
Clinical stage (proportion of stage-III/IV patients, %)	5	469	random-effects meta-regression		Coef = 0.001 (−0.078 to 0.079)	0.99 ± 0.001 ^e^	het_explained_ = −38.30% ^f^		Figure S10
Tobacco consumption (proportion of smokers, %)	2	141	random-effects meta-regression		-	-	-		-
Areca nut/Betel quid consumption (proportion of chewers, %)	0	0	-		-	-	-		-
Alcohol consumption (% of patients with positive habits)	2	141	-		-	-	-		-
**Disease-free survival**									
Aberrant β-catenin expression (all) ^b^	5	379	REM	DL	HR = 2.44 (1.10–5.40)	0.03	<0.001	88.6	Figure S11
**Clinicopathological Characteristics**									
**T Status**									
Aberrant β-catenin expression (all) ^b^	10	1418	REM	DL	OR = 1.76 (1.23–2.53)	0.004	0.06	37.1	Figure S12
Subgroup analysis by differential subcellular location ^c^									Figure S12
Loss of cell membrane	7	673	REM	DL	OR = 1.81 (1.05–3.11)	0.03	0.08	47.7	
Nuclear-cytoplasmic expression	7	542	REM	DL	OR = 1.93 (0.91–4.06)	0.09	0.05	53.0	
Not defined in primary-level studies	4	203	REM	DL	OR = 1.42 (0.68–2.97)	0.35	0.50	0.0	
**N Status**									
Aberrant β-catenin expression (all) ^b^	23	1881	REM	DL	OR = 2.39 (1.68–3.40)	<0.001	0.002	53.0	Figure S13
Subgroup analysis by differential subcellular location ^c^									Figure S13
Loss of cell membrane	9	769	REM	DL	OR = 3.44 (2.40–4.93)	<0.001	0.44	0.0	
Nuclear-cytoplasmic expression	9	849	REM	DL	OR = 1.67 (0.93–3.00)	0.08	0.01	59.1	
Not defined in primary-level studies	5	263	REM	DL	OR = 1.97 (0.88–4.38)	0.10	0.18	36.7	
**Clinical Stage**									
Aberrant β-catenin expression (all) ^b^	15	1165	REM	DL	OR = 2.40 (1.58–3.63)	<0.001	0.03	45.6	Figure S14
Subgroup analysis by differential subcellular location ^c^									Figure S14
Loss of cell membrane	6	514	REM	DL	OR = 2.51 (1.17–5.35)	0.02	0.01	66.7	
Nuclear-cytoplasmic expression	5	426	REM	DL	OR = 3.12 (1.81–5.40)	<0.001	0.50	0.0	
Not defined in primary-level studies	4	225	REM	DL	OR = 1.49 (0.66–3.36)	0.33	0.21	33.5	
**Histological Grade**									
Aberrant β-catenin expression (all) ^b^	32	1974	REM	DL	OR = 1.57 (1.09–2.25)	0.02	<0.001	55.1	Figure S15
Subgroup analysis by differential subcellular location ^c^									Figure S15
Loss of cell membrane	7	604	REM	DL	OR = 1.77 (0.82–3.83)	0.14	0.002	71.9	
Nuclear-cytoplasmic expression	17	986	REM	DL	OR = 1.76 (1.23–2.53)	0.002	0.33	10.2	
Not defined in primary-level studies	8	384	REM	DL	OR = 0.99 (0.36–2.76)	0.99	<0.001	73.4	

Abbreviations: Stat., statistical; Wt, method of weighting; ES, effect size estimation; HR, hazard ratio; OR, odds ratio; CI, confidence intervals; REM, random-effects model; DL, DerSimonian and Laird method; OSCC, oral squamous cell carcinoma; RoB, risk of bias; ^a^—More information in the Supplementary Materials, ^b^—Prognostic meta-analysis of aggregate (summary) data, ^c^—Subgroup meta-analyses, ^d^—Meta-regression analysis of the potential effect of study covariates on the association between the aberrant expression of β-catenin and OSCC. A meta-regression coefficient > 0 indicates a greater impact of covariates on poor prognosis., ^e^—*p*-value ± standard error recalculated after 10,000 permutations based on Monte Carlo simulations, ^f^—Proportion of between-study variance explained (adjusted R^2^ statistic) using the residual maximum likelihood (REML) method. A negative number for a proportion of heterogeneity explained reflects no heterogeneity explained.

## Supplementary Materials

The following supporting information can be downloaded at: https://www.mdpi.com/article/10.3390/cancers14030479/s1, Figure S1: Forest plot graphically representing the stratified analysis by geographical area on the association between the aberrant β-catenin expression and overall survival in patients with OSCC, Figure S2: Forest plot graphically representing the stratified analysis by anti-β-catenin antibody on the association between the aberrant β-catenin expression and overall survival in patients with OSCC, Figure S3: Forest plot graphically representing the stratified analysis by anti-β-catenin antibody dilution on the association between the aberrant β-catenin expression and overall survival in patients with OSCC, Figure S4: Forest plot graphically representing the stratified analysis by anti-β-catenin antibody incubation time on the association between the aberrant β-catenin expression and overall survival in patients with OSCC, Figure S5: Forest plot graphically representing the stratified analysis by anti-β-catenin antibody incubation temperature on the association between the aberrant β-catenin expression and overall survival in patients with OSCC, Figure S6: Forest plot graphically representing the stratified analysis by overall RoB in primary-level studies, on the association between the aberrant β-catenin expression and overall survival in patients with OSCC, Figure S7: Bubble plot graphically representing the univariable meta-regression analysis of the potential effect of follow up period (expressed in months, in x-axis) on the association between the aberrant β-catenin expression and overall survival in patients with OSCC (using HR as effect size measure, in y-axis), Figure S8: Bubble plot graphically representing the univariable meta-regression analysis of the potential effect of sex (% of males) on the association between the aberrant β-catenin expression and overall survival in patients with OSCC, Figure S9: Bubble plot graphically representing the univariable meta-regression analysis of the potential effect of age (mean age of patients, expressed in years) on the association between the aberrant β-catenin expression and overall survival in patients with OSCC, Figure S10: Bubble plot graphically representing the univariable meta-regression analysis of the potential effect of clinical stage (% of stage III/IV patients) on the association between the aberrant β-catenin expression and overall survival in patients with OSCC, Figure S11: Forest plot graphically representing the stratified analysis on the association between the aberrant β-catenin expression and DFS in patients with OSCC, Figure S12: Forest plot graphically representing the stratified analysis by anti-β-catenin subcellular location on the association between the aberrant β-catenin expression and T status (T3/T4 vs. T1/T2) in patients with OSCC, Figure S13: Forest plot graphically representing the stratified analysis by anti-β-catenin subcellular location on the association between the aberrant β-catenin expression and N status (positive metastatic lymph nodes vs. negative) in patients with OSCC, Figure S14: Forest plot graphically representing the stratified analysis by anti-β-catenin subcellular location on the association between the aberrant β-catenin expression and clinical stage (III/IV vs. I/II) in patients with OSCC, Figure S15: Forest plot graphically representing the stratified analysis by anti-βcatenin subcellular location on the association between the aberrant β-catenin expression and histological grade (poorly-moderate vs. well-differentiated carcinomas) in patients with OSCC, Figure S16: A funnel plot of estimated logHRs against their standard errors, graphically representing the analysis of small-study effects on the association between the aberrant β-catenin expression and overall survival in OSCC, Figure S17: A funnel plot of estimated logHRs against their standard errors, graphically representing the analysis of small-study effects on the association between the aberrant β-catenin expression and DFS in OSCC, Figure S18: A funnel plot of estimated logORs against their standard errors, graphically representing the analysis of small-study effects on the association between the aberrant β-catenin expression and T status in OSCC, Figure S19: A funnel plot of estimated logORs against their standard errors, graphically representing the analysis of small-study effects on the association between the aberrant β-catenin expression and N status in OSCC, Figure S20: A funnel plot of estimated logORs against their standard errors, graphically representing the analysis of small-study effects on the association between the aberrant β-catenin expression and clinical stage in OSCC, Figure S21: A funnel plot of estimated logORs against their standard errors, graphically representing the analysis of small-study effects on the association between the aberrant β-catenin expression and histological grade in OSCC, Figure S22: Interval plot graphically representing the sensitivity analysis of the studies pooled in the meta-analysis on the association between aberrant β-catenin expression and overall survival in OSCC, Figure S23: Interval plot graphically representing the sensitivity analysis of the studies pooled in the meta-analysis on the association between aberrant β-catenin expression and DFS in OSCC, Figure S24: Interval plot graphically representing the sensitivity analysis of the studies pooled in the meta-analysis on the association between aberrant β-catenin expression and T status in OSCC, Figure S25: Interval plot graphically representing the sensitivity analysis of the studies pooled in the meta-analysis on the association between aberrant β-catenin expression and N status in OSCC, Figure S26: Interval plot graphically representing the sensitivity analysis of the studies pooled in the meta-analysis on the association between aberrant β-catenin expression and clinical stage in OSCC, Figure S27: Interval plot graphically representing the sensitivity analysis of the studies pooled in the meta-analysis on the association between aberrant β-catenin expression and histological grade in OSCC, Figure S28: AMSTAR2 checklist, Table S1: Search strategy for each database, number of results, and execution date, Table S2: Characteristics of analyzed studies (*n* = 41), Table S3: AMSTAR2 scoring system. Table S4: Stage of review at the date of protocol preparation (October 2020).
