# Supplementary material for: Prognostic and Clinicopathological Significance of the Aberrant Expression of β-Catenin in Oral Squamous Cell Carcinoma: A Systematic Review and Meta-Analysis
Source: Cancers (Basel). 2022 Jan 18;14(3):479. doi: 10.3390/cancers14030479 (PMC8833491; doi:10.3390/cancers14030479)
Supplement: Supplementary file 1 [file cancers-14-00479-s001.zip › cancers-1552740-supplementary.pdf]

Supplementary Materials

Prognostic and Clinicopathological Significance of the Aberrant Expression of β-Catenin in Oral Squamous Cell Carcinoma: A Systematic Review and Meta-Analysis

Pablo Ramos-García and Miguel Á. González-Moles

1. Search Strategy

Table S1. Search strategy for each database, number of results, and execution date.

Database	Query/Search Strategy	Results/Items Found	Search Time Limits
PubMed	("beta Catenin"[Mesh Terms] OR "β-catenin"[All Fields] or "beta-catenin"[All Fields] or "b-catenin"[All Fields] OR "CTNNB"[All Fields] OR "Wnt Signaling Pathway"[Mesh Terms] OR "wnt"[All Fields] OR "Armadillo Domain Proteins"[Mesh] OR "armadillo"[All Fields]) AND ("mouth"[MeSH Terms] OR "mouth"[All Fields] OR "oral"[All Fields]) AND ("carcinoma, squamous cell"[MeSH Terms] OR ("carcinoma"[All Fields] AND "squamous"[All Fields] AND "cell"[All Fields]) OR "squamous cell carcinoma"[All Fields] OR "Neoplasms"[Mesh Terms] OR neopla*[All Fields] OR "cancer"[All Fields])	1145	October, 2021
Embase	('beta catenin'/exp OR 'β-catenin' OR 'beta-catenin' OR 'b-catenin' OR 'ctnnb gene'/exp OR 'CTNNB' OR 'canonical Wnt signaling'/exp OR 'wnt' OR 'armadillo domain protein'/exp OR 'armadillo') AND ('mouth'/exp OR 'mouth' OR 'oral') AND ('squamous cell carcinoma'/exp OR 'carcinoma' OR 'malignant neoplasm'/exp OR 'neoplas*' OR 'cancer')	2936	October, 2021
Web of Science	TS=("β-catenin" OR "beta-catenin" OR "b-catenin" OR "CTNNB" OR "wnt" OR "armadillo") AND TS=(mouth OR oral) AND TS=("squamous cell carcinoma" OR neoplas* or cancer)	1929	October, 2021
Scopus	TITLE-ABS-KEY(("β-catenin" OR "beta-catenin" OR "b-catenin" OR "CTNNB" OR "wnt" OR "armadillo") AND (mouth OR oral) AND ("squamous cell carcinoma" OR neoplas* or cancer))	1081	October, 2021
Total	7091		

*Truncation technique was used, shortening the word and adding a special character, to search for alternate forms and/or variant spellings of a term.

2. Characteristics of analyzed studies (*n* = 41)

Table S2. Characteristics of Analyzed Studies (*n* = 41; [1–41]).

Study	Year	Country	Publ Language	Study Design	Recruitment Period	Follow Up Mean ± SD (Range)	Sample Size, *n*	Sex M, *n* (%) F, *n* (%)	Age Mean ± SD (Range)	Tumour Subsites	Tobacco, *n* (%)	Alcohol, *n* (%)	RoB	Methods	Anti-β-catenin antibody (dilution, incubation time, temperature)	Subcellular location	IHQ cutoff point (%)	β-catenin + (%)
Bagutti et al. [1]	1998	UK	English	Retrospective cohort	NR	NR	22	M = 12 (54.54) F = 10 (45.46)	60.23 ± 14.24 (37–82)	Gingiva:12 Tongue:7 Oropharynx:2 FOM:1	NR	NR	high	IHQ	VB2 (NR, 1 h, NR)	Not defined	0	17 (77.27)
Lo Muzio et al. [2]	1999	Italy	English	Retrospective cohort	1991–1996	(12–96)	30	M=25 (83.33) F = 5 (16.67)	64.05 (27–81)	NR	NR	NR	high	IHQ	C19220 (1:700, 20 min, 24 °C)	Nuclear cytoplasmic	0	22 (73.33)
Gasparoni et al. [3]	2002	Brazil	English	Retrospective cohort	NR	NR	24	M = 19 (79.17) F = 5 (20.83)	61.5 ± 8.39 (47–78)	Tongue: 10 Rtm: 6 Fom: 5 Palate:3	23 (95.83)	22 (91.67)	moderate	IHQ	NR (1:1000, 90 min, room temperature)	Nuclear cytoplasmic	0	13 (54.17)
Miyashita et al. [4]	2003	Japan	English	Retrospective cohort	1992–2000	Median = 36 (5–112)	65	M = 41 (63.08) F = 24 (36.92)	64.0 (37–88)	NR	NR	NR	high	IHQ	NR (1:1000, overnight, 4 °C)	Nuclear-cytoplasmic	0	10 (15.38)
Tanaka et al. [5]	2003	Japan	English	Retrospective cohort	NR	(24–60)	159	M = 55 (33.96) F = 104 (63.04)	59.0 (26–85)	Tongue:84 Gingiva:33 Fom: 27 Bm:13 Lip:2	NR	NR	low	IHQ	Clone 14 (1:100, overnight, 4 °C)	Loss of membrane	75	45 (28.30)
Lim et al. [6]	2004	Japan	English	Retrospective cohort	1992–2000	(>24–NR)	56	M = 31 (55.36) F = 25 (44.64)	61 years (26–87)	tongue: 56	NR	NR	high	IHQ	Clone 14 (1:200, NR, NR)	Loss of membrane	50	18 (32.14)
Gao et al. [7]	2005	Taipei	English	Retrospective cohort	NR	NR	14	NR	NR	NR	NR	NR	high	IHQ	NR (NR)	Nuclear cytoplasmic	0	9 (64.29)
Odajima et al. [8]	2005	Japan	English	Retrospective cohort	NR	66 (5–134)	110	M = 80 (72.73) F = 30 (27.27)	59.0 (26–85)	Tongue:57 Fom:23 Gingiva:19 Bm:10 Lip:1	NR	NR	low	IHQ	C19220 (1:200,1 h, room temperature)	Nuclear-cytoplasmic	0	21 (19.09)
Zhang and Gao [9]	2005	China	Chinese	Retrospective cohort	1999–2004	NR	66	NR	NR	NR	NR	NR	high	IHQ	NR (NR)	Not defined	0	42 (63.64)
Iwai et al. [10]	2005	Japan	English	Retrospective cohort	NR	NR	20	NR	NR	NR	NR	NR	high	IHQ	NR (1:500, overnight, 4 °C)	Nuclear cytoplasmic	0	18 (90.00)
Fillies et al. [11]	2005	Germany	English	Retrospective cohort	NR	51.8 (36–120)	85	M = 71 (83.5) F = 14 (16.5)	57.0 (33–87)	Fom:85	NR	NR	high	IHQ	NR (1:1000, overnight, 4 °C)	Loss of membrane	15	71 (83.53)

Ueda et al. [12]	2006	Japan	English	Retrospective cohort	1990–1999	68<br>(49–166)	135	M = 94 (69.63)<br>F = 41 (30.37)	61.0<br>(23–89)	NR	NR	NR	low	IHQ	Clone 14<br>(1:250,<br>overnight,<br>4 °C)	Loss of membrane	90	57 (42.22)
Williams et al. [13]	2007	UK	English	Retrospective cohort	NR	(NR-60)	12	NR	NR	NR	NR	NR	high	IHQ	NR<br>(1:100,<br>NR,<br>NR)	Loss of membrane	0	8 (66.67)
de Aguiar et al. [14]	2007	Brazil	English	Retrospective cohort	1981–2000	NR<br>(24–120)	81	M = 70 (86.42)<br>F = 11 (13.58)	>56 = 40<br><56 = 41	Tongue:NR<br>Rtm:NR<br>Fom:NR<br>Gingiva:NR	64<br>(85.33)<br>Missing:6	55<br>(73.33)<br>Missing:6	high	IHQ	NR<br>(1:200,<br>overnight,<br>4 °C)	Loss of membrane	5	68 (83.95)
Wang et al. [15]	2007	Japan	English	Retrospective cohort	2001–2003	NR	48	M = 28 (58.33)<br>F = 20 (41.67)	59.0<br>(28–82)	Tongue:20<br>Gingiva:12<br>Fom:9<br>Bm:7	NR	NR	low	IHQ	E-5<br>(1:100,<br>overnight,<br>NR)	Loss of membrane	NR	16 (33.33)
Cai et al. [16]	2008	China	English	Retrospective cohort	NR	NR	76	M= 47 (61.84)<br>F= 29 (38.16)	62	Tongue:24<br>Fom:16<br>Gingiva:14<br>Bm:11<br>Lip:4<br>Palate:3<br>Other:4	NR	NR	high	IHQ	NR<br>(1:500,<br>overnight,<br>4 °C)	Loss of membrane	25	51 (67.11)
Xavier et al. [17]	2009	Brazil	English	Retrospective cohort	NR	NR	51	NR	NR	Lip:51	NR	NR	moderate	IHQ	Clone 14<br>(1:150,<br>30min,<br>room temperature)	Nuclear cytoplasmic	0	47 (91.16)
Liu et al. [18]	2010	China	English	Retrospective cohort	1994–2004	Median:51<br>(2–170)	83	M= 46 (55.52)<br>F= 37 (44.48)	58<br>(26–79)	Tongue:30<br>Bm:23<br>Gingiva:11<br>Palate:8<br>Lip:3<br>Fom:2<br>Rtm:2<br>Other:4	NR	NR	moderate	IHQ	Sc-7963<br>(NR,<br>60min,<br>room temperature)	Not defined	10	70 (84.34)
Lee et al. [19]	2010	Taiwan	English	Retrospective cohort	NR	NR	112	M = 93 (83.04)<br>F = 19 (16.96)	NR	NR	NR	NR	high	IHQ	NR<br>(NR,<br>overnight,<br>4 °C)	Nuclear cytoplasmic	0	26 (23.21)
De Almeidas et al. [20]	2010	Brazil	English	Retrospective cohort	NR	NR	24	NR	NR	Tongue:24	NR	NR	high	IHQ	Ab1<br>(1:500,<br>NR,<br>NR)	Nuclear cytoplasmic	10	10 (41.67)
Laxmidevi et al. [21]	2010	India	English	Retrospective cohort	NR	NR	30	NR	NR	NR	NR	NR	high	IHQ	NR<br>(1:50,<br>4h,<br>room temperature)	Nuclear cytoplasmic	0	5 (16.67)

Lee et al. [22]	2012	Taiwan	English	Retrospective cohort	NR	NR	40	M = 34 (85.0)<br>F = 6 (15.0)	Median = 54	NR	40 (100) areca quid chewers	NR	high	IHQ	NR<br>(1:50,<br>NR,<br>NR)	Nuclear cytoplasmic	0	23 (40)
Rosado et al. [23]	2012	Spain	English	Retrospective cohort	1990–1992	Median:53 (10–128)	69	M = 54 (78.26)<br>F = 15 (21.74)	Median = 61 (24–87)	Tongue: 29<br>Fom: 18<br>Gingiva: 8<br>Bm: 6<br>Palate: 4<br>Lip: 4	22 (31.88)	24 (34.78)	moderate	IHQ	FLEX Clone β-Catenin-1<br>(1:200,<br>20,<br>room temperature)	Loss of membrane	66	19 (27.54)
Kaur et al. [24]	2013	India	English	Retrospective cohort	2002–2005	Median 24 (NR-91)	105	NR	40 (29–75)	Bm:36<br>Tongue:35<br>Gingiva:12<br>Lip:6<br>Other:16	NR	NR	high	IHQ	NR<br>(NR,<br>120,<br>37 °C)	Loss of membrane	50	40 (38.10)
Kyridimou et al. [25]	2013	Greece	English	Retrospective cohort	NR	NR	25	NR	NR	Tongue (17)<br>Palate (4)<br>Gingiva (1) Bm (1)<br>Lip (1)	NR	NR	high	IHQ	NR<br>(NR,<br>NR,<br>NR)	Not defined	35	16 (64)
Ravindran et al. [26]	2014	India	English	Retrospective cohort	NR	31.9 (14–48)	60	M = 34 (56.67)<br>F = 26 (43.33)	59.3 (45–70)	Tongue (20)<br>Gingiva (14)<br>Fom (12)<br>Bm (9)<br>Palate (5)	38 (63.33)	34 (56.67)	moderate	IHQ	sc-7199<br>(1:200,<br>overnight,<br>4 °C)	Not defined	15	41 (68.33)
Zhang et al. [27]	2014	China	English	Retrospective cohort	2006–2008	NR (NR-60)	80	M = 52 (65.0)<br>F = 28 (35.0)	58.7 (36–77)	Tongue (27)<br>Gingiva (26)<br>Fom (15)<br>Bm (10)<br>Palate (2)	NR	NR	moderate	IHQ	NR<br>(NR,<br>1h,<br>room temperature)	Nuclear cytoplasmic	5	51 (63.75)
Balasundaram et al. [28]	2014	India	English	Retrospective cohort	2010–2012	NR	60	M = 44 (73.33)<br>F = 16 (26.67)	44.79 (23–72)	Tongue: 31<br>Bm: 29	NR	NR	high	IHQ	NR<br>(1:250,<br>overnight,<br>4 °C)	Not defined	10	44 (73.33)
Soares et al. [29]	2015	Brazil	English	Retrospective cohort	2001–2004	NR	40	M = 25 (62.5)<br>F = 15 (37.5)	57.8 (24–90)	Tongue:21<br>Fom:13<br>Other:6	33 (82.5)	28 (70.0)	high	IHQ	Clone-E5<br>(1:100,<br>NR,<br>NR)	Loss of membrane	50	12 (30)
Zhang et al. [30]	2015	China	English	Retrospective cohort	2007–2013	NR	109	M = 62 (56.88)<br>F = 47 (43.12)	56 (35–80)	Tongue:109	NR	NR	high	IHQ	Clone 610154<br>(1:200,<br>overnight,<br>4 °C)	Nuclear cytoplasmic	0	70
Reyes et al. [31]	2015	Chile	English	Retrospective cohort	NR	NR	36	M = 17 (47.22)<br>F = 19 (52.78)	NR	Tongue:13<br>Palate:2<br>Fom:3<br>Gingiva:4<br>Bm:9<br>Other:5	NR	NR	high	IHQ	NR<br>(1:200,<br>30,<br>37 °C)	Nuclear cytoplasmic	0	10 (27.78)

Zhou et al. [32]	2015	China	English	Retrospective cohort	2013–2014	NR	55	M = 52 (94.55)<br>F = 3 (5.45)	NR	Tongue:40<br>Others:12	NR	NR	high	IHQ	51067–2-AP<br>(1:100,<br>overnight,<br>4 °C)	Nuclear cytoplasmic	5	52 (94.55)
Pramanik et al. [33]	2016	India	English	Retrospective cohort	2013–2015	NR	112<br>Missing: 21	M = 69 (61.61)<br>F = 43 (38.39)	NR	Tongue:70<br>Bm:20<br>Lip:6<br>Gingiva:6<br>Others:10	NR	NR	high	IHQ	NR<br>(NR,<br>NR,<br>NR)	Nuclear cytoplasmic	0	56 (61.54)
Angadi et al. [34]	2016	India	English	Retrospective cohort	NR	NR	60	NR	NR	NR	NR	NR	moderate	IHQ	Clone E-247<br>(NR,<br>1h,<br>room temperature)	Nuclear cytoplasmic	0	51 (85.0)
Li et al. [35]	2018	China	English	Retrospective cohort	2014–2017	NR	38	M = 28 (73.68)<br>F = 10 (26.32)	61.92<br>(42–81)	NR	NR	NR	high	IHQ	NR<br>(1:100,<br>overnight,<br>4 °C)	Not defined	0	14 (36.84)
Ahmad et al. [36]	2019	Pakistan	English	Retrospective cohort	2017–2019	NR	70	NR	NR	NR	NR	NR	moderate	IHQ	Clone 17C2<br>(NR,<br>NR,<br>NR)	Nuclear cytoplasmic	0	62 (88.57)
Siriwardena et al. [37]	2020	Sri Lanka	English	Retrospective cohort	1999–2013	<60	290	M = 218 (75.17)<br>F = 72 (24.83)	NR<br>(31–85)	Bm:130<br>Tongue:73<br>Gingiva:26<br>Fom:23<br>Other:38	NR	NR	moderate	IHQ	M3539<br>(1:50,<br>1h,<br>room temperature)	Nuclear cytoplasmic	0	84 (28.97)
Sowmya et al. [38]	2020	India	English	Retrospective cohort	2014–2017	NR	40	NR	NR	NR	NR	NR	moderate	IHQ	Reporting bias<br>(1:50,<br>overnight,<br>4 °C)	Nuclear cytoplasmic	0	15 (37.5)
Kar et al. [39]	2021	India	English	Retrospective cohort	2012–2014	32.4<br>(NR-120)	80	M = 69 (86.25)<br>F = 11 (13.75)	NR	Gingiva: 26<br>Tongue: 24<br>Bm: 23<br>Rtm: 6<br>Fom: 1	NR	NR	moderate	IHQ	Ab6302<br>(1:500,<br>NR,<br>NR)	Not defined	0	47 (58.75)
Kumar et al. [40]	2021	India	English	Retrospective cohort	NR	NR	30	NR	NR	NR	NR	NR	high	IHQ	NR<br>(NR,<br>NR,<br>NR)	Not defined	10	15 (50.0)
Al-Rawi et al. [41]	2021	Saudi Arabia	English	Retrospective cohort	2010–2019	NR<br>(6–72)	65	M = 46 (70.77)<br>F = 19 (29.23)	Median:57<br>(18–78)	Tongue: 41<br>Bm: 9<br>Gingiva: 6<br>Others:9	16<br>(24.81)	NR	low	IHQ	Clone 14<br>(1:150,<br>NR,<br>NR)	Nuclear cytoplasmic	NR	NR

Abbreviations**:** Bm, buccal mucosa; fom, floor of mouth; IHQ, immunohistochemistry; OSCC, oral squamous cell carcinoma; m, months; n, number; NR, not reported; RoB, risk of bias; SD, standard deviation; y, years.

References for Table S2

1. Bagutti, C.; Speight, P.M.; Watt, F.M. Comparison of integrin, cadherin, and catenin expression in squamous cell carcinomas of the oral cavity. *J. Pathol.* **1998**, *186*, 8–16, https://doi.org/10.1002/(SICI)1096-9896(199809)186:1<8::AID-PATH156>3.0.CO;2-H.
2. Muzio, L. Lo; Staibano, S.; Pannone, G.; Grieco, M.; Mignogna, M.D.; Cerrato, A.; Testa, N.F.; De Rosa, G. Beta- and gamma-catenin expression in oral squamous cell carcinomas. *Anticancer Res.* **1999**, *19*, 3817–3826.
3. Gasparoni, A.; Chaves, A.; Fonzi, L.; Johnson, G.K.; Schneider, G.B.; Squier, C.A. Subcellular localization of beta-catenin in malignant cell lines and squamous cell carcinomas of the oral cavity. *J. Oral Pathol. Med.* **2002**, *31*, 385–394, https://doi.org/10.1034/j.1600-0714.2002.00108.x.
4. Miyashita, H.; Mori, S.; Motegi, K.; Fukumoto, M.; Uchida, T. Pin1 is overexpressed in oral squamous cell carcinoma and its levels correlate with cyclin D1 overexpression. *Oncol. Rep.* **2003**, *10*, 455–461, https://doi.org/10.3892/or.10.2.455.
5. Tanaka, N.; Odajima, T.; Ogi, K.; Ikeda, T.; Satoh, M. Expression of E-cadherin, α-catenin, and β-catenin in the process of lymph node metastasis in oral squamous cell carcinoma. *Br. J. Cancer* **2003**, *89*, 557–563, https://doi.org/10.1038/sj.bjc.6601124.
6. Lim, S.C.; Zhang, S.; Ishii, G.; Endoh, Y.; Kodama, K.; Miyamoto, S.; Hayashi, R.; Ebihara, S.; Cho, J.S.; Ochiai, A. Predictive Markers for Late Cervical Metastasis in Stage I and II Invasive Squamous Cell Carcinoma of the Oral Tongue. *Clin. Cancer Res.* **2004**, *10*, 166–172, https://doi.org/10.1158/1078-0432.CCR-0533-3.
7. Gao, S.; Eiberg, H.; Krogdahl, A.; Liu, C.J.; Sørensen, J.A. Cytoplasmic expression of E-cadherin and β-catenin correlated with LOH and hypermethylation of the APC gene in oral squamous cell carcinomas. *J. Oral Pathol. Med.* **2005**, *34*, 116–119, https://doi.org/10.1111/j.1600-0714.2004.00275.x.
8. Odajima, T.; Sasaki, Y.; Tanaka, N.; Kato-Mori, Y.; Asanuma, H.; Ikeda, T.; Satoh, M.; Hiratsuka, H.; Tokino, T.; Sawada, N. Abnormal β-catenin expression in oral cancer with no gene mutation: Correlation with expression of cyclin D1 and epidermal growth factor receptor, Ki-67 labeling index, and clinicopathological features. *Hum. Pathol.* **2005**, *36*, 234–241, https://doi.org/10.1016/j.humpath.2004.12.009.
9. Zhang, W.; Gao, Y. [Roles of Wnt-1, beta-catenin and adenomatous polyposis coli in the differentiation and proliferation of oral squamous cell carcinoma]. *Zhonghua Kou Qiang Yi Xue Za Zhi* **2005**, *40*, 491–494.
10. Iwai, S.; Katagiri, W.; Kong, C.; Amekawa, S.; Nakazawa, M.; Yura, Y. Mutations of the APC, beta-catenin, and axin 1 genes and cytoplasmic accumulation of beta-catenin in oral squamous cell carcinoma. *J. Cancer Res. Clin. Oncol.* **2005**, *131*, 773–782, https://doi.org/10.1007/s00432-005-0027-y.
11. Fillies, T.; Buerger, H.; Gaertner, C.; August, C.; Brandt, B.; Joos, U.; Werkmeister, R. Catenin expression in T1/2 carcinomas of the floor of the mouth. *Int. J. Oral Maxillofac. Surg.* **2005**, *34*, 907–911, https://doi.org/10.1016/j.ijom.2005.03.010.
12. Ueda, G.; Sunakawa, H.; Nakamori, K.; Shinya, T.; Tsuhako, W.; Tamura, Y.; Kosugi, T.; Sato, N.; Ogi, K.; Hiratsuka, H. Aberrant expression of β- and γ-catenin is an independent prognostic marker in oral squamous cell carcinoma. *Int. J. Oral Maxillofac. Surg.* **2006**, *35*, 356–361, https://doi.org/10.1016/j.ijom.2005.07.023.
13. Williams, H.K.; Sanders, D.S.A.; Jankowski, J.A.Z.; Landini, G.; Brown, A.M.S. Expression of cadherins and catenins in oral epithelial dysplasia and squamous cell carcinoma. *J. Oral Pathol. Med.* **1998**, *27*, 308–317, https://doi.org/10.1111/j.1600-0714.1998.tb01962.x.
14. de Aguiar, F.C.A.; Kowalski, L.P.; de Almeida, O.P. Clinicopathological and immunohistochemical evaluation of oral squamous cell carcinoma in patients with early local recurrence. *Oral Oncol.* **2007**, *43*, 593–601, https://doi.org/10.1016/j.oraloncology.2006.07.003.
15. Wang, L.; Liu, T.; Wang, Y.; Cao, L.; Nishioka, M.; Aguirre, R.L.; Ishikawa, A.; Geng, L.; Okada, N. Altered expression of desmocollin 3, desmoglein 3, and β-catenin in oral squamous cell

carcinoma: Correlation with lymph node metastasis and cell proliferation. *Virchows Arch.* **2007**, *451*, 959–966, https://doi.org/10.1007/s00428-007-0485-5.
16. Cai, Z.G.; Shi, X.J.; Gao, Y.; Wei, M.J.; Wang, C.Y.; Yu, G.Y. Β-Catenin Expression Pattern in Primary Oral Squamous Cell Carcinoma. *Chin. Med. J. (Engl).* **2008**, *121*, 1866–1870, https://doi.org/10.1097/00029330-200810010-00003.
17. Xavier, F.C.A.; Rodini, C.O.; Ramalho, L.M.P.; Mantesso, A.; Nunes, F.D. WNT-5A, but not matrix metalloproteinase 3 or β-catenin protein, expression is related to early stages of lip carcinogenesis. *J. Oral Pathol. Med.* **2009**, *38*, 708–715, https://doi.org/10.1111/j.1600-0714.2009.00756.x.
18. Liu, L.K.; Jiang, X.Y.; Zhou, X.X.; Wang, D.M.; Song, X.L.; Jiang, H.B. Upregulation of vimentin and aberrant expression of E-cadherin/Β- catenin complex in oral squamous cell carcinomas: Correlation with the clinicopathological features and patient outcome. *Mod. Pathol.* **2010**, *23*, 213–224, https://doi.org/10.1038/modpathol.2009.160.
19. Lee, C.H.; Hung, H.W.; Hung, P.H.; Shieh, Y.S. Epidermal growth factor receptor regulates β-catenin location, stability, and transcriptional activity in oral cancer. *Mol. Cancer* **2010**, *9*, 64. https://doi.org/10.1186/1476-4598-9-64.
20. De Almeidas, F.; Silveira, E.J.D.; Da Silveira, J.P.B.; Da Silva, F.M.; De Amorim, R.F.B. Correlation of β-catenin expresssion and metastasis in tongue squamous cell carcinoma. *Acta Cir. Bras.* **2010**, *25*, 513–517, https://doi.org/10.1590/S0102-86502010000600010.
21. Laxmidevi, L.B.; Angadi, P. V.; Pillai, R.K.; Chandreshekar, C. Aberrant β-catenin expression in the histologic differentiation of oral squamous cell carcinoma and verrucous carcinoma: an immunohistochemical study. *J. Oral Sci.* **2010**, *52*, 633–640, https://doi.org/10.2334/josnusd.52.633.
22. Lee, S.S.; Tsai, C.H.; Tsai, L.L.; Chou, M.C.; Chou, M.Y.; Chang, Y.C. Β-Catenin Expression in Areca Quid Chewing-Associated Oral Squamous Cell Carcinomas and Upregulated By Arecoline in Human Oral Epithelial Cells. *J. Formos. Med. Assoc.* **2012**, *111*, 194–200, https://doi.org/10.1016/j.jfma.2010.11.002.
23. Rosado, P.; Lequerica-Fernández, P.; Fernández, S.; Allonca, E.; Villallaín, L.; De Vicente, J.C. E-cadherin and β-catenin expression in well-differentiated and moderately-differentiated oral squamous cell carcinoma: Relations with clinical variables. *Br. J. Oral Maxillofac. Surg.* **2013**, *51*, 149–156, https://doi.org/10.1016/j.bjoms.2012.03.018.
24. Kaur, J.; Sawhney, M.; DattaGupta, S.; Shukla, N.K.; Srivastava, A.; Walfish, P.G.; Ralhan, R. Clinical Significance of Altered Expression of β-Catenin and E-Cadherin in Oral Dysplasia and Cancer: Potential Link with ALCAM Expression. *PLoS One* **2013**, *8*, e67361. https://doi.org/10.1371/journal.pone.0067361.
25. Kyrodimou, M.; Andreadis, D.; Drougou, A.; Amanatiadou, E.; Angelis, L.; Barbatis, C.; Epivatianos, A.; Vizirianakis, I. Desmoglein-3/γ-catenin and E-cadherin/β-catenin differential expression in oral leukoplakia and squamous cell carcinoma. *Clin. Oral Investig.* **2014**, *18*, 199–210.
26. Ravindran, G.; Sawant, S.S.; Hague, A.; Kingsley, K.; Devaraj, H. Association of differential β-catenin expression with Oct-4 and Nanog in oral squamous cell carcinoma and their correlation with clinicopathological factors and prognosis. *Head Neck* **2015**, *37*, 982–993, https://doi.org/10.1002/hed.23699.
27. Zhang, S.; Zhou, X.; Wang, B.; Zhang, K.; Liu, S.; Yue, K.; Zhang, L.; Wang, X. Loss of VHL expression contributes to epithelial-mesenchymal transition in oral squamous cell carcinoma. *Oral Oncol.* **2014**, *50*, 809–817, https://doi.org/10.1016/j.oraloncology.2014.06.007.
28. Balasundaram, P.; Singh, M.K.; Dinda, A.K.; Thakar, A.; Yadav, R. Study of β-catenin, E-cadherin and vimentin in oral squamous cell carcinoma with and without lymph node metastases. *Diagn. Pathol.* **2014**, *9*, 145. https://doi.org/10.1186/1746-1596-9-145.
29. Soares, M.Q.S.; Mendonça, J.A.; Morais, M.O.; Leles, C.R.; Batista, A.C.; Mendonça, E.F. E-cadherin, β-catenin, and α2β1 and α3β1 integrin expression in primary oral squamous cell carcinoma and its regional metastasis. *Histol. Histopathol.* **2015**, *30*, 1213–22, https://doi.org/10.14670/HH-11-616.

30. Zhang, P.; Cao, H.Y.; Bai, L.L.; Li, W.N.; Wang, Y.; Chen, S.Y.; Zhang, L.; Yang, L.H.; Xu, H.T.; Wang, E.H. The high expression of TC1 (C8orf4) was correlated with the expression of β-catenin and cyclin D1 and the progression of squamous cell carcinomas of the tongue. *Tumor Biol.* **2015**, *36*, 7061–7067, https://doi.org/10.1007/s13277-015-3423-1.
31. Reyes, M.; Rojas-Alcayaga, G.; Maturana, A.; Aitken, J.P.; Rojas, C.; Ortega, A.V. Increased nuclear β-catenin expression in oral potentially malignant lesions: A marker of epithelial dysplasia. *Med. Oral Patol. Oral Cir. Bucal* **2015**, *20*, e540–e546, https://doi.org/10.4317/medoral.20341.
32. Zhou, S.; Chen, L.; Mashrah, M.; Zhu, Y.; Liu, J.; Yang, X.; He, Z.; Wang, L.; Xiang, T.; Yao, Z.; et al. Deregulation of secreted frizzled-related proteins is associated with aberrant β-catenin activation in the carcinogenesis of oral submucous fibrosis. *Onco. Targets. Ther.* **2015**, *8*, 2923–2931, https://doi.org/10.2147/ott.s91460.
33. Angadi, P. V.; Patil, P. V.; Angadi, V.; Mane, D.; Shekar, S.; Hallikerimath, S.; Kale, A.D.; Kardesai, S.G. Immunoexpression of Epithelial Mesenchymal Transition Proteins E-Cadherin, β-Catenin, and N-Cadherin in Oral Squamous Cell Carcinoma. *Int. J. Surg. Pathol.* **2016**, *24*, 696–703, https://doi.org/10.1177/1066896916654763.
34. Pramanik, K.K.; Singh, A.K.; Alam, M.; Kashyap, T.; Mishra, P.; Panda, A.K.; Dey, R.K.; Rana, A.; Nagini, S.; Mishra, R. Reversion-inducing cysteine-rich protein with Kazal motifs and its regulation by glycogen synthase kinase 3 signaling in oral cancer. *Tumor Biol.* **2016**, *37*, 15253–15264, https://doi.org/10.1007/s13277-016-5362-x.
35. Li, Y.; Xu, Z.; Li, J.; Ban, S.; Duan, C.; Liu, W. Interleukin-18 expression in oral squamous cell carcinoma: its role in tumor cell migration and invasion, and growth of tumor cell xenografts. *FEBS Open Bio* **2018**, *8*, 1953–1963, https://doi.org/10.1002/2211-5463.12532.
36. Ahmad, B.; Asif, M.; Ali, A.; Jamal, S.; Khan, M.Z.; Khadim, M.T. Expression of Ki-67 and beta-catenin in pseudoepitheliomatous hyperplasia and squamous cell carcinoma in oral mucosal biopsies: An immunohistochemical study. *Asian Pacific J. Cancer Prev.* **2020**, *21*, 157–161, https://doi.org/10.31557/APJCP.2020.21.1.157.
37. Siriwardena, B.S.M.S.; Karunathilaka, H.D.N.U.; Kumarasiri, P.V.R.; Tilakaratne, W.M. Impact of Histological and Molecular Parameters on Prognosis of Oral Squamous Cell Carcinoma: Analysis of 290 Cases. *Biomed Res. Int.* **2020**, 2059240. https://doi.org/10.1155/2020/2059240.
38. Sowmya, S.; Rao, R.; Prasad, K. Prediction of metastasis in oral squamous cell carcinoma through phenotypic evaluation and gene expression of E-cadherin, β-catenin, matrix metalloproteinase-2, and matrix metalloproteinase-9 biomarkers with clinical correlation. *J. Carcinog.* **2020**, *19*, 8, https://doi.org/10.4103/jcar.jcar_8_20.
39. Kar, M.; Sultania, M.; Roy, S.; Padhi, S.; Banerjee, B. β-Catenin—a Possible Prognostic Molecular Marker for Recurrence in Histopathologically Negative Surgical Margin of Oral Cancer. *Indian J. Surg. Oncol.* **2021**, *12*, 128–133, https://doi.org/10.1007/s13193-020-01217-0.
40. Kumar, V.; Panda, A.; Dash, K.; Bhuyan, L.; Mahapatra, N.; Mishra, P. Immunohistochemical Expression of the Epithelial to Mesenchymal Transition Proteins E-cadherin and β-catenin in Grades of Oral Squamous Cell Carcinoma. *J. Pharm. Bioallied Sci.* **2021**, *13*, S555–S560, https://doi.org/10.4103/jpbs.JPBS_562_20.
41. Al-Rawi, N.; Al Ani, M.; Quadri, A.; Hamdoon, Z.; Awwad, A.; Al Kawas, S.; Al Nuaimi, A. Prognostic Significance of E-Cadherin, B-Catenin and Cyclin D1 in Oral Squamous Cell Carcinoma: a tissue microarray study. *Histol. Histopathol.* **2021**, 36, 18363, https://doi.org/10.14670/HH-18-363.

3. Meta-Analysis on the Aberrant β-Catenin Expression and Overall Survival in OSCC

3.1. Subgroup Meta-Analysis by Geographical Area

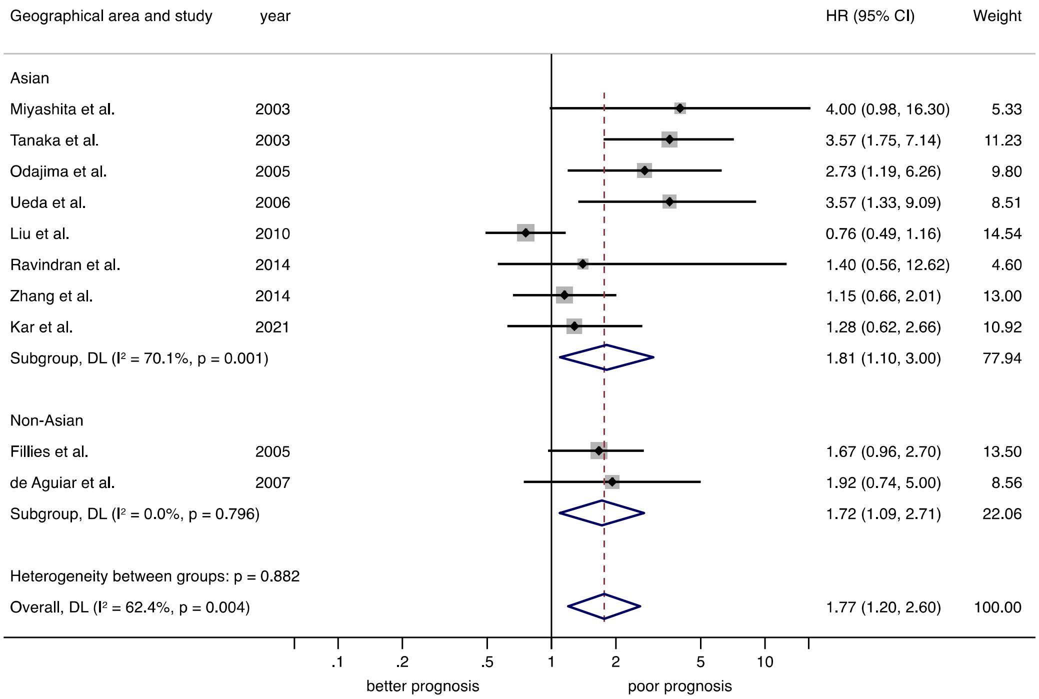

Figure S1. Forest plot graphically representing the stratified analysis by geographical area on the association between the aberrant β-catenin expression and overall survival in patients with OSCC. OSCC, oral squamous cell carcinoma; HR, hazard ratio; CI, confidence intervals. Random-effects model, inverse-variance weighting (based on the DerSimonian and Laird method). A HR > 1 suggests that the aberrant β-catenin expression is associated with poor overall survival. Diamonds indicate the pooled HRs with their corresponding 95% CIs. The forest plot was constructed using Stata software (v.16.1, Stata Corp, College Station, TX, USA).

3.2. *Subgroup Meta-Analysis by Anti-β-Catenin Antibody*

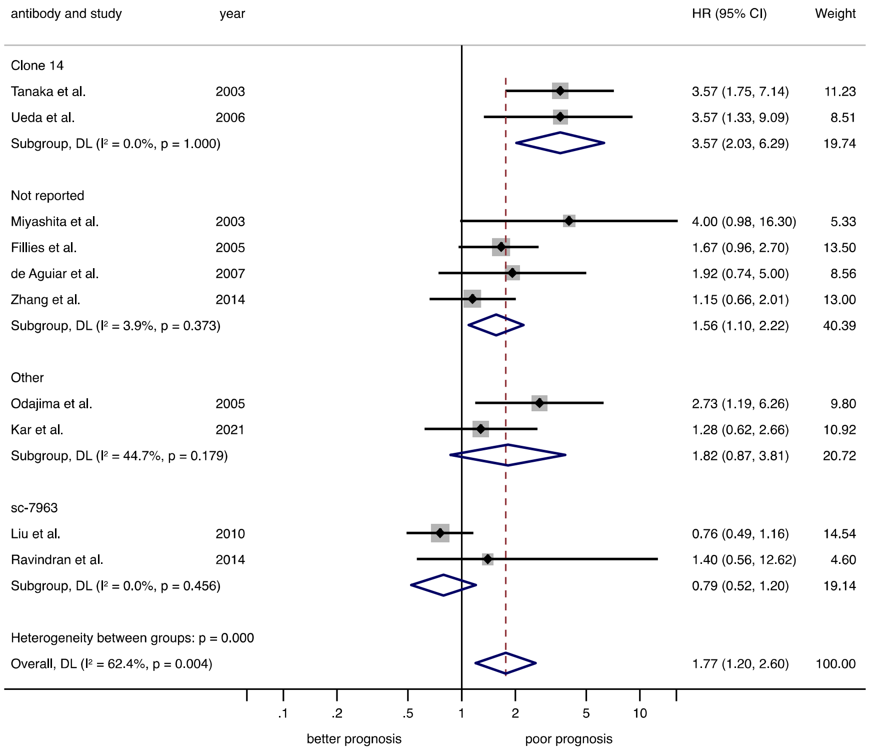

Figure S2. Forest plot graphically representing the stratified analysis by anti-β-catenin antibody on the association between the aberrant β-catenin expression and overall survival in patients with OSCC. OSCC, oral squamous cell carcinoma; HR, hazard ratio; CI, confidence intervals. Random-effects model, inverse-variance weighting (based on the DerSimonian and Laird method). A HR > 1 suggests that the aberrant β-catenin expression is associated with poor overall survival. Diamonds indicate the pooled HRs with their corresponding 95% CIs. The forest plot was constructed using Stata software (v.16.1, Stata Corp, College Station, TX, USA).

3.3. Subgroup Meta-Analysis by Anti-β-Catenin Antibody Dilution

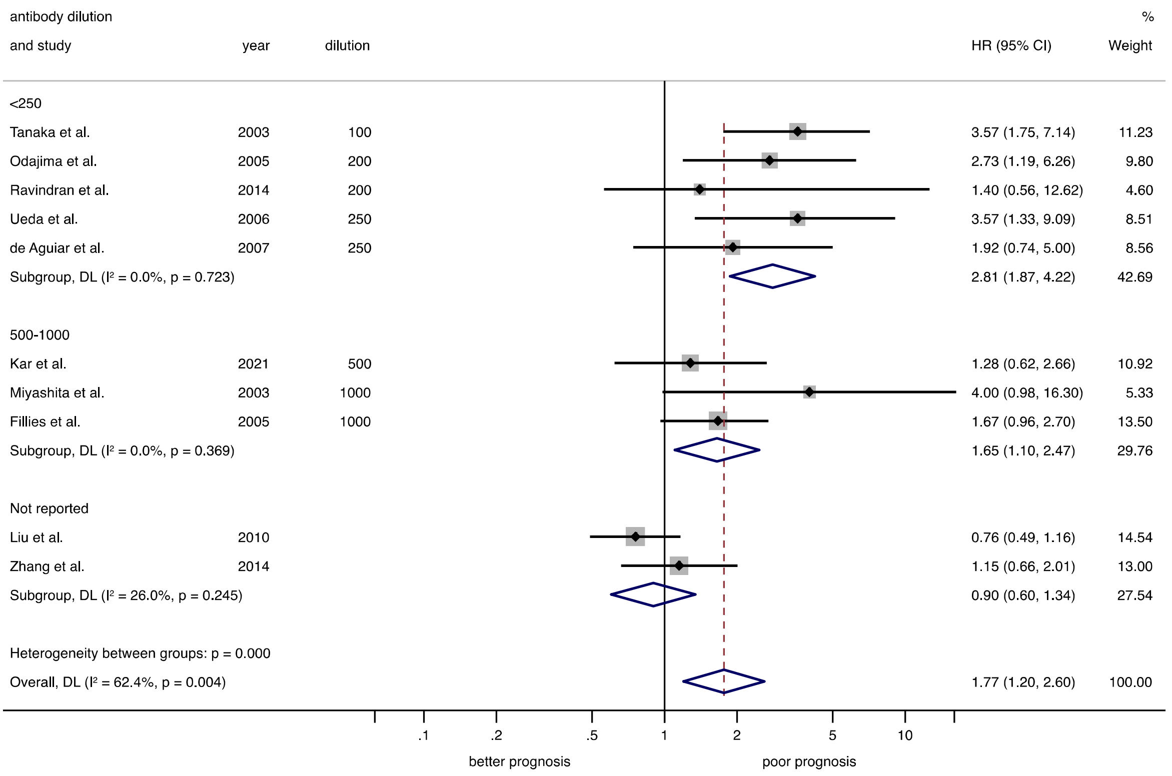

Figure S3. Forest plot graphically representing the stratified analysis by anti-β-catenin antibody dilution on the association between the aberrant β-catenin expression and overall survival in patients with OSCC. OSCC, oral squamous cell carcinoma; HR, hazard ratio; CI, confidence intervals. Random-effects model, inverse-variance weighting (based on the DerSimonian and Laird method). A HR > 1 suggests that the aberrant β-catenin expression is associated with poor overall survival. Diamonds indicate the pooled HRs with their corresponding 95% CIs. The forest plot was constructed using Stata software (v.16.1, Stata Corp, College Station, TX, USA).

3.4. Subgroup Meta-Analysis by Anti-β-Catenin Antibody Incubation Time

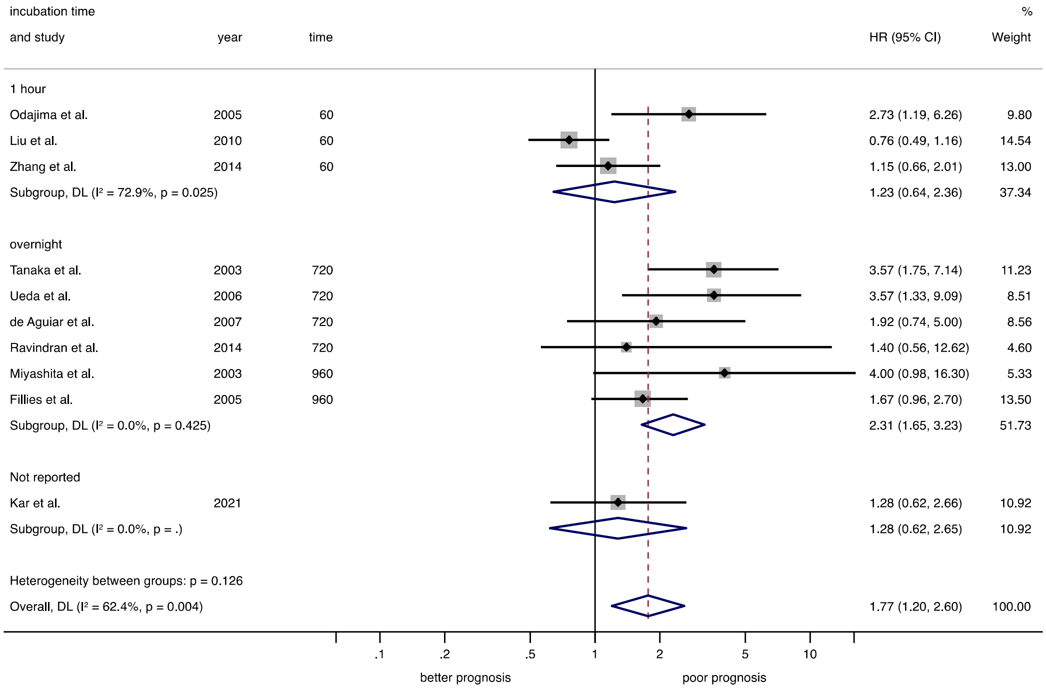

Figure S4. Forest plot graphically representing the stratified analysis by anti-β-catenin antibody incubation time on the association between the aberrant β-catenin expression and overall survival in patients with OSCC. OSCC, oral squamous cell carcinoma; HR, hazard ratio; CI, confidence intervals. Random-effects model, inverse-variance weighting (based on the DerSimonian and Laird method). A HR > 1 suggests that the aberrant β-catenin expression is associated with poor overall survival. Diamonds indicate the pooled HRs with their corresponding 95% CIs. The forest plot was constructed using Stata software (v.16.1, Stata Corp, College Station, TX, USA).

3.5. Subgroup Meta-Analysis by Anti-β-Catenin Antibody Incubation Temperature

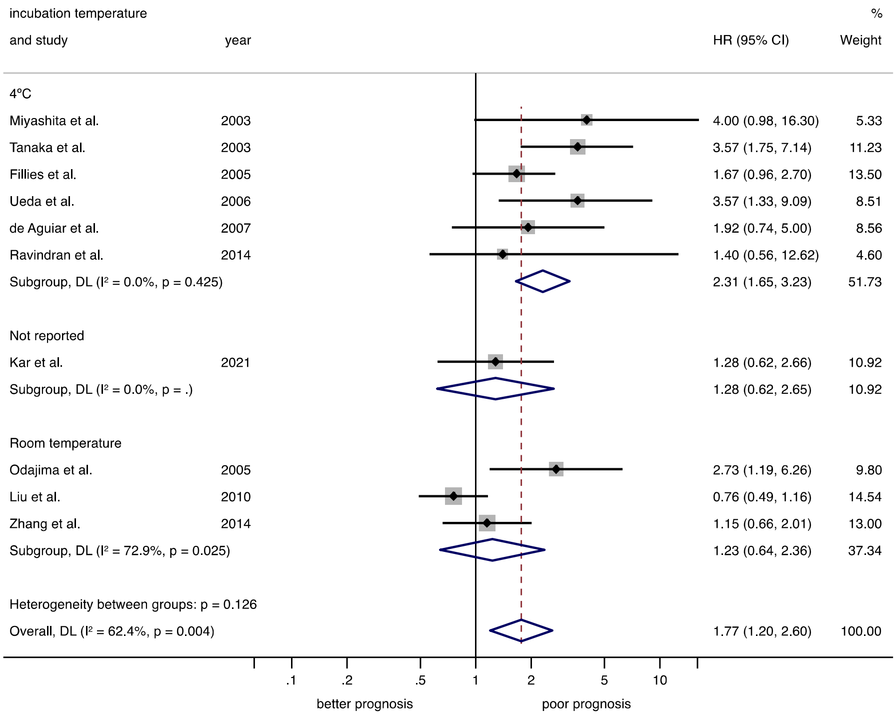

Figure S5. Forest plot graphically representing the stratified analysis by anti-β-catenin antibody incubation temperature on the association between the aberrant β-catenin expression and overall survival in patients with OSCC. OSCC, oral squamous cell carcinoma; HR, hazard ratio; CI, confidence intervals. Random-effects model, inverse-variance weighting (based on the DerSimonian and Laird method). A HR > 1 suggests that the aberrant β-catenin expression is associated with poor overall survival. Diamonds indicate the pooled HRs with their corresponding 95% CIs. The forest plot was constructed using Stata software (v.16.1, Stata Corp, College Station, TX, USA).

3.6. Subgroup Meta-Analysis by Overall Risk of Bias in Primary-Level Studies

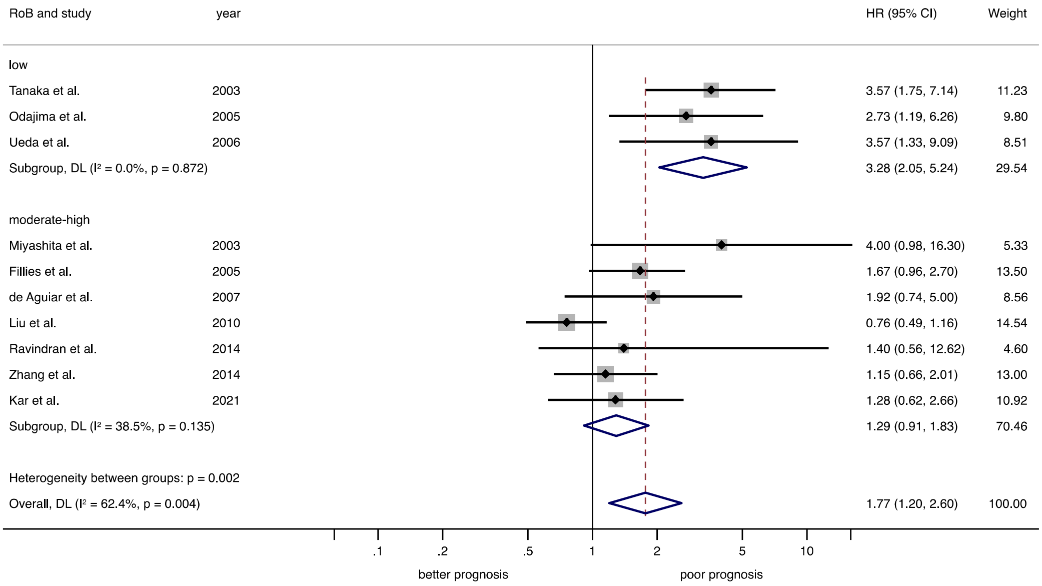

Figure S6. Forest plot graphically representing the stratified analysis by overall RoB in primary-level studies, on the association between the aberrant β-catenin expression and overall survival in patients with OSCC. OSCC, oral squamous cell carcinoma; RoB, risk of bias; HR, hazard ratio; CI, confidence intervals. Random-effects model, inverse-variance weighting (based on the DerSimonian and Laird method). A HR > 1 suggests that the aberrant β-catenin expression is associated with poor overall survival. Diamonds indicate the pooled HRs with their corresponding 95% CIs. The forest plot was constructed using Stata software (v.16.1, Stata Corp, College Station, TX, USA).

3.7. Univariable Meta-Regression on the Effect of Follow Up

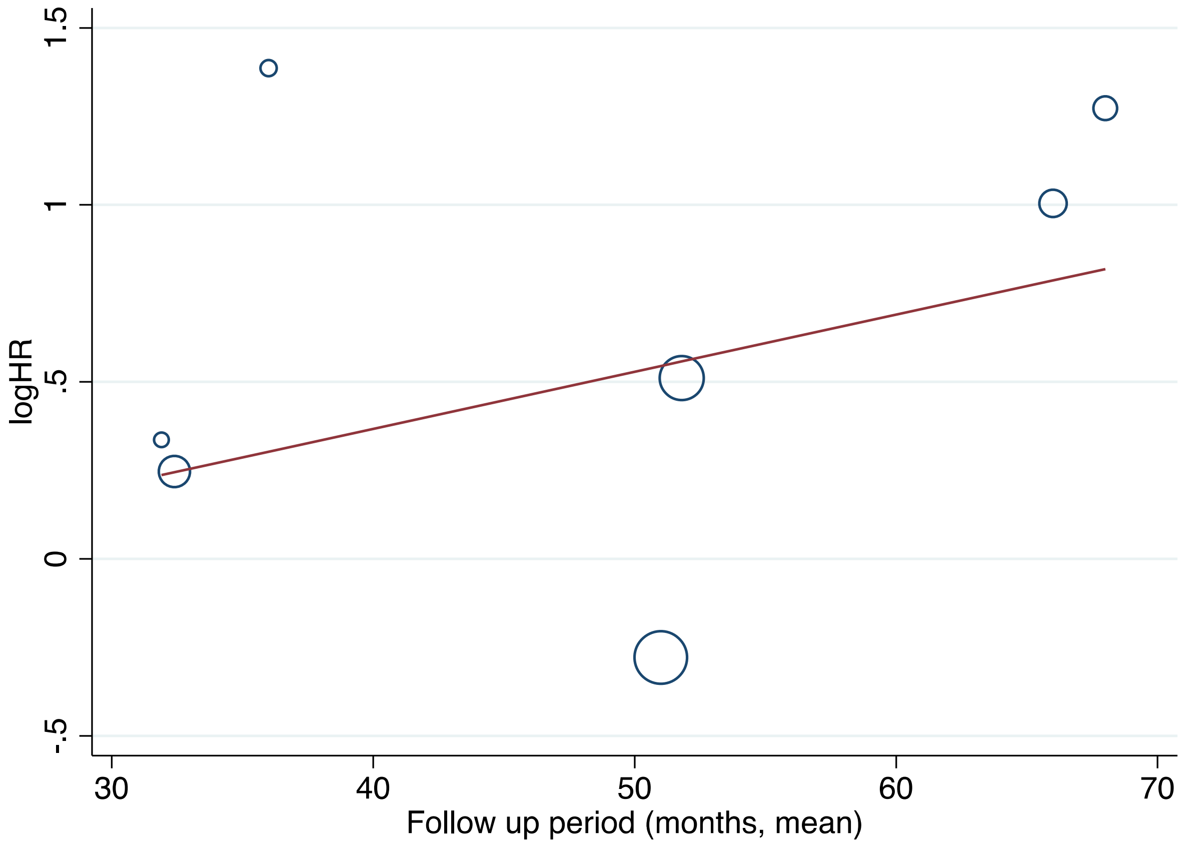

Figure S7. Bubble plot graphically representing the univariable meta-regression analysis of the potential effect of follow up period (expressed in months, in x−axis) on the association between the aberrant β-catenin expression and overall survival in patients with OSCC (using HR as effect size measure, in y−axis). OSCC, oral squamous cell carcinoma; HR, hazard ratio; log, natural logarithm (i.e., log base e). The red line exhibits the fitted regression line together with blue circles representing the estimates from each individual study, sized according to the precision of each estimate (the inverse of its within-study variance). The bubble plot was constructed using Stata software (v.16.1, Stata Corp, College Station, TX, USA).

3.8. Univariable Meta-Regression on the Effect of Sex

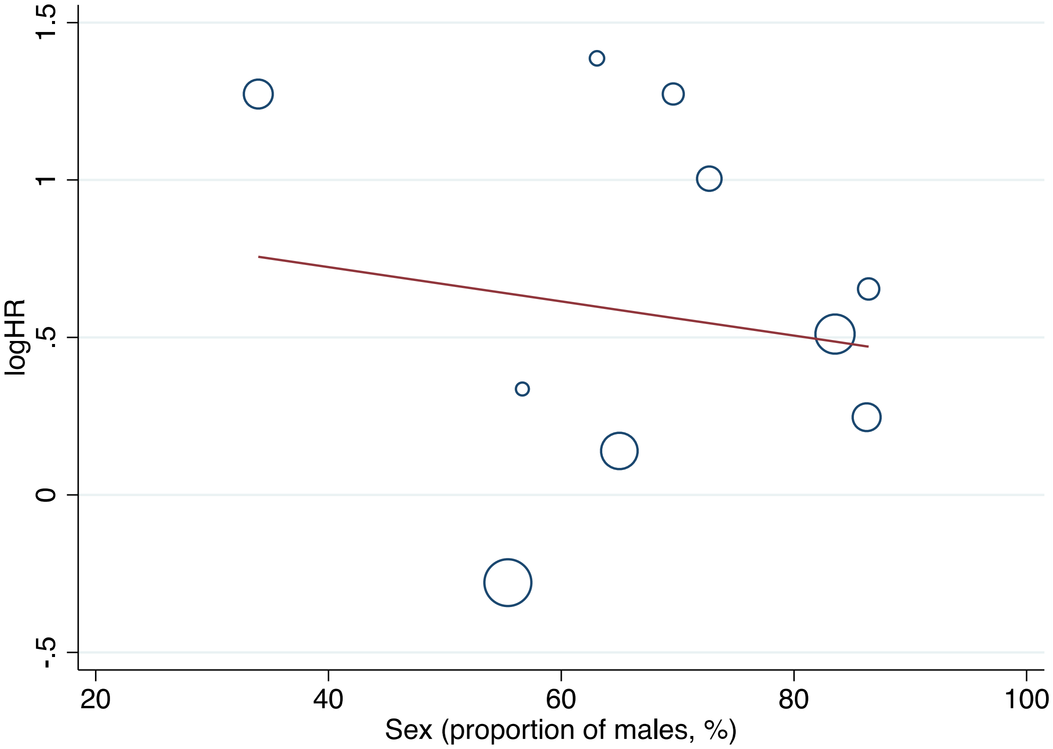

Figure S8. Bubble plot graphically representing the univariable meta-regression analysis of the potential effect of sex (% of males, in x−axis) on the association between the aberrant β-catenin expression and overall survival in patients with OSCC (using HR as effect size measure, in y−axis). OSCC, oral squamous cell carcinoma; HR, hazard ratio; log, natural logarithm (i.e., log base e). The red line exhibits the fitted regression line together with blue circles representing the estimates from each individual study, sized according to the precision of each estimate (the inverse of its within-study variance). The bubble plot was constructed using Stata software (v.16.1, Stata Corp, College Station, TX, USA).

3.9. Univariable Meta-Regression on the Effect of Age

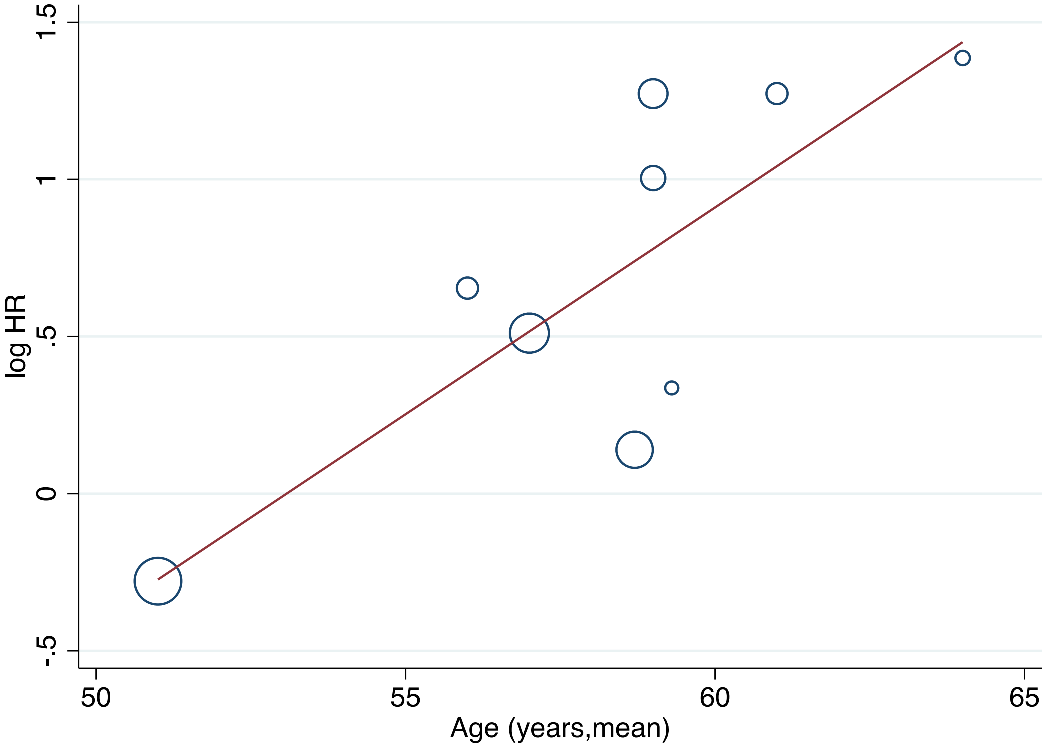

Figure S9. Bubble plot graphically representing the univariable meta-regression analysis of the potential effect of age (mean age of patients, expressed in years, in x–axis) on the association between the aberrant β-catenin expression and overall survival in patients with OSCC (using HR as effect size measure, in y–axis). OSCC, oral squamous cell carcinoma; HR, hazard ratio; log, natural logarithm (i.e., log base e). The red line exhibits the fitted regression line together with blue circles representing the estimates from each individual study, sized according to the precision of each estimate (the inverse of its within-study variance). The bubble plot was constructed using Stata software (v.16.1, Stata Corp, College Station, TX, USA).

3.10. Univariable Meta-Regression on the Effect of Clinical Stage

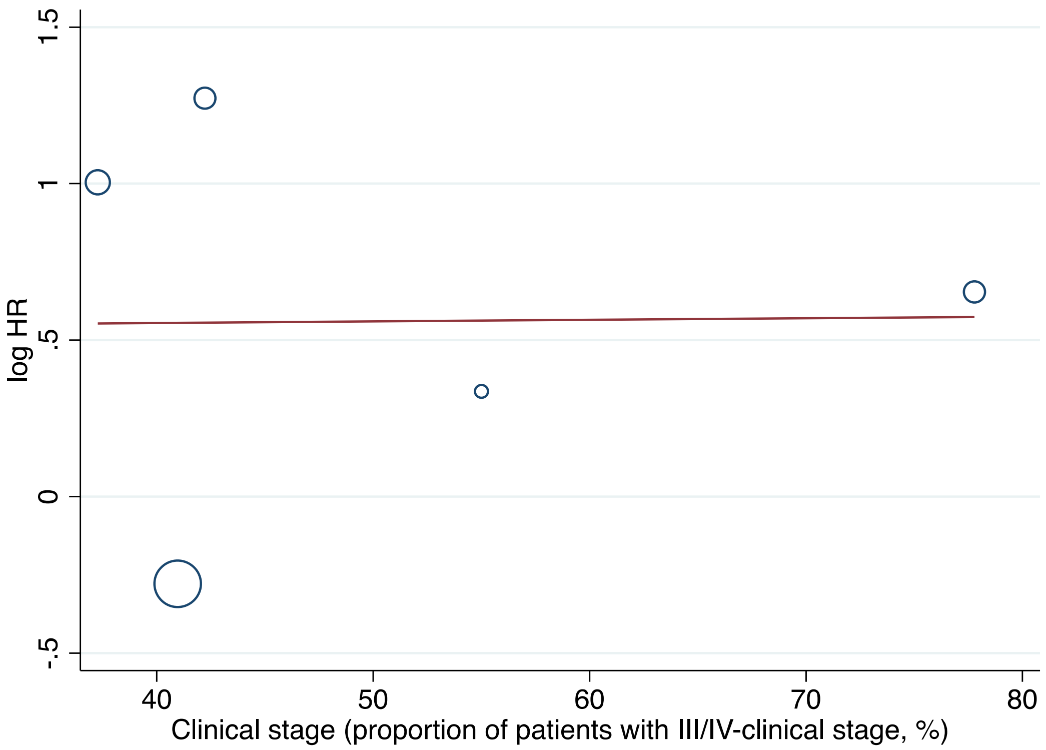

Figure S10. Bubble plot graphically representing the univariable meta-regression analysis of the potential effect of clinical stage (% of stage III/IV patients, in x–axis) on the association between the aberrant β-catenin expression and overall survival in patients with OSCC (using HR as effect size measure, in y–axis). OSCC, oral squamous cell carcinoma; HR, hazard ratio; log, natural logarithm (i.e., log base e). The red line exhibits the fitted regression line together with blue circles representing the estimates from each individual study, sized according to the precision of each estimate (the inverse of its within-study variance). The bubble plot was constructed using Stata software (v.16.1, Stata Corp, College Station, TX, USA).

4. Meta-Analysis on the Aberrant β-Catenin Expression and DFS in OSCC

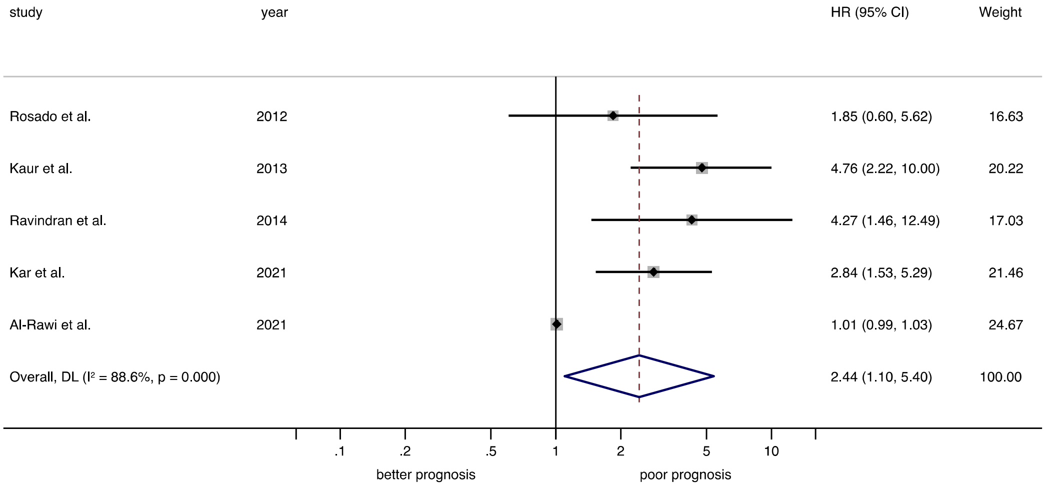

Figure S11. Forest plot graphically representing the stratified analysis on the association between the aberrant β-catenin expression and DFS in patients with OSCC. DFS, disease-free survival; OSCC, oral squamous cell carcinoma; HR, hazard ratio; CI, confidence intervals. Random-effects model, inverse-variance weighting (based on the DerSimonian and Laird method). A HR > 1 suggests that the aberrant β-catenin expression is associated with poor DFS. Diamonds indicate the pooled HRs with their corresponding 95% CIs. The forest plot was constructed using Stata software (v.16.1, Stata Corp, College Station, TX, USA).

5. Meta-analysis on the aberrant β-catenin expression and T status in OSCC

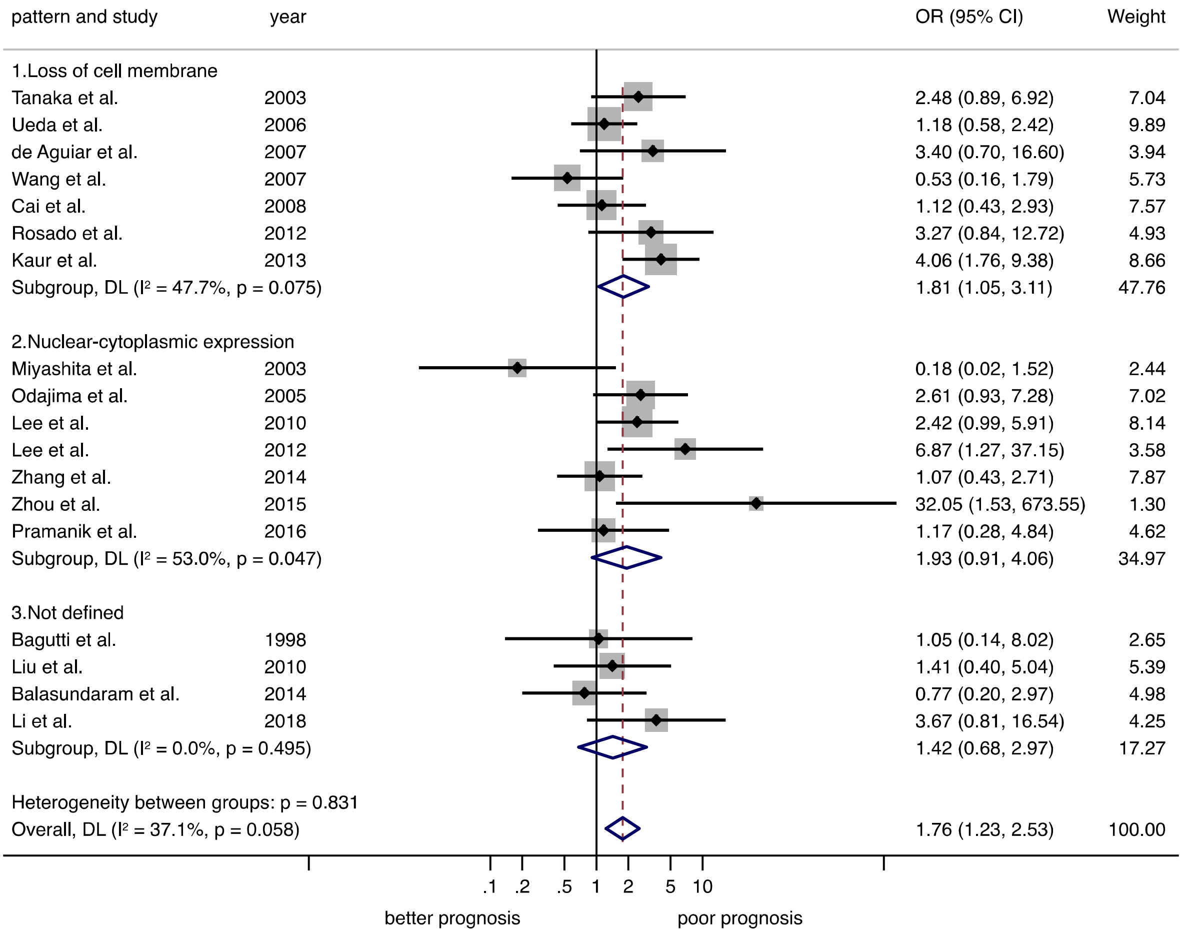

Figure S12. Forest plot graphically representing the stratified analysis by anti-β-catenin subcellular location on the association between the aberrant β-catenin expression and T status (T3/T4 vs. T1/T2) in patients with OSCC. OSCC, oral squamous cell carcinoma; OR, odds ratio; CI, confidence intervals. Random-effects model, inverse-variance weighting (based on the DerSimonian and Laird method). A OR > 1 suggests that the aberrant β-catenin expression is associated with a higher T status. Diamonds indicate the pooled ORs with their corresponding 95% CIs. The forest plot was constructed using Stata software (v.16.1, Stata Corp, College Station, TX, USA).

6. Meta-Analysis on the Aberrant β-Catenin Expression and N Status in OSCC

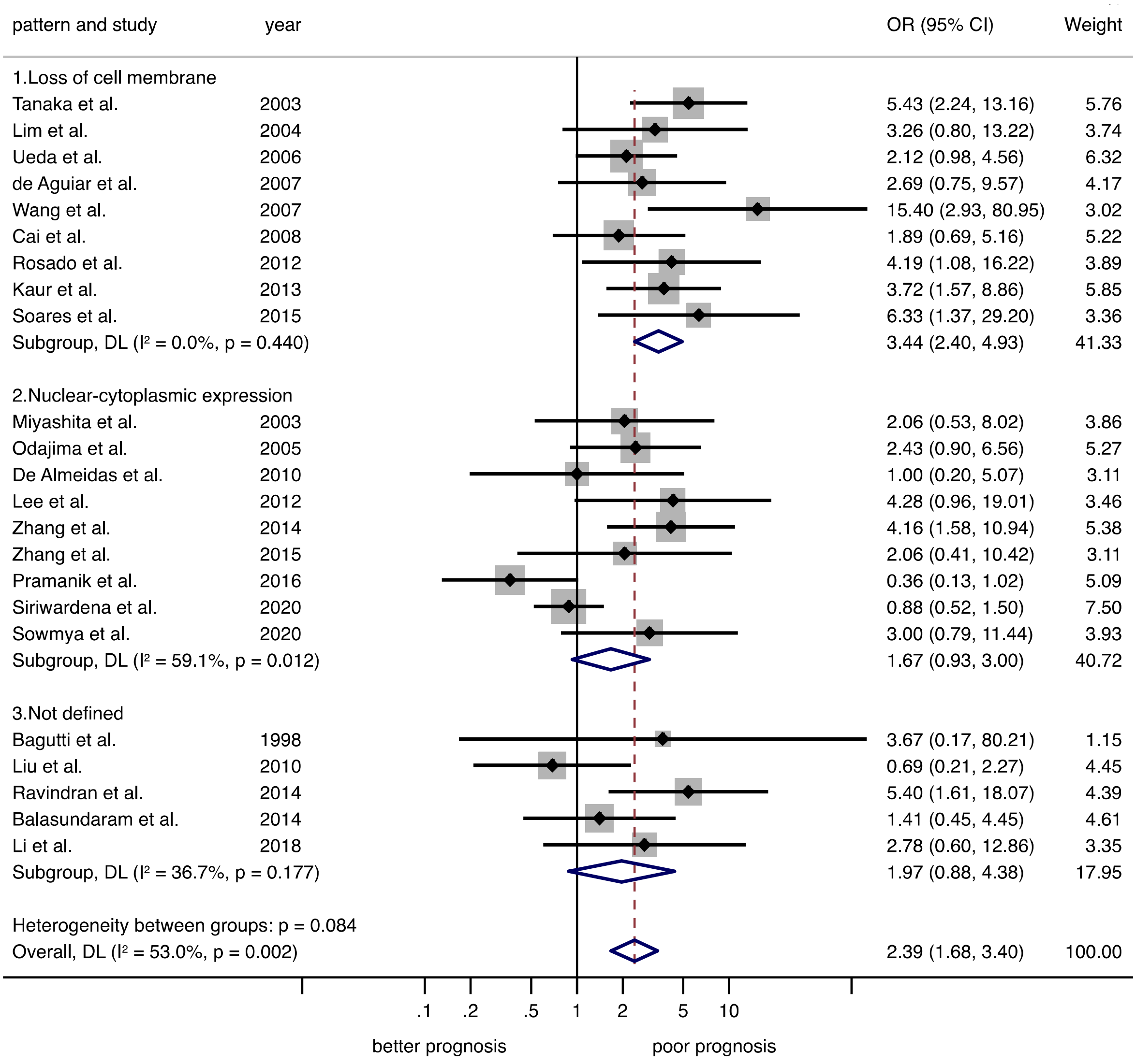

Figure S13. Forest plot graphically representing the stratified analysis by anti-β-catenin subcellular location on the association between the aberrant β-catenin expression and N status (positive metastatic lymph nodes vs. negative) in patients with OSCC. OSCC, oral squamous cell carcinoma; OR, odds ratio; CI, confidence intervals. Random-effects model, inverse-variance weighting (based on the DerSimonian and Laird method). A OR > 1 suggests that the aberrant β-catenin expression is associated with positive N status. Diamonds indicate the pooled ORs with their corresponding 95% CIs. The forest plot was constructed using Stata software (v.16.1, Stata Corp, College Station, TX, USA).

7. Meta-Analysis on the Aberrant β-Catenin Expression and Clinical Stage in OSCC

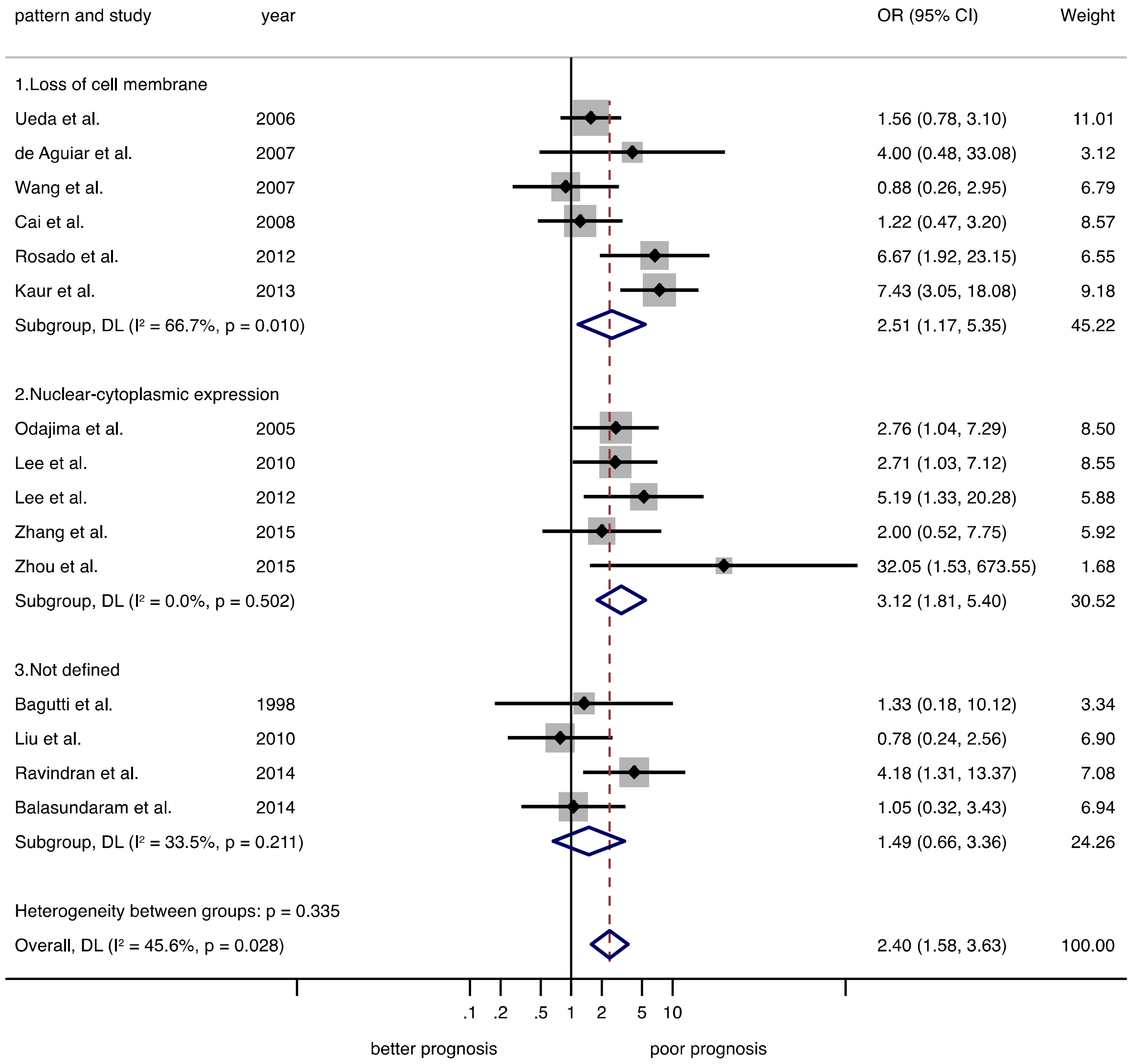

Figure S14. Forest plot graphically representing the stratified analysis by anti-β-catenin subcellular location on the association between the aberrant β-catenin expression and clinical stage (III/IV vs. I/II) in patients with OSCC. OSCC, oral squamous cell carcinoma; OR, odds ratio; CI, confidence intervals. Random-effects model, inverse-variance weighting (based on the DerSimonian and Laird method). A OR > 1 suggests that the aberrant β-catenin expression is associated with a higher stage. Diamonds indicate the pooled ORs with their corresponding 95% CIs. The forest plot was constructed using Stata software (v.16.1, Stata Corp, College Station, TX, USA).

8. Meta-Analysis on the Aberrant β-Catenin Expression and Histological Grade in OSCC

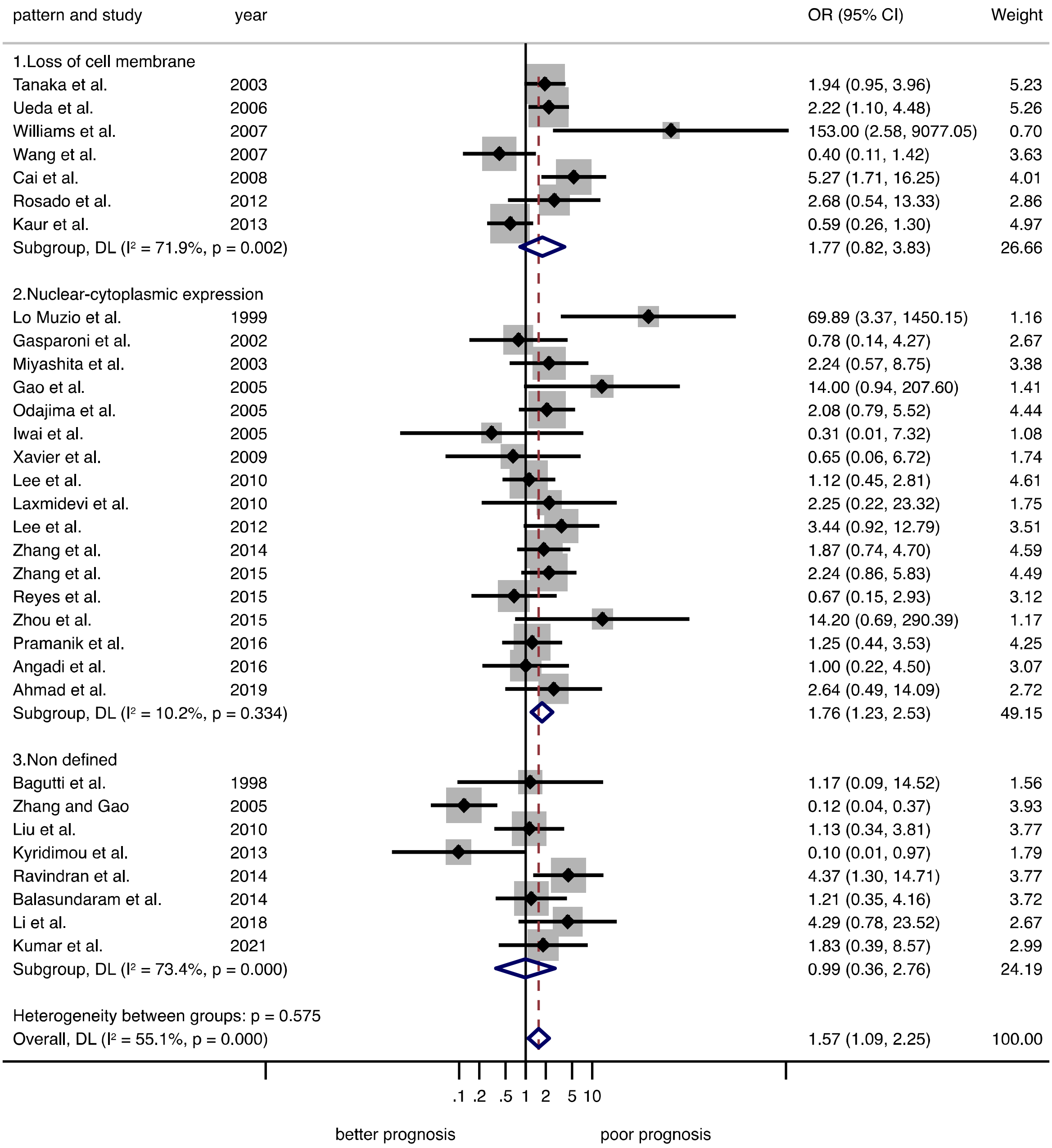

Figure S15. Forest plot graphically representing the stratified analysis by anti-β-catenin subcellular location on the association between the aberrant β-catenin expression and histological grade (poorly-moderate vs. well-differentiated carcinomas) in patients with OSCC. OSCC, oral squamous cell carcinoma; OR, odds ratio; CI, confidence intervals. Random-effects model, inverse-variance weighting (based on the DerSimonian and Laird method). A OR > 1 suggests that the aberrant β-catenin expression is associated with a higher grade. Diamonds indicate the pooled ORs with their corresponding 95% CIs. The forest plot was constructed using Stata software (v.16.1, Stata Corp, College Station, TX, USA).

9. Analysis of Small-Study Effects

9.1. Aberrant β-Catenin Expression and Overall Survival in OSCC

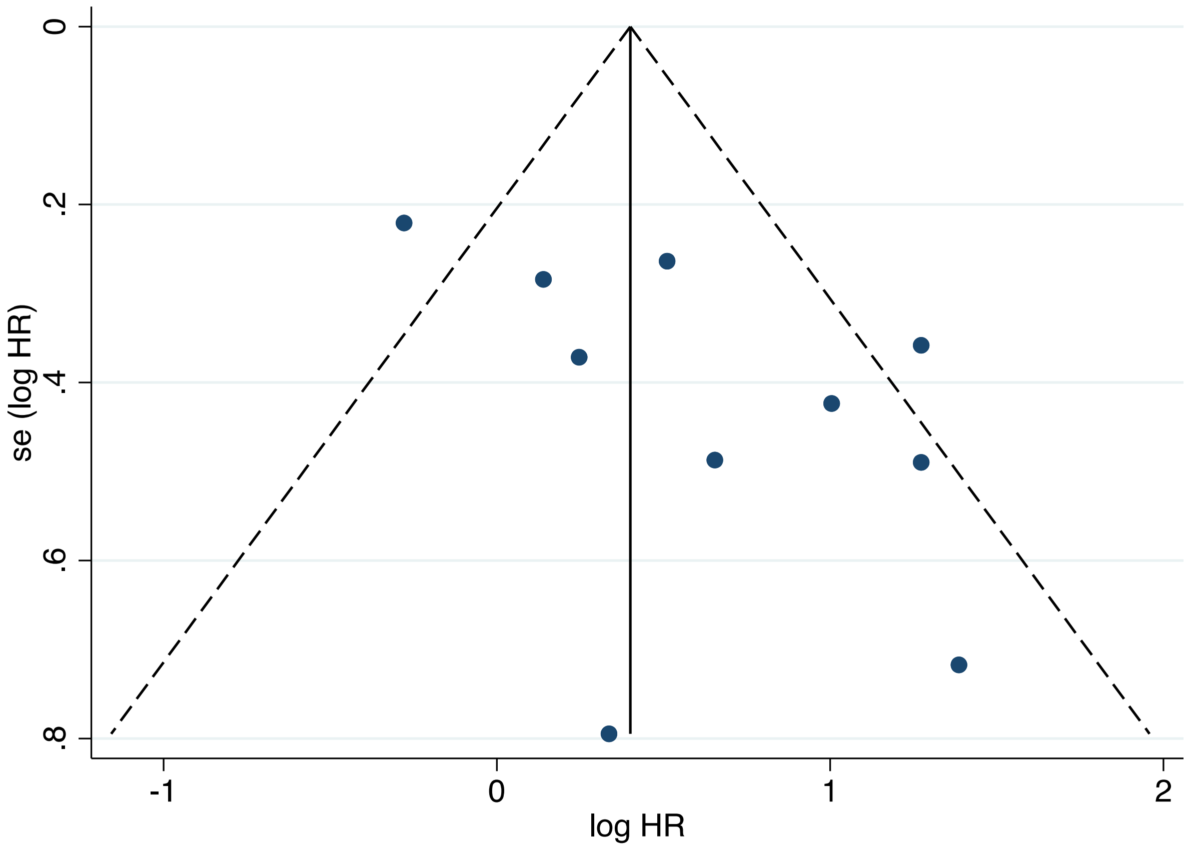

Figure S16. A funnel plot of estimated logHRs against their standard errors, graphically representing the analysis of small-study effects on the association between the aberrant β-catenin expression and overall survival in OSCC. SE, standard error; HR, hazard ratio; log, natural logarithm (i.e., log base e). The black vertical line corresponds to the pooled estimated prevalence. The two diagonal intermittent lines represent the pseudo−95% confidence interval. The blue circles represent the estimates from primary−level studies. The funnel plot was constructed using Stata software (v.16.1, Stata Corp, College Station, TX, USA).

9.2. Aberrant β-Catenin Expression and DFS in OSCC

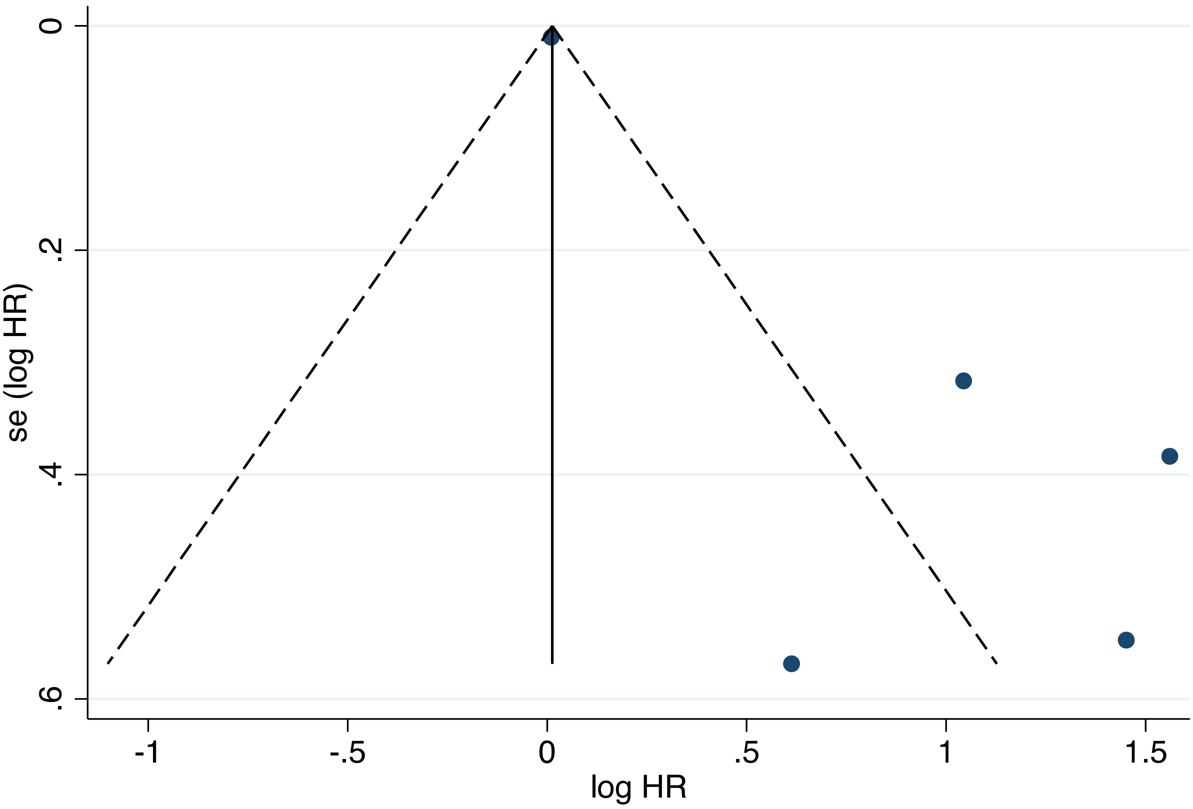

Figure S17. A funnel plot of estimated logHRs against their standard errors, graphically representing the analysis of small-study effects on the association between the aberrant β-catenin expression and DFS in OSCC. DFS, disease−free survival; SE, standard error; HR, hazard ratio; log, natural logarithm (i.e., log base e). The black vertical line corresponds to the pooled estimated prevalence. The two diagonal intermittent lines represent the pseudo−95% confidence interval. The blue circles represent the estimates from primary−level studies. The funnel plot was constructed using Stata software (v.16.1, Stata Corp, College Station, TX, USA).

9.3. Aberrant β-Catenin Expression and T Status in OSCC

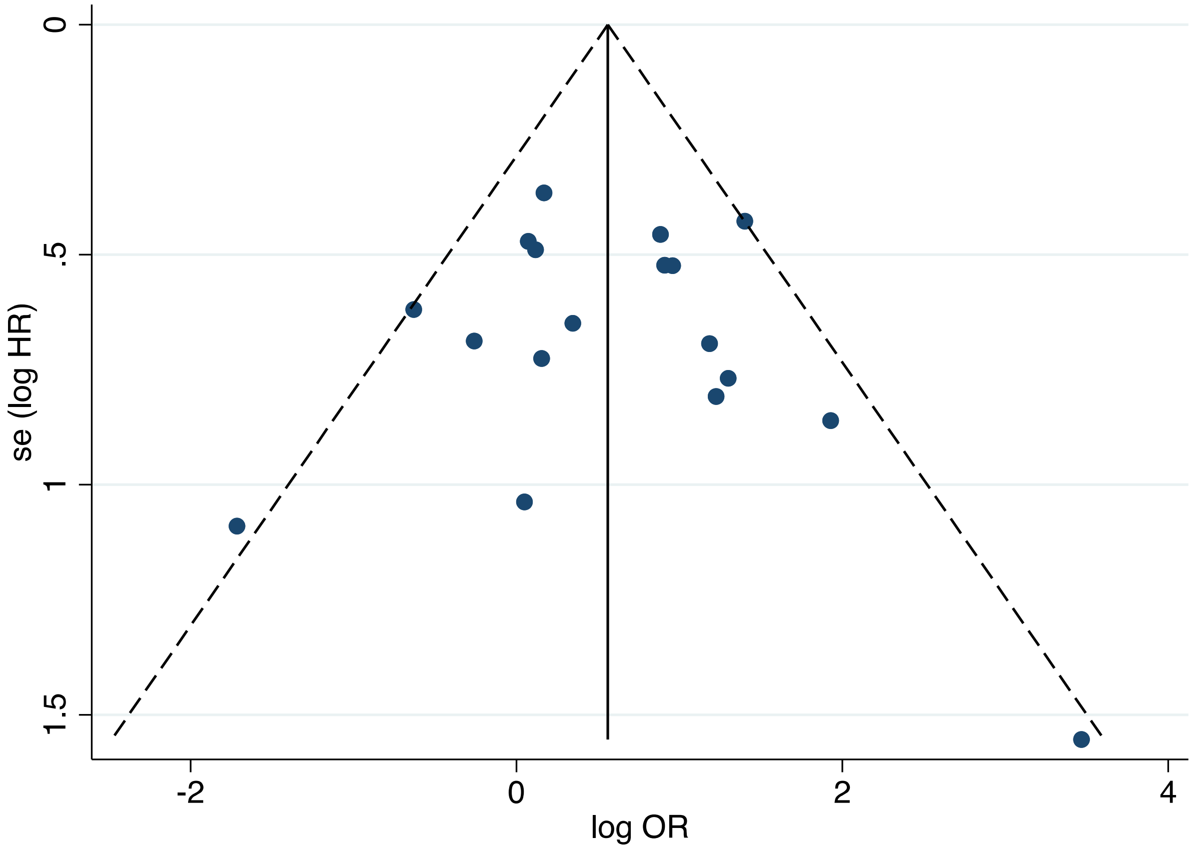

Figure S18. A funnel plot of estimated logORs against their standard errors, graphically representing the analysis of small−study effects on the association between the aberrant β-catenin expression and T status in OSCC. SE, standard error; OR, odds ratio; log, natural logarithm (i.e., log base e). The black vertical line corresponds to the pooled estimated prevalence. The two diagonal intermittent lines represent the pseudo−95% confidence interval. The blue circles represent the estimates from primary−level studies. The funnel plot was constructed using Stata software (v.16.1, Stata Corp, College Station, TX, USA).

9.4. Aberrant β-Catenin Expression and N Status in OSCC

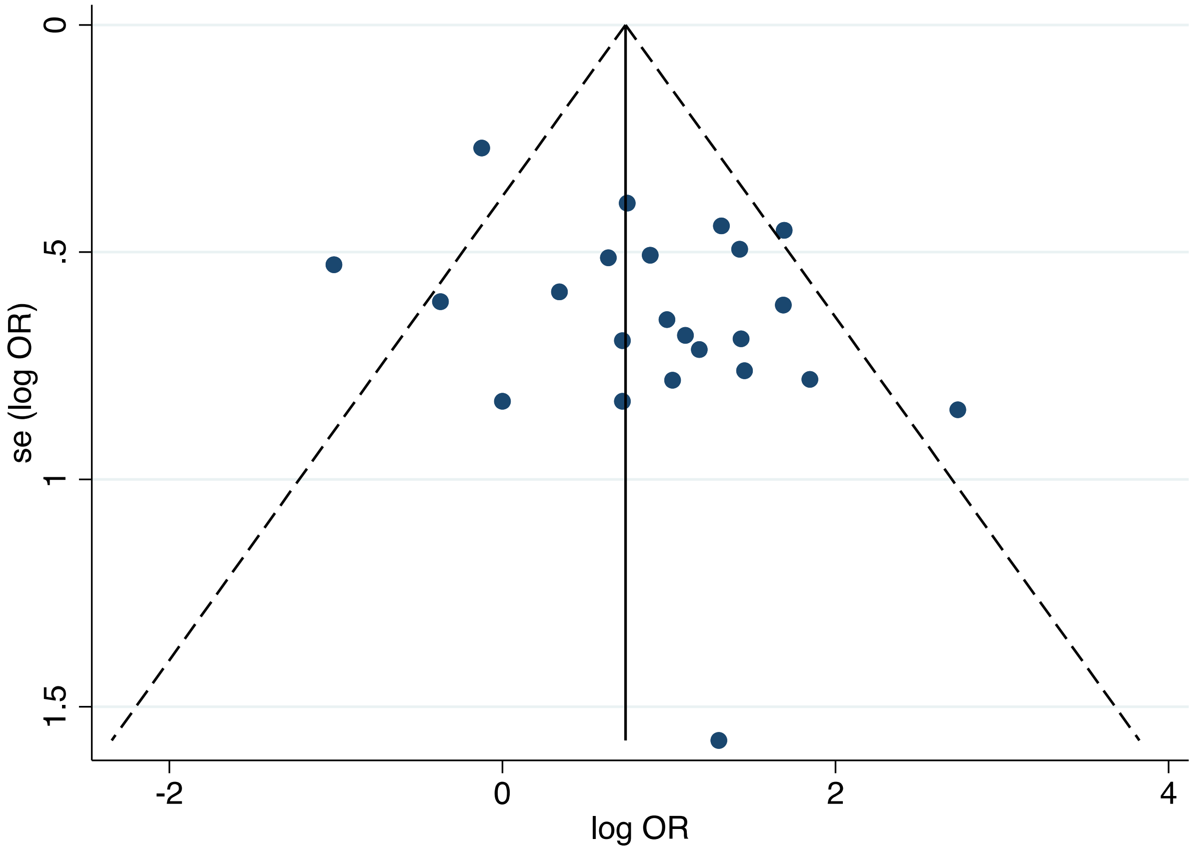

Figure S19. A funnel plot of estimated logORs against their standard errors, graphically representing the analysis of small−study effects on the association between the aberrant β-catenin expression and N status in OSCC. SE, standard error; OR, odds ratio; log, natural logarithm (i.e., log base e). The black vertical line corresponds to the pooled estimated prevalence. The two diagonal intermittent lines represent the pseudo−95% confidence interval. The blue circles represent the estimates from primary−level studies. The funnel plot was constructed using Stata software (v.16.1, Stata Corp, College Station, TX, USA).

9.5. Aberrant β-Catenin Expression and Clinical Stage in OSCC

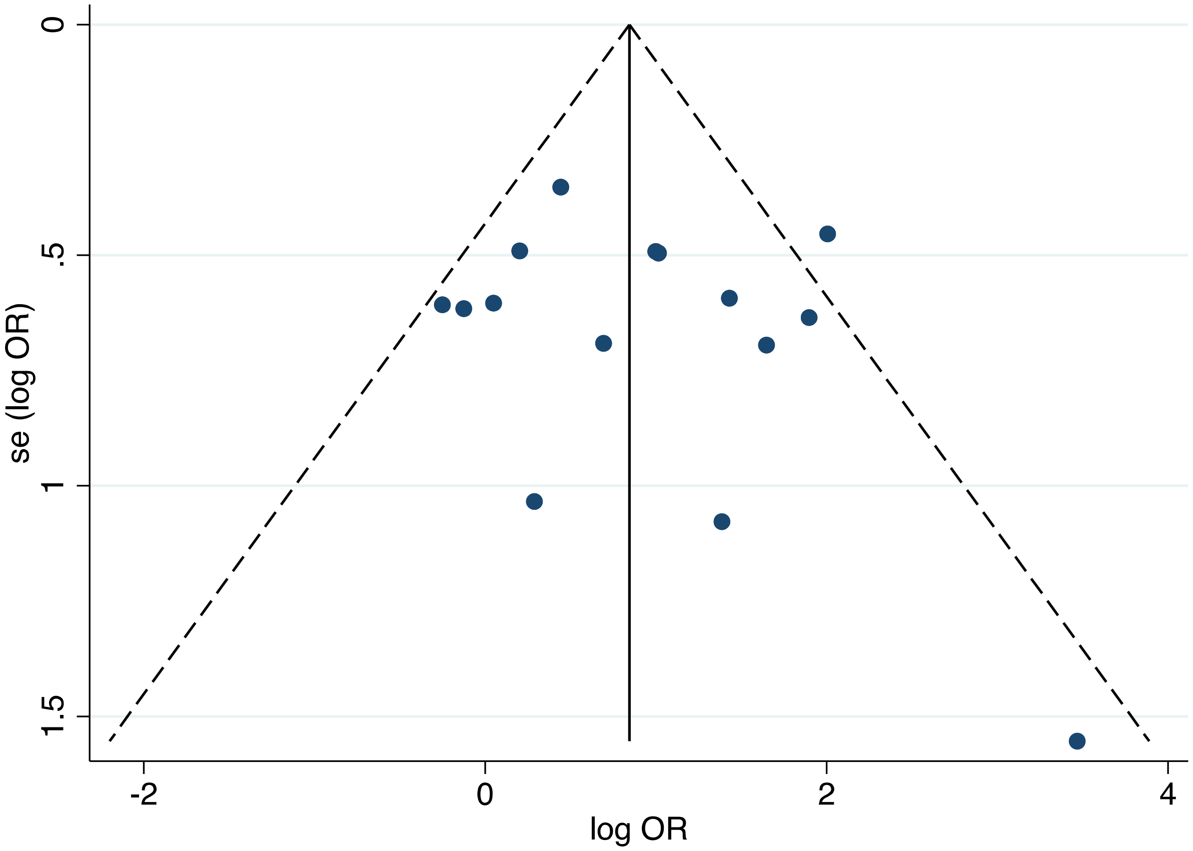

Figure S20. A funnel plot of estimated logORs against their standard errors, graphically representing the analysis of small−study effects on the association between the aberrant β-catenin expression and clinical stage in OSCC. SE, standard error; OR, odds ratio; log, natural logarithm (i.e., log base e). The black vertical line corresponds to the pooled estimated prevalence. The two diagonal intermittent lines represent the pseudo−95% confidence interval. The blue circles represent the estimates from primary−level studies. The funnel plot was constructed using Stata software (v.16.1, Stata Corp, College Station, TX, USA).

9.6. Aberrant β-catenin Expression and Histological Grade in OSCC

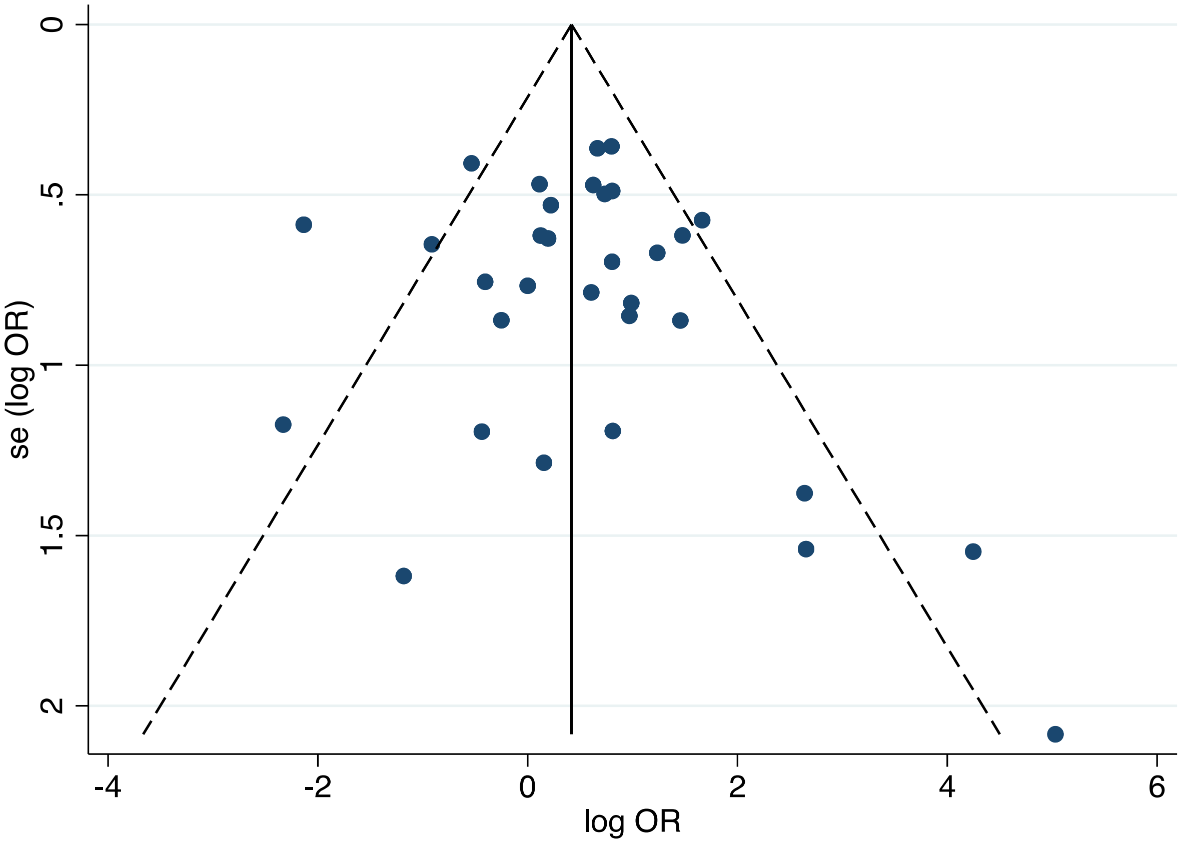

Figure S21. A funnel plot of estimated logORs against their standard errors, graphically representing the analysis of small−study effects on the association between the aberrant β-catenin expression and histological grade in OSCC. SE, standard error; OR, odds ratio; log, natural logarithm (i.e., log base e). The black vertical line corresponds to the pooled estimated prevalence. The two diagonal intermittent lines represent the pseudo−95% confidence interval. The blue circles represent the estimates from primary−level studies. The funnel plot was constructed using Stata software (v.16.1, Stata Corp, College Station, TX, USA).

10. Sensitivity Analysis (Leave-One-Out Method)

10.1. Aberrant β-Catenin Expression and Overall Survival in OSCC

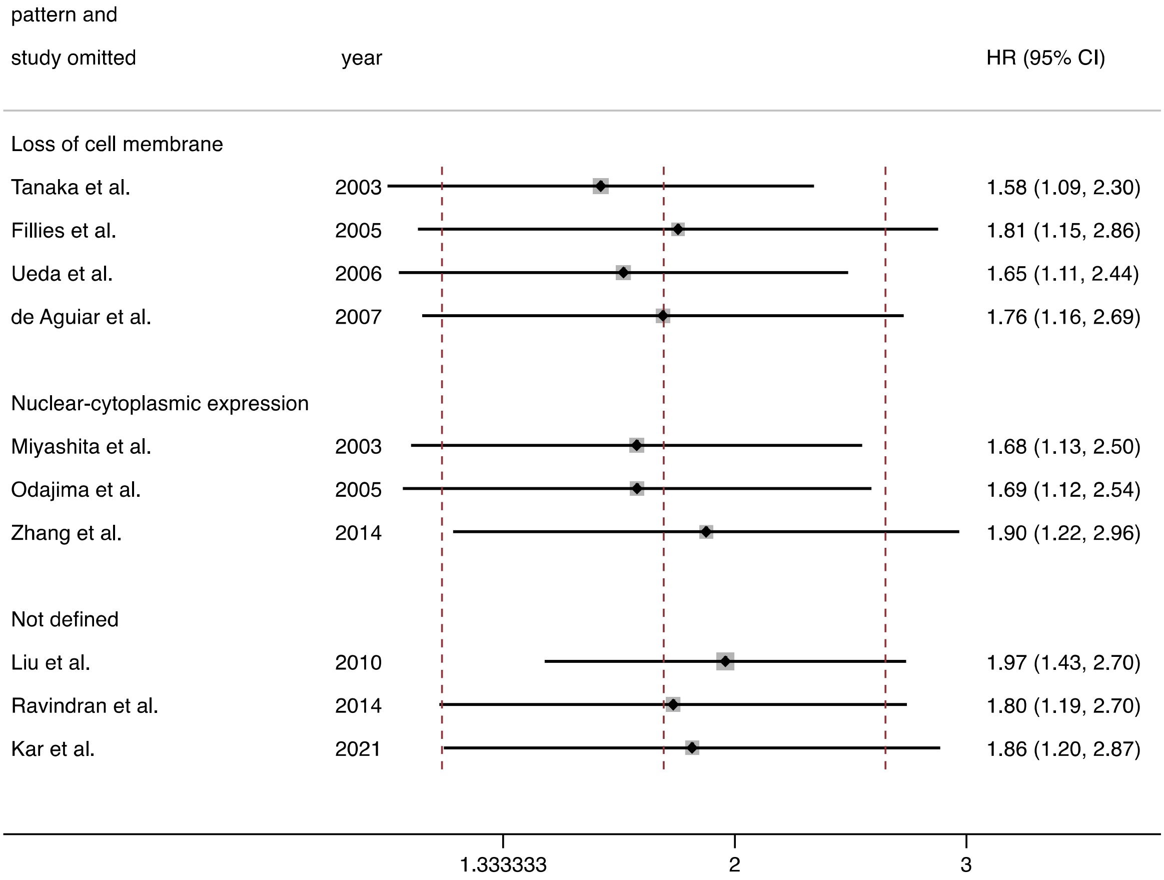

Figure S22. Interval plot graphically representing the sensitivity analysis of the studies pooled in the meta-analysis on the association between aberrant β-catenin expression and overall survival in OSCC. HR, hazard ratio; CI, confidence intervals. Sensitivity analysis ("leave-one-out" method) of the meta-analysis results, sequentially omitting one study at a time to investigate its influence on the overall result. In the interval plot, the usual diamond shape representing the pooled effect was replaced by vertical intermittent red. The interval plot was constructed using Stata software (v.16.1, Stata Corp, College Station, TX, USA).

10.2. Aberrant β-Catenin Expression and DFS in OSCC

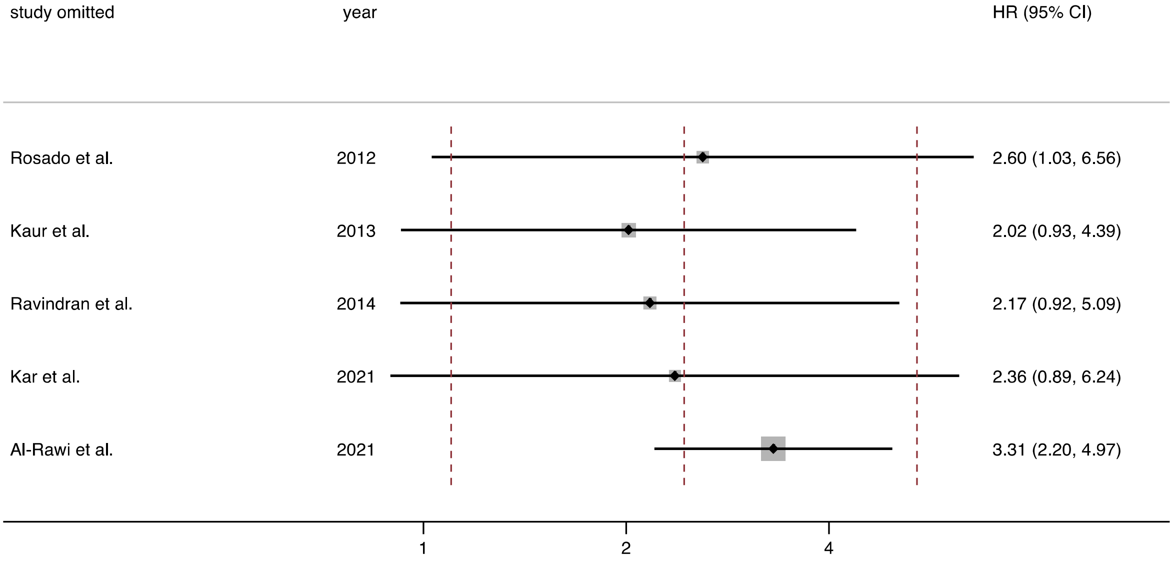

Figure S23. Interval plot graphically representing the sensitivity analysis of the studies pooled in the meta-analysis on the association between aberrant β-catenin expression and DFS in OSCC. DFS, disease-free survival; HR, hazard ratio; CI, confidence intervals. Sensitivity analysis ("leave-one-out" method) of the meta-analysis results, sequentially omitting one study at a time to investigate its influence on the overall result. In the interval plot, the usual diamond shape representing the pooled effect was replaced by vertical intermittent red lines, allowing a visual inspection analysis of influence. The interval plot was constructed using Stata software (v.16.1, Stata Corp, College Station, TX, USA).

10.3. Aberrant β-Catenin Expression and T Status in OSCC

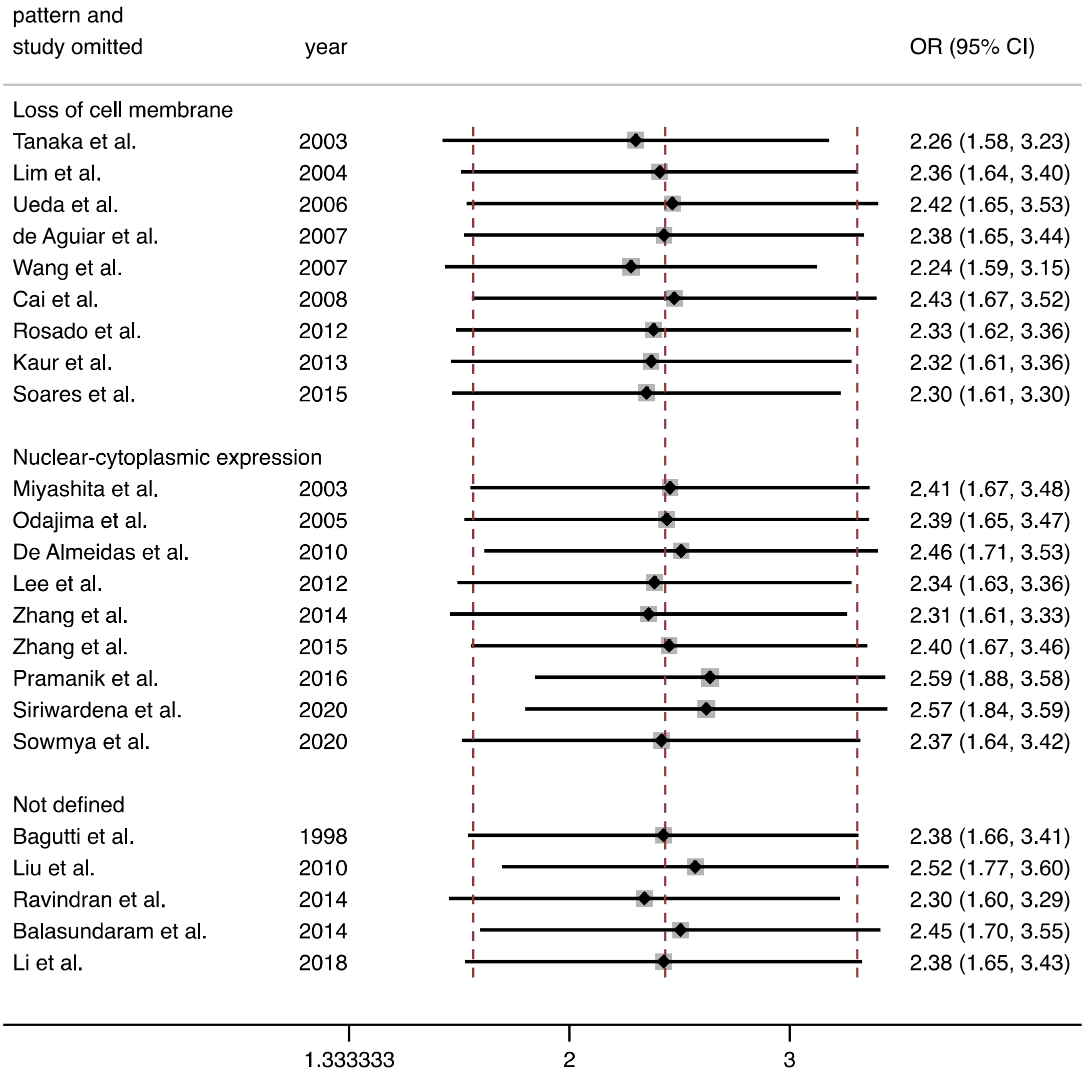

Figure S24. Interval plot graphically representing the sensitivity analysis of the studies pooled in the meta-analysis on the association between aberrant β-catenin expression and T status in OSCC. OR, odds ratio; CI, confidence intervals. Sensitivity analysis ("leave-one-out" method) of the meta-analysis results, sequentially omitting one study at a time to investigate its influence on the overall result. In the interval plot, the usual diamond shape representing the pooled effect was replaced by vertical intermittent red lines, allowing a visual inspection analysis of influence. The interval plot was constructed using Stata software (v.16.1, Stata Corp, College Station, TX, USA).

10.4. Aberrant β-Catenin Expression and N Status in OSCC

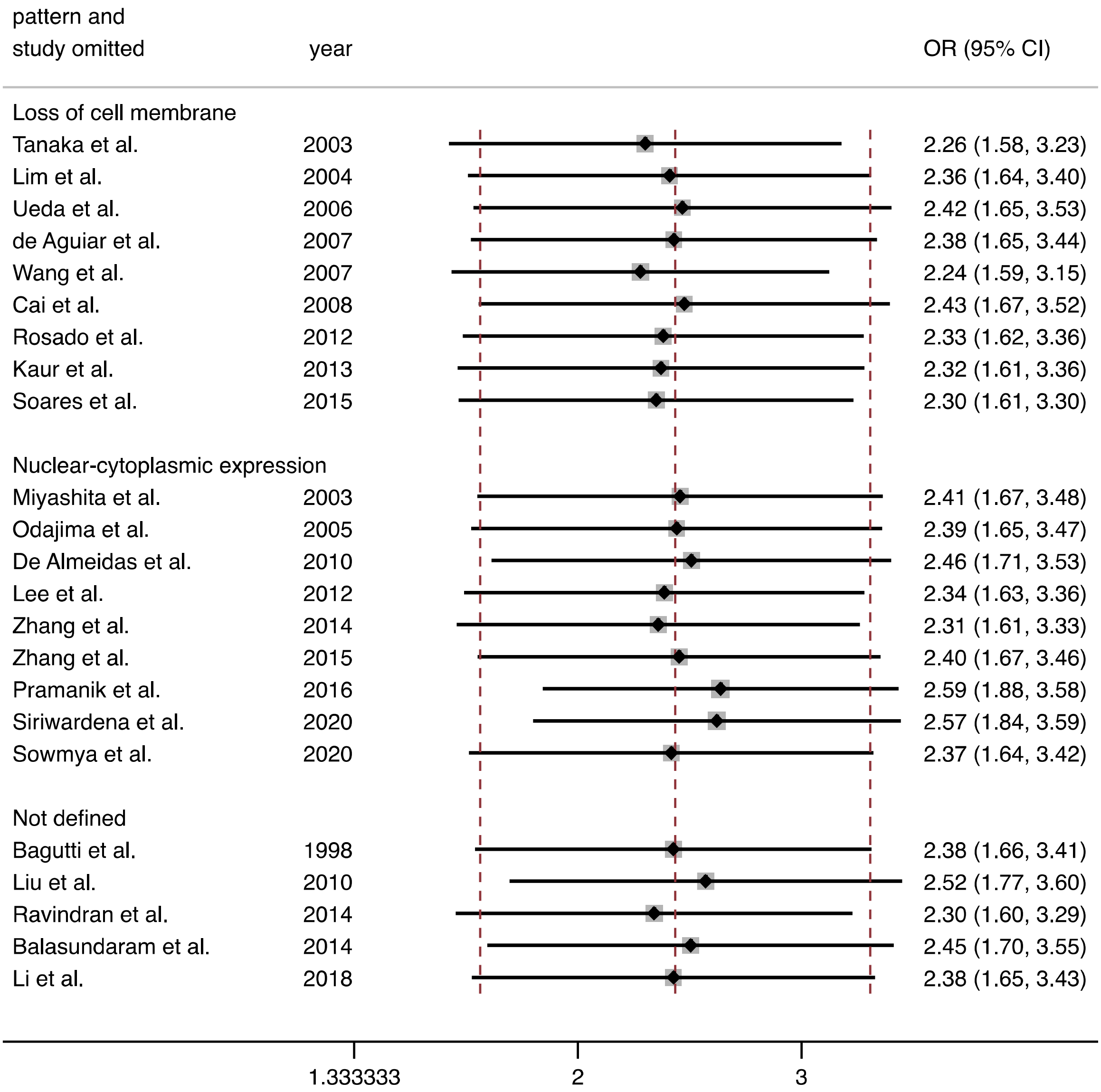

Figure S25. Interval plot graphically representing the sensitivity analysis of the studies pooled in the meta-analysis on the association between aberrant β-catenin expression and N status in OSCC. OR, odds ratio; CI, confidence intervals. Sensitivity analysis ("leave-one-out" method) of the meta-analysis results, sequentially omitting one study at a time to investigate its influence on the overall result. In the interval plot, the usual diamond shape representing the pooled effect was replaced by vertical intermittent red lines, allowing a visual. The interval plot was constructed using Stata software (v.16.1, Stata Corp, College Station, TX, USA).

10.5. Aberrant β-Catenin Expression and Clinical Stage in OSCC

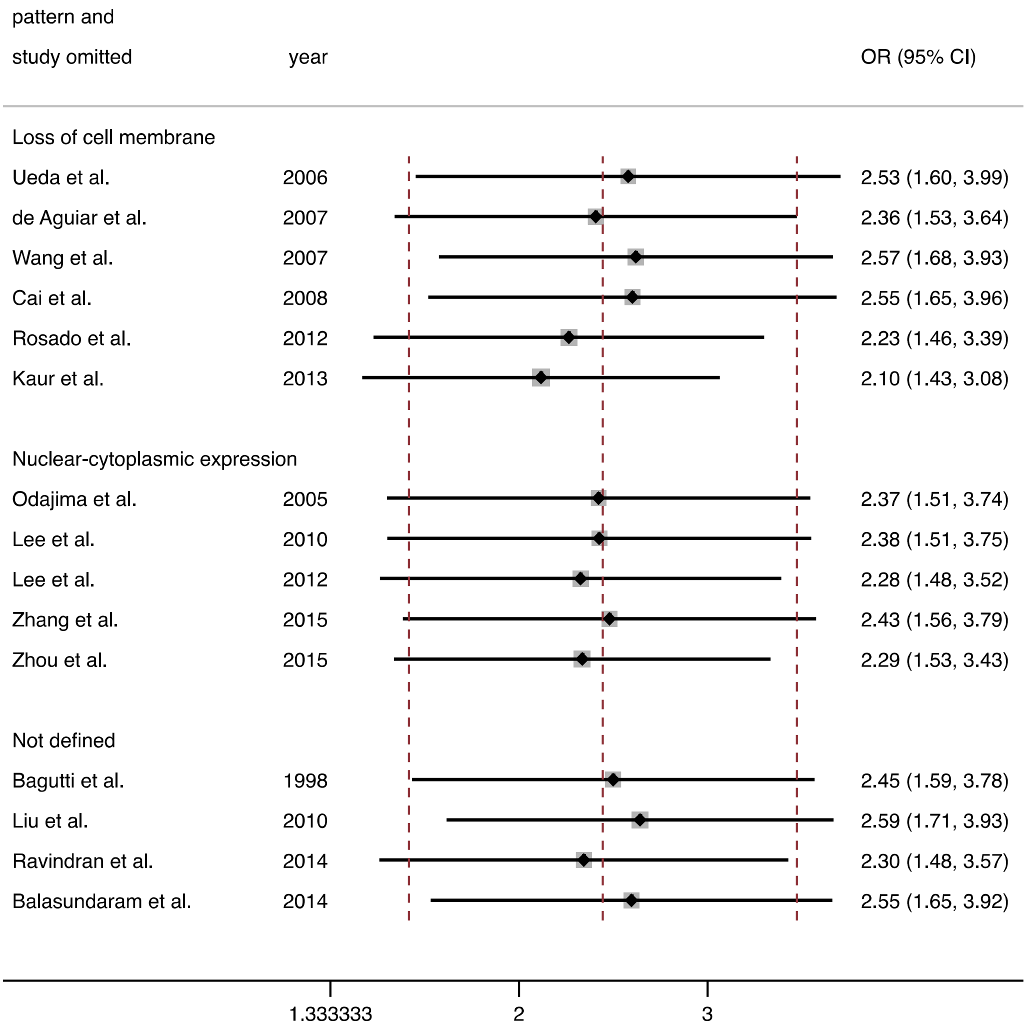

Figure S26. Interval plot graphically representing the sensitivity analysis of the studies pooled in the meta-analysis on the association between aberrant β-catenin expression and clinical stage in OSCC. OR, odds ratio; CI, confidence intervals. Sensitivity analysis ("leave-one-out" method) of the meta-analysis results, sequentially omitting one study at a time to investigate its influence on the overall result. In the interval plot, the usual diamond shape representing the pooled effect was replaced by vertical intermittent red lines, allowing a visual. The interval plot was constructed using Stata software (v.16.1, Stata Corp, College Station, TX, USA).

10.6. Aberrant β-Catenin Expression and Histological Grade in OSCC

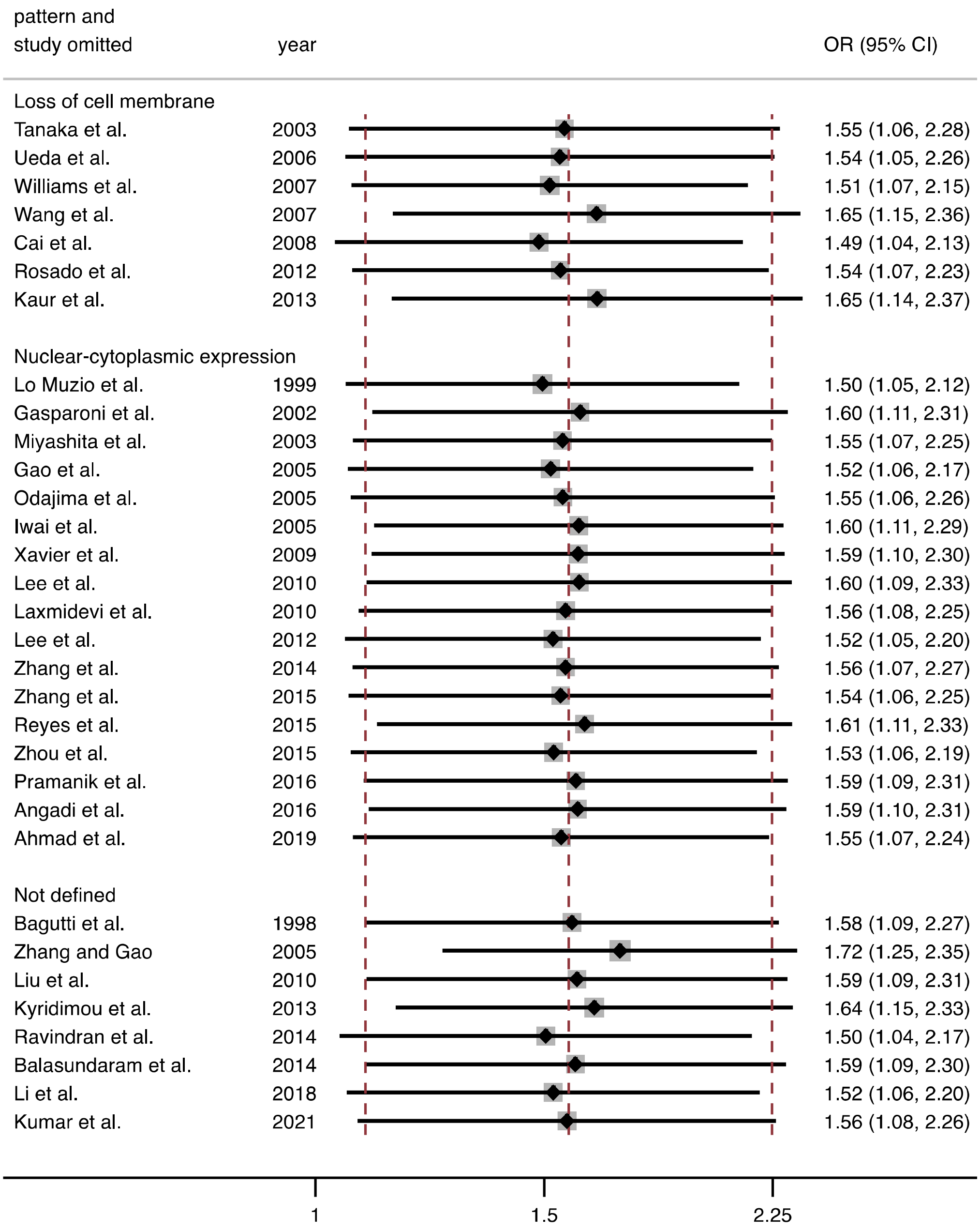

Figure S27. Interval plot graphically representing the sensitivity analysis of the studies pooled in the meta-analysis on the association between aberrant β-catenin expression and histological grade in OSCC. OR, odds ratio; CI, confidence intervals. Sensitivity analysis ("leave-one-out" method) of the meta-analysis results, sequentially omitting one study at a time to investigate its influence on the overall result. In the interval plot, the usual diamond shape representing the pooled effect was replaced by vertical intermittent red lines, allowing a visual. The interval plot was constructed using Stata software (v.16.1, Stata Corp, College Station, TX, USA).

11. Validation of Methodological Quality

11.1. AMSTAR2 Checklist

AMSTAR 2: a critical appraisal tool for systematic reviews that include randomised or non-randomised studies of healthcare interventions, or both

1. Did the research questions and inclusion criteria for the review include the components of PICO?

For Yes:
- ☑ Population
- ☑ Intervention
- ☑ Comparator group
- ☑ Outcome

Optional (recommended)
- ☑ Timeframe for follow-up

- ☑ Yes
- ☐ No

2. Did the report of the review contain an explicit statement that the review methods were established prior to the conduct of the review and did the report justify any significant deviations from the protocol?

For Partial Yes:
The authors state that they had a written protocol or guide that included ALL the following:
- ☑ review question(s)
- ☑ a search strategy
- ☑ inclusion/exclusion criteria
- ☑ a risk of bias assessment

For Yes:
As for partial yes, plus the protocol should be registered and should also have specified:
- ☑ a meta-analysis/synthesis plan, if appropriate, *and*
- ☑ a plan for investigating causes of heterogeneity
- ☑ justification for any deviations from the protocol

- ☑ Yes
- ☐ Partial Yes
- ☐ No

3. Did the review authors explain their selection of the study designs for inclusion in the review?

For Yes, the review should satisfy ONE of the following:
- ☐ *Explanation for* including only RCTs
- ☑ OR *Explanation for* including only NRSI
- ☐ OR *Explanation for* including both RCTs and NRSI

- ☑ Yes
- ☐ No

4. Did the review authors use a comprehensive literature search strategy?

For Partial Yes (all the following):
- ☑ searched at least 2 databases (relevant to research question)
- ☑ provided key word and/or search strategy
- ☑ justified publication restrictions (e.g. language)

For Yes, should also have (all the following):
- ☑ searched the reference lists / bibliographies of included studies
- ☐ searched trial/study registries — Not relevant for observational studies
- ☑ included/consulted content experts in the field
- ☐ where relevant, searched for grey literature — Considered not relevant and very controversial.
- ☑ conducted search within 24 months of completion of the review

- ☑ Yes
- ☐ Partial Yes
- ☐ No

5. Did the review authors perform study selection in duplicate?

For Yes, either ONE of the following:
- ☑ at least two reviewers independently agreed on selection of eligible studies and achieved consensus on which studies to include
- ☐ OR two reviewers selected a sample of eligible studies and achieved good agreement (at least 80 percent), with the remainder selected by one reviewer.

- ☑ Yes
- ☐ No

Figure S28. *Cont*.

AMSTAR 2: a critical appraisal tool for systematic reviews that include randomised or non-randomised studies of healthcare interventions, or both

6. Did the review authors perform data extraction in duplicate?

For Yes, either ONE of the following:

- ☑ at least two reviewers achieved consensus on which data to extract from included studies
- ☐ OR two reviewers extracted data from a sample of eligible studies and achieved good agreement (at least 80 percent), with the remainder extracted by one reviewer.

☑ Yes
☐ No

7. Did the review authors provide a list of excluded studies and justify the exclusions?

For Partial Yes:	For Yes, must also have:	
☑ provided a list of all potentially relevant studies that were read in full-text form but excluded from the review	☑ Justified the exclusion from the review of each potentially relevant study	☑ Yes<br>☐ Partial Yes<br>☐ No

8. Did the review authors describe the included studies in adequate detail?

For Partial Yes (ALL the following):	For Yes, should also have ALL the following:	
☑ described populations<br>☑ described interventions<br>☑ described comparators<br>☑ described outcomes<br>☑ described research designs	☑ described population in detail<br>☑ described intervention in detail (including doses where relevant)<br>☑ described comparator in detail (including doses where relevant)<br>☑ described study's setting<br>☑ timeframe for follow-up	☑ Yes<br>☐ Partial Yes<br>☐ No

9. Did the review authors use a satisfactory technique for assessing the risk of bias (RoB) in individual studies that were included in the review?

RCTs

For Partial Yes, must have assessed RoB from	For Yes, must also have assessed RoB from:	
☐ unconcealed allocation, *and*<br>☐ lack of blinding of patients and assessors when assessing outcomes (unnecessary for objective outcomes such as all-cause mortality)	☐ allocation sequence that was not truly random, *and*<br>☐ selection of the reported result from among multiple measurements or analyses of a specified outcome	☐ Yes<br>☐ Partial Yes<br>☐ No<br>☑ Includes only NRSI

NRSI

For Partial Yes, must have assessed RoB:	For Yes, must also have assessed RoB:	
☑ from confounding, *and*<br>☑ from selection bias	☑ methods used to ascertain exposures and outcomes, *and*<br>☑ selection of the reported result from among multiple measurements or analyses of a specified outcome	☑ Yes<br>☐ Partial Yes<br>☐ No<br>☐ Includes only RCTs

10. Did the review authors report on the sources of funding for the studies included in the review?

For Yes

- ☑ Must have reported on the sources of funding for individual studies included in the review. Note: Reporting that the reviewers looked for this information but it was not reported by study authors also qualifies

☑ Yes
☐ No

Figure S28. *Cont.*

AMSTAR 2: a critical appraisal tool for systematic reviews that include randomised or non-randomised studies of healthcare interventions, or both

11. If meta-analysis was performed did the review authors use appropriate methods for statistical combination of results?

RCTs
For Yes:

- ☐ The authors justified combining the data in a meta-analysis
  - ☐ AND they used an appropriate weighted technique to combine study results and adjusted for heterogeneity if present.
  - ☐ AND investigated the causes of any heterogeneity

☐ Yes
☐ No
☐ No meta-analysis conducted

For NRSI
For Yes:

- ☑ The authors justified combining the data in a meta-analysis
  - ☑ AND they used an appropriate weighted technique to combine study results, adjusting for heterogeneity if present
  - ☑ AND they statistically combined effect estimates from NRSI that were adjusted for confounding, rather than combining raw data, or justified combining raw data when adjusted effect estimates were not available
  - ☑ AND they reported separate summary estimates for RCTs and NRSI separately when both were included in the review

☑ Yes
☐ No
☐ No meta-analysis conducted

12. If meta-analysis was performed, did the review authors assess the potential impact of RoB in individual studies on the results of the meta-analysis or other evidence synthesis?

For Yes:

- ☐ included only low risk of bias RCTs
- ☑ OR, if the pooled estimate was based on RCTs and/or NRSI at variable RoB, the authors performed analyses to investigate possible impact of RoB on summary estimates of effect.

☑ Yes
☐ No
☐ No meta-analysis conducted

13. Did the review authors account for RoB in individual studies when interpreting/ discussing the results of the review?

For Yes:

- ☐ included only low risk of bias RCTs
- ☑ OR, if RCTs with moderate or high RoB, or NRSI were included the review provided a discussion of the likely impact of RoB on the results

☑ Yes
☐ No

14. Did the review authors provide a satisfactory explanation for, and discussion of, any heterogeneity observed in the results of the review?

For Yes:

- ☐ There was no significant heterogeneity in the results
- ☑ OR if heterogeneity was present the authors performed an investigation of sources of any heterogeneity in the results and discussed the impact of this on the results of the review

☑ Yes
☐ No

15. If they performed quantitative synthesis did the review authors carry out an adequate investigation of publication bias (small study bias) and discuss its likely impact on the results of the review?

For Yes:

- ☑ performed graphical or statistical tests for publication bias and discussed the likelihood and magnitude of impact of publication bias

☑ Yes
☐ No
☐ No meta-analysis conducted

Figure S28. *Cont.*

AMSTAR 2: a critical appraisal tool for systematic reviews that include randomised or non-randomised studies of healthcare interventions, or both

16. Did the review authors report any potential sources of conflict of interest, including any funding they received for conducting the review?

For Yes:

- ☑ The authors reported no competing interests OR
- ☐ The authors described their funding sources and how they managed potential conflicts of interest

☑ Yes
☐ No

To cite this tool: Shea BJ, Reeves BC, Wells G, Thuku M, Hamel C, Moran J, Moher D, Tugwell P, Welch V, Kristjansson E, Henry DA. AMSTAR 2: a critical appraisal tool for systematic reviews that include randomised or non-randomised studies of healthcare interventions, or both. BMJ. 2017 Sep 21;358:j4008.

Figure S28. AMSTAR2 checklist.

11.2. Validation of Methodological Quality

Table S3. AMSTAR2 scoring system.

Tool	Study Design	Items																Overall Rating	Score
AMSTAR2	Systematic review and meta-analysis	1	2	3	4	5	6	7	8	9	10	11	12	13	14	15	16	HIGH	16

Explanation: The methodological quality of this systematic review followed the *Assessing the Methodological Quality of Systematic Reviews-2* (AMSTAR2) recommendations and was validated using this tool. AMSTAR2 was designed to develop, evaluate and validate high quality systematic reviews through 16 items. An overall rating is obtained based on weaknesses (*) in the following critical and non-critical items (the checklist was also included in the precedent appendix page):

1. Did the research questions and inclusion criteria for the review include the components of PICO?
2. Did the report of the review contain an explicit statement that the review methods were established prior to the conduct of their review, and did the report justify any significant deviations from the protocol? *
3. Did the review authors explain their selection of the study designs for inclusion in the review?
4. Did the review authors use a comprehensive literature search strategy? *
5. Did the review authors perform study selection in duplicate?
6. Did the review authors perform data extraction in duplicate?
7. Did the review authors provide a list of excluded studies and justify the exclusions?*
8. Did the review authors describe the included studies in adequate detail?
9. Did the review authors use a satisfactory technique for assessing the risk of bias (RoB) in individual studies that were included in the review? *
10. Did the review authors report on the sources of funding for the studies included in the review?
11. If meta-analysis was performed, did the review authors use appropriate methods for statistical combination of results? *
12. If meta-analysis was performed, did the review authors assess the potential impact of RoB in individual studies on the results of the metaanalysis or other evidence synthesis?
13. Did the review authors account for RoB in individual studies when interpreting/discussing the results of the review? *
14. Did the review authors provide a satisfactory explanation for, and discussion of, any heterogeneity observed in the results of the review?

15. If they performed quantitative synthesis, did the review authors carry out an adequate investigation of publication bias (small study bias) and discuss its likely impact on the results of the review? *
16. Did the review authors report any potential sources of conflict of interest, including any funding they received for conducting the review?

High overall rating: No or one non-critical weakness. The systematic review provides an accurate and comprehensive summary of the results of the available studies that address the question of interest.

12. List of Full-Text Articles Excluded with Reasons

12.1. Lack of Essential Data (n = 21)

1. Andrews, N.A.; Jones, A.S.; Helliwell, T.R.; Kinsella, A.R. Expression of the E-cadherin-catenin cell adhesion complex in primary squamous cell carcinomas of the head and neck and their nodal metastases. *Br. J. Cancer.* **1997**, *75*, 1474–1480, doi:10.1038/bjc.1997.252.
2. Bánkfalvi, A.; Kraβort, M.; Végh, A.; Felszeghy, E.; Piffkó, J. Deranged expression of the E-cadherin/β-catenin complex and the epidermal growth factor receptor in the clinical evolution and progression of oral squamous cell carcinomas. *J. Oral Pathol. Med.* **2002**, *31*, 450–457, doi:10.1034/j.1600-0714.2002.00147.x.
3. Barakat, C. B-Catenin Alterations in Squamous Cell Carcinoma of the Lip. Asian Pacific J. *Cancer Prev.* **2015**, *16*, 5187–5190, doi:10.7314/APJCP.2015.16.13.5187.
4. Cavicchioli Buim, M.E.; Gurgel, C.A.S.; Gonçalves Ramos, E.A.; Lourenço, S.V.; Soares, F.A. Activation of sonic hedgehog signaling in oral squamous cell carcinomas: A preliminary study. *Hum. Pathol.* **2011**, *42*, 1484–1490, doi:10.1016/j.humpath.2010.12.015.
5. Chow, V.; Yuen, A.P.; Lam, K.Y.; Tsao, G.S.; Ho, W.K.; Wei, W.I. A comparative study of the clinicopathological significance of E-cadherin and catenins (alpha, beta, gamma) expression in the surgical management of oral tongue carcinoma. *J. Cancer. Res. Clin. Oncol.* **2001**, *127*, 59–63.
6. Dantas, R.C.M.; Guimarães, V.S.N.; de Souza, R.O.; Valverde, L.F.; Vidal, M.T.A.; Nogueira, R.L.R.; da Rocha, L.O.S.; Araújo, G.T.; dos Santos, J.N.; Rocha, C.A.G. Immunodetection of epithelial-mesenchymal transition and tumor proliferation markers in gli-1-positive oral squamous cell carcinoma. *Appl. Immunohistochem. Mol. Morphol.* **2021**, *29*, 335–344, doi:10.1097/PAI.0000000000000866.
7. Frohwitter, G.; Buerger, H.; Korsching, E.; van Diest, P.J.; Kleinheinz, J.; Fillies, T. Site-specific gene expression patterns in oral cancer. *Head Face Med.* **2017**, *13*, doi:10.1186/s13005-017-0138-0.
8. Fujii, M.; Katase, N.; Lefeuvre, M.; Gunduz, M.; Buery, R.R.; Tamamura, R.; Tsujigiwa, H.; Nagatsuka, H. Dickkopf (Dkk)-3 and β-catenin expressions increased in the transition from normal oral mucosal to oral squamous cell carcinoma. *J. Mol. Histol.* **2011**, *42*, 499–504, doi:10.1007/s10735-011-9357-z.
9. Jot, K.; Urs, A.B.; Kumar, P. Does Loss of Immunohistochemical Expression of Glypican 3 in Oral Squamous Cell Carcinoma Play a Role in the Wnt/β-catenin Signaling Pathway? *Appl. Immunohistochem. Mol. Morphol. AIMM*. **2021**, *29*, 693–699, doi:10.1097/PAI.0000000000000955.
10. Li, S.; Jiao, J.; Lu, Z.; Zhang, M. An essential role for N-cadherin and β-catenin for progression in tongue squamous cell carcinoma and their effect on invasion and metastasis of Tca8113 tongue cancer cells. *Oncol. Rep.* **2009**, *21*, 1223–1233, doi:10.3892/or_00000345.

11. Lin, Y.M.; Chen, M.L.; Chen, C. Lo; Yeh, C.M.; Sung, W.W. Overexpression of EIF5A2 predicts poor prognosis in patients with oral squamous cell carcinoma. *Diagn.* **2020**, *10*, doi:10.3390/diagnostics10070436.
12. Lopes, F.F.; da Costa Miguel, M.C.; Pereira, A.L.A.; da Cruz, M.C.F.N.; de Almeida Freitas, R.; Pinto, L.P.; de Souza, L.B. Changes in immunoexpression of E-cadherin and β-catenin in oral squamous cell carcinoma with and without nodal metastasis. *Ann. Diagn. Pathol.* **2009**, *13*, 22–29, doi:10.1016/j.anndiagpath.2008.07.006.
13. Närkiö-Mäkelä, M.; Pukkila, M.; Lagerstedt, E.; Virtaniemi, J.; Pirinen, R.; Johansson, R.; Kosunen, A.; Lappalainen, K.; Hämäläinen, K.; Kosma, V.-M. Reduced γ-Catenin Expression and Poor Survival in Oral Squamous Cell Carcinoma. *Arch. Otolaryngol. Neck Surg.* **2009**, *135*, 1035, doi:10.1001/archoto.2009.132.
14. Pereira, C.H.; Morais, M.O.; Martins, A.F.L.; Soares, M.Q.S.; Alencar, R.D.C.G.; Batista, A.C.; Leles, C.R.; Mendonça, E.F. Expression of adhesion proteins (E-cadherin and β-catenin) and cell proliferation (Ki-67) at the invasive tumor front in conventional oral squamous cell and basaloid squamous cell carcinomas. *Arch. Oral Biol.* **2016**, *61*, 8–15, doi:10.1016/j.archoralbio.2015.10.003.
15. Roy, S.; Kar, M.; Roy, S.; Padhi, S.; Saha, A.; Banerjee, B. KLF4 expression in the surgical cut margin is associated with disease relapse of oral squamous cell carcinoma. *Oral Surg. Oral Med. Oral Pathol. Oral Radiol.* **2019**, *128*, 154–165, doi:10.1016/j.oooo.2019.02.021.
16. Santoro, A.; Pannone, G.; Papagerakis, S.; McGuff, H.S.; Cafarelli, B.; Lepore, S.; De Maria, S.; Rubini, C.; Mattoni, M.; Staibano, S.; et al. Beta-catenin and epithelial tumors: A study based on 374 oropharyngeal cancers. *Biomed Res. Int.* **2014**, *2014*, doi:10.1155/2014/948264.
17. Sasaya, K.; Sudo, H.; Maeda, G.; Kawashiri, S.; Imai, K. Concomitant Loss of p120-Catenin and β-Catenin Membrane Expression and Oral Carcinoma Progression with E-Cadherin Reduction. *PLoS One* **2013**, *8*, doi:10.1371/journal.pone.0069777.
18. Schneider, F.T.; Schänzer, A.; Czupalla, C.J.; Thom, S.; Engels, K.; Schmidt, M.H.H.; Plate, K.H.; Liebner, S. Sonic hedgehog acts as a negative regulator of β-catenin signaling in the adult tongue epithelium. *Am. J. Pathol.* **2010**, *177*, 404–414, doi:10.2353/ajpath.2010.091079.
19. Sgaramella, N.; Wilms, T.; Boldrup, L.; Loljung, L.; Gu, X.; Coates, P.J.; Hassellöf, P.; Califano, L.; Muzio, L. Lo; Fåhraeus, R.; et al. Ethnicity based variation in expression of E-cadherin in patients with squamous cell carcinoma of the oral tongue. *Oncol. Lett.* **2018**, *16*, 6603–6607, doi:10.3892/ol.2018.9452.
20. Zaid, K.W. Immunohistochemical assessment of E-cadherin and β-catenin in the histological differentiations of oral squamous cell carcinoma. *Asian Pacific J. Cancer Prev.* **2014**, *15*, 8847–8853, doi:10.7314/APJCP.2014.15.20.8847.
21. Zargaran, M. Alternation of β-catenin and CD44s immunoexpression in different histopathological grades of oral squamous cell carcinoma. *Asian Pacific J. Cancer Prev.* **2020**, *21*, 1181–1185, doi:10.31557/APJCP.2020.21.5.1181.

12.2. No Clinico-Pathological Outcomes (n = 12)

1. Bazarsad, S.; Zhang, X.; Kim, K.Y.; Illeperuma, R.; Jayasinghe, R.D.; Tilakaratne, W.M.; Kim, J. Identification of a combined biomarker for malignant transformation in oral submucous fibrosis. *J. Oral Pathol. Med.* **2017**, *46*, 431–438, doi:10.1111/jop.12483.
2. Chaw, S.Y.; Abdul Majeed, A.; Dalley, A.J.; Chan, A.; Stein, S.; Farah, C.S. Epithelial to mesenchymal transition (EMT) biomarkers – E-cadherin, beta-catenin, APC and Vimentin – in oral squamous cell carcinogenesis and transformation. *Oral Oncol.* **2012**, *48*, 997–1006, doi:10.1016/j.oraloncology.2012.05.011.
3. Ishida, K.; Ito, S.; Wada, N.; Deguchi, H.; Hata, T.; Hosoda, M.; Nohno, T. Nuclear localization of beta-catenin involved in precancerous change in oral leukoplakia. *Mol. Cancer.* **2007**, *6*, doi:10.1186/1476-4598-6-62.
4. Krüger, M.; Amort, J.; Wilgenbus, P.; Helmstädter, J.P.; Grechowa, I.; Ebert, J.; Tenzer, S.; Moergel, M.; Witte, I.; Horke, S. The anti-apoptotic PON2 protein is Wnt/β-catenin-regulated and correlates with radiotherapy resistance in OSCC patients. *Oncotarget*. **2016**, 7, 51082–51095, doi:10.18632/oncotarget.9013.
5. Kudo, Y.; Kitajima, S.; Ogawa, I.; Hiraoka, M.; Sargolzaei, S.; Keikhaee, M.R.; Sato, S.; Miyauchi, M.; Takata, T. Invasion and metastasis of oral cancer cells require methylation of E-cadherin and/or degradation of membranous β-catenin. *Clin. Cancer Res.* **2004**, *10*, 5455–5463, doi:10.1158/1078-0432.CCR-04-0372.
6. Leung, K.W.; Tsai, C.H.; Hsiao, M.; Tseng, C.J.; Ger, L.P.; Lee, K.H.; Lu, P.J. Pin1 overexpression is associated with poor differentiation and survival in oral squamous cell carcinoma. *Oncol. Rep.* **2009**, *21*, 1097–1104, doi:10.3892/or_00000329.
7. Prgomet, Z.; Andersson, T.; Lindberg, P. Higher expression of WNT5A protein in oral squamous cell carcinoma compared with dysplasia and oral mucosa with a normal appearance. *Eur. J. Oral Sci.* **2017**, *125*, 237–246, doi:10.1111/eos.12352.
8. Schussel, J.L.; Pinto, D.D.S.; Martins, M.T. Altered β-catenin expression related to cancer progression on actinic cheilitis and squamous cell carcinoma of the lip. *Ann. Diagn. Pathol.* **2011**, *15*, 1–5, doi:10.1016/j.anndiagpath.2010.07.002.
9. Tsuchiya R, Yamamoto G, Nagoshi Y, Aida T, Irie T, Tachikawa T. Expression of adenomatous polyposis coli (APC) in tumorigenesis of human oral squamous cell carcinoma. *Oral Oncol.* **2004**, *40*, 932–940.
10. Uraguchi, M.; Morikawa, M.; Shirakawa, M.; Sanada, K.; Imai, K. Activation of WNT family expression and signaling in squamous cell carcinomas of the oral cavity. *J. Dent. Res.* **2004**, *83*, 327–332, doi:10.1177/154405910408300411.
11. Yeh, K.T.; Chang, J.G.; Lin, T.H.; Wang, Y.F.; Chang, J.Y.; Shih, M.C.; Lin, C.C. Correlation between protein expression and epigenetic and mutation changes of Wnt pathway-related genes in oral cancer. *Int. J. Oncol*. **2003**, *23*, 1001–1007, doi:10.3892/ijo.23.4.1001.
12. M., Z.; F., B.; A., M. Comparative study of $β$-catenin and CD44 immunoexpression in oral lichen planus and squamous cell carcinoma. *Int. J. Dermatol.* **2018**, *57*, 794–798.

12.3. In Vitro (n = 10)

1. Qiao, C.; Qiao, T.; Yang, S.; Liu, L.; Zheng, M. SNHG17/miR-384/ELF1 axis promotes cell growth by transcriptional regulation of CTNNB1 to activate Wnt/β-catenin pathway in oral squamous cell carcinoma. *Cancer Gene Ther.* **2021**, doi:10.1038/s41417-021-00294-9.
2. Reyes, M.; Peña-Oyarzun, D.; Maturana, A.; Torres, V.A. Nuclear localization of β-catenin and expression of target genes are associated with increased Wnt secretion in oral dysplasia. *Oral Oncol.* **2019**, *94*, 58–67, doi:10.1016/j.oraloncology.2019.05.010.
3. Marimuthu, M.; Andiappan, M.; Wahab, A.; Muthusekhar, M.; Balakrishnan, A.; Shanmugam, S. Canonical Wnt pathway gene expression and their clinical correlation in oral squamous cell carcinoma. *Indian J. Dent. Res.* **2018**, *29*, 291–297, doi:10.4103/ijdr.IJDR_375_17.
4. Lyu, Q.; Jin, L.; Yang, X.; Zhang, F. LncRNA MINCR activates Wnt/β-catenin signals to promote cell proliferation and migration in oral squamous cell carcinoma. *Pathol. Res. Pract.* **2019**, *215*, 924–930, doi:10.1016/j.prp.2019.01.041.
5. Liu, B.; Cao, G.; Dong, Z.; Guo, T. Effect of microRNA-27b on cisplatin chemotherapy sensitivity of oral squamous cell carcinoma via FZD7 signaling pathway. *Oncol. Lett.* **2019**, *18*, 667–673, doi:10.3892/ol.2019.10347.
6. Peng, C.; Jia, X.; Xiong, Y.; Yin, J.; Li, N.; Deng, Y.; Luo, K.; Zhang, Q.; Wang, C.; Zhang, Z.; et al. The 14-3-3σ/GSK3β/β-catenin/ZEB1 regulatory loop modulates chemo-sensitivity in human tongue cancer. *Oncotarget* **2015**, *6*, 20177–20189, doi:10.18632/oncotarget.3896.
7. Zhong, W.; Xu, Z.; Wen, S.; Xie, T.; Wang, F.; Wang, Q.; Chen, J. Long non-coding RNA myocardial infarction associated transcript promotes epithelial-mesenchymal transition and is an independent risk factor for poor prognosis of tongue squamous cell carcinoma. *J. Oral Pathol. Med.* **2019**, *48*, 720–727, doi:10.1111/jop.12892.
8. Zhang, P. ping; Xu, X. ying; Gao, Z. nan Effect of hypoglycosylated E-cadherins on proliferation and invasiveness of tongue squamous cell carcinoma. *Shanghai Kou Qiang Yi Xue.* **2014**, *23*, 1–6.
9. Yan, Q.; Su, Y.; Zhou, Y.; Zhu, H.; Yang, X.; Xu, J. Interleukin-23 strengthens the anti-apoptotic and drug resistance of human tongue squamous cell carcinoma through the Wingless-related integration site/β-catenin pathway. *Hua Xi Kou Qiang Yi Xue Za Zhi.* **2015**, *33*, 249–254, doi:10.7518/hxkq.2015.03.007.
10. Fang, Z. Role and mechanism of Galectin-3 gene in proliferation, invasion, and apoptosis of oral squamous cell carcinoma. *Hua Xi Kou Qiang Yi Xue Za Zhi.* **2018**, *36*, 404–409.

12.4. Review (n = 4)

1. Clevers H. Wnt/beta-catenin signaling in development and disease. *Cell* **2006**, 469–480.
2. Shiah, S.G.; Shieh, Y.S.; Chang, J.Y. The Role of Wnt Signaling in Squamous Cell Carcinoma. *J. Dent. Res.* **2016**, *95*, 129–134, doi:10.1177/0022034515613507.
3. Varelas, X.; Bouchie, M.P.; Kukuruzinska, M.A. Protein N-glycosylation in oral cancer: Dysregulated cellular networks among DPAGT1, E-cadherin adhesion and canonical Wnt signaling. *Glycobiology* **2014**, *24*, 579–591, doi:10.1093/glycob/cwu031.
4. Xie, J.; Huang, L.; Lu, Y.G.; Zheng, D.L. Roles of the Wnt Signaling Pathway in Head and Neck Squamous Cell Carcinoma. *Front. Mol. Biosci.* **2021**, *7*, doi:10.3389/fmolb.2020.590912.

12.5. *Overlapping Population (n = 3)*

1. da Silva, S.D.; Morand, G.B.; Alobaid, F.A.; Hier, M.P.; Mlynarek, A.M.; Alaoui-Jamali, M.A.; Kowalski, L.P. Epithelial-mesenchymal transition (EMT) markers have prognostic impact in multiple primary oral squamous cell carcinoma. *Clin. Exp. Metastasis.* **2015**, *32*, 55–63, doi:10.1007/s10585-014-9690-1.
2. Hanemann, J.A.C.; Oliveira, D.T.; Nonogaki, S.; Nishimoto, I.N.; de Carli, M.L.; Landman, G.; Kowalski, L.P. Expression of E-cadherin and β-catenin in basaloid and conventional squamous cell carcinoma of the oral cavity: Are potential prognostic markers? *BMC Cancer.* **2014**, *14*, doi:10.1186/1471-2407-14-395.
3. Zhang, W.M.; Lo Muzio, L.; Rubini, C.; Yan, G. Effect of WNT-1 on β-catenin expression and its relation to Ki-67 and tumor differentiation in oral squamous cell carcinoma. *Oncol. Rep.* **2005**, *13*, 1095–1099, doi:10.3892/or.13.6.1095.

12.6. *Animal Experimentation (n = 2)*

1. Li, G.H.; Ma, Z.H.; Wang, X. Long non-coding RNA CCAT1 is a prognostic biomarker for the progression of oral squamous cell carcinoma via miR-181a-mediated Wnt/β-catenin signaling pathway. *Cell Cycle.* **2019**, *18*, 2902–2913.
2. Nie, D.; Wang, Z.; Zhang, Y.; Pang, D.; Ouyang, H.; Li, L. Fat-1 gene inhibits human oral squamous carcinoma cell proliferation through downregulation of β-catenin signaling pathways. *Exp. Ther. Med.* **2016**, *11*, 191–196, doi:10.3892/etm.2015.2847.

12.7. *Off Topic (n = 2)*

1. Kina, S.; Kawabata-Iwakawa, R.; Miyamoto, S.; Arasaki, A.; Sunakawa, H.; Kinjo, T. A molecular signature of well-differentiated oral squamous cell carcinoma reveals a resistance mechanism to metronomic chemotherapy and novel therapeutic candidates. *J. Drug Target.* **2021**, *29*, 1118–1127, doi:10.1080/1061186X.2021.1929256.
2. Gokulan, R.; Halagowder, D. Expression pattern of Notch intracellular domain (NICD) and Hes-1 in preneoplastic and neoplastic human oral squamous epithelium: Their correlation with c-Myc, clinicopathological factors and prognosis in Oral cancer. Med. Oncol. **2014**, *31*, doi:10.1007/s12032-014-0126-1.

12.8. *Gene Alterations (n = 1)*

1. Marimuthu, M.; Andiappan, M.; Wahab, A.; Muthusekhar, M.; Balakrishnan, A.; Shanmugam, S. Canonical Wnt pathway gene expression and their clinical correlation in oral squamous cell carcinoma. *Indian J. Dent. Res.* **2018**, *29*, 291–297, doi:10.4103/ijdr.IJDR_375_17.

12.9. *No Oral Cancer (n = 1)*

1. de Silva, B.S.F.; de Castro, C.A.; Von Zeidler, S.L.V.; de Sousa, S.C.O.M.; Batista, A.C.; Yamamoto-Silva, F.P. Altered β-catenin expression in oral mucosal dysplasia: A comparative study. *J. Appl. Oral Sci.* **2015**, *23*, 472–478, doi:10.1590/1678-775720150150.

13. Protocol

Review title:

- Prognostic and clinicopathological significance of the aberrant expression of β-catenin in oral squamous cell carcinoma: a systematic review and meta-analysis protocol.

Anticipated or actual start date: September 2021
Anticipated completion date: April, 2022

Table S4. Stage of review at the date of protocol preparation (Oct, 2020).

Review Stage	Started	Completed
Preliminary searches	Yes	Yes
Piloting of the study selection process	Yes	Yes
Formal screening of search results against eligibility criteria	Yes	Yes
Data extraction	No	No
Risk of bias (quality) assessment	No	No
Data analysis	No	No

Review team members and their organizational affiliations:

- Pablo Ramos-Garcia - School of Dentistry, University of Granada, Granada, Spain
- Miguel Ángel González-Moles - School of Dentistry, University of Granada, Granada, Spain

Funding sources/sponsors:

- None

Conflicts of interest:

- None

Review question:

What is the prognostic and clinicopathological significance of the aberrant expression of β-catenin (assessed through the immunohistochemical loss of membrane expression, cytoplasmic and nuclear expression) in oral squamous cell carcinoma patients?

Searches:

Studies published in PubMed, Embase, Web of Science and Scopus (upper limit = October 2021), using both database thesaurus terms (*i.e.* MeSH or Emtree terms) and free text words:

PubMed—("beta Catenin"[Mesh Terms] OR "β-catenin"[All Fields] or "beta-catenin"[All Fields] or "b-catenin"[All Fields] OR "CTNNB"[All Fields] OR "Wnt Signaling Pathway"[Mesh Terms] OR "wnt"[All Fields] OR "Armadillo Domain Proteins"[Mesh] OR "armadillo"[All Fields]) AND ("mouth"[MeSH Terms] OR "mouth"[All Fields] OR "oral"[All Fields]) AND ("carcinoma, squamous cell"[MeSH Terms] OR ("carcinoma"[All Fields] AND "squamous"[All Fields] AND "cell"[All Fields]) OR "squamous cell carcinoma"[All Fields] OR "Neoplasms"[Mesh Terms] OR neopla*[All Fields] OR "cancer"[All Fields])

Embase—('beta catenin'/exp OR 'β-catenin' OR 'beta-catenin' OR 'b-catenin' OR 'ctnnb gene'/exp OR 'CTNNB' OR 'canonical Wnt signaling'/exp OR 'wnt' OR 'armadillo domain protein'/exp OR 'armadillo') AND ('mouth'/exp OR 'mouth' OR 'oral') AND ('squamous cell carcinoma'/exp OR 'carcinoma' OR 'malignant neoplasm'/exp OR 'neoplas*' OR 'cancer')

Web of Science—TS=("β-catenin" OR "beta-catenin" OR "b-catenin" OR "CTNNB" OR "wnt" OR "armadillo") AND TS=(mouth OR oral) AND TS=("squamous cell carcinoma" OR neoplas* or cancer)

Scopus—TITLE-ABS-KEY(("β-catenin" OR "beta-catenin" OR "b-catenin" OR "CTNNB" OR "wnt" OR "armadillo") AND (mouth OR oral) AND ("squamous cell carcinoma" OR neoplas* or cancer))

An additional screening will also be performed handsearching the reference lists of retrieved included studies and using Google Scholar.

Condition or domain being studied:

Oral cancer presents a worldwide incidence of 377,713 new cases and 177,757 deaths per year (GLOBOCAN, IARC, WHO). Oral squamous cell carcinoma accounts for approximately 90% of oral malignancies and has a 5-year mortality rate close to 50%. Therefore, the future identification and validation of prognostic molecular markers is needed to identify high risk patients, and predict treatment response.

Participants/population:

Patients diagnosed with oral squamous cell carcinoma. Studies researching patients with distinct anatomical location or histopathological type will be excluded.

Intervention(s), exposure(s):

We will evaluate studies in which the aberrant expression of β-catenin (assessed through the immunohistochemical loss of membrane expression, cytoplasmic and nuclear expression) were evaluated in tumour samples from patients with oral squamous cell carcinomas. The aberrant expression of β-catenin (based on the cut-off value chosen by the authors) will be categorized and considered as the exposition group.

Comparator(s)/control:

Control/comparator group will be represented by the group of oral squamous cell carcinoma patients without β-catenin aberrant expression.

Primary outcome(s):

Prognostic variables: survival and recurrence variables.

Clinicopathological variables: T and N status, clinical stage and histological grade.

Secondary outcome(s):

Secondary clinicopathological parameters (e.g., tumour thickness, number of metastatic lymph nodes, extracapsular spread, tumour growth pattern, mode of invasion in tumour front, perineural/lymphatic/vascular/bone/skin invasion, tumour margins, etc). These datasets are rarely reported in primary-level studies. if meta-analysis of these variables cannot be performed, an albatross plot will be constructed and narrative synthesis will be performed.

Types of study to be included:

Inclusion criteria will be:

- Original research articles published in all languages, without time or study design restrictions.
- β-catenin aberrant alterations evaluated in human oral squamous cell carcinomas.
- Analysis of the outcomes of interest (please, see below) and their relationships with β-catenin assessed using immunohistochemistry..

Exclusion criteria will be:

- Retractions, case reports, editorials, letters, personal opinions or comments, meeting abstracts, books, reviews or meta-analyses.
- In vitro or animal research.
- No oral squamous cell carcinomas (i.e., from different anatomic areas or histopathology).
- Evaluation of β-catenin/CTNNB genomic alterations.
- Lack essential data for OR/HR (with 95%CI) estimations.

Data extraction (selection and coding):

Data will be gathered on the first author, publication language, publication date, country and continent, sample size, anatomical site and subsites affected, sex and age of patients, tobacco and alcohol consumption, treatment modality, recruitment and follow up period, study design, methodology and the frequency of proteins expression, immunohistochemical methods (i.e., anti-β-catenin antibody, dilution, incubation time and temperature), cut-off value, scoring system, subcellular β-catenin location and the relative frequency of cases presenting β-catenin aberrant

expression (sub-categorized as loss of cell membrane, cytoplasmic-nuclear expression, or not defined in primary-level studies).

If during data extraction process it is desirable to combine two or more different datasets expressed as means±SD from subgroups into a single group, the Cochrane Handbook [1] formula will be applied. Data expressed as order statistics (i.e., median, interquartile range and/or maximum-minimum values) will be computed and transformed, if possible, into means ± standard deviation (SD) using the methods proposed by Luo et al. (2018) [2] and Wan et al. (2014) [3].

Risk of bias (quality) assessment:

The risk of bias in individual studies will be assessed using the Quality in Prognostic Studies (QUIPS) tool, developed by Cochrane prognosis methods group. Specifically, it contains 6 domains: study participation, study attrition, prognostic factor measurement, outcome measurement, study confounding and statistical analysis and reporting. Each domain will be rated as low, moderate or high risk of bias for each study. An overall rating will be also assigned to individual studies for statistical purposes (i.e., to explore the potential influence of quality/risk of bias on pooled estimates).

Strategy for data synthesis:

Odds ratios (OR) and 95% confidence intervals (CI) will be used as the measure of association to determine the association between β-catenin aberrant expression and clinicopathological features (T status [T3/4 vs, T1/2], N status [N+ vs, N−], clinical stage [III/IV vs, I/II] and histological grade [II/III vs, I]).

Hazard ratios (HR) and 95% CI will be used as the measure of association to estimate the impact of β-catenin aberrant expression on time-to-event parameters (OS and DFS). If HR with 95%CI are not explicitly reported by the authors, they will be calculated by us using Parmar and Tierney methods. If only Kaplan-Meier curves are reported, HR data will be extracted using Engauge Digitizer 4.1 software. In both meta-analyses, if data are not reported as OR or HR, different ratio metrics will be extracted and pooled as an approximation of these measures if appropriate (rare outcomes under study (<5%) and an effect size not too high [>2] or low [<0.5]). If these measures derived both from univariable and multivariable models, data were extracted from multivariable, reflecting a greater adjustment for potentially confounding variables.

In meta-analysis, OR and HR will be pooled where appropriate (taking into account heterogeneity degree with a low number of studies, making it impossible to assess their potential sources) using random effects models, which accounts for the possibility that are different underlying results among study subpopulations (i.e., oral squamous cell carcinoma subsites, geographical differences, or based on different experimental methods). Forest plots will be constructed to examine the overall effect. Heterogeneity between studies will be checked using the χ^2 based Cochran's Q test ($p < 0.10$) and Higgins I^2 statistic. Subgroups analyses, meta-regression and sensitivity analyses will also be performed (please, see above).

Finally, funnel plots will be constructed where appropriate, to assess small-study effects such as publication bias. Egger's regression tests ($p < 0.10$) will also be used to statistically assess funnel plots asymmetry. Stata v.16.1 will be employed for all tests, computer-typing commands.

Analysis of subgroups or subsets:

Preplanned subgroup meta-analyses (by geographical area, immunohistochemical methods and overall risk of bias) and meta-regression (sex, age, clinical stage, follow up period, tobacco and alcohol) analyses will be performed, if enough observations are available, to explore the relationships between the precedent outcomes in these subgroups. If a low number of studies are included in meta-regression analyses, bootstrap methods will be implemented to improve the precision of estimations. Finally, sensitivity analyses will be performed to explore the influence of primary-level studies (aka "leave-one-out method") on the overall pooled results, to test the reliability the results.

Protocol References

1. Higgins, J.P.; Green, S. Cochrane Handbook for Systematic Reviews of Interventions: Cochrane Book Series. Available online: http://handbook.cochrane.org
2. Luo, D.; Wan, X.; Liu, J.; Tong, T. Optimally estimating the sample mean from the sample size, median, mid-range, and/or mid-quartile range. Stat. Methods Med. Res. **2018**, *27*, 1785–1805. https://doi.org/10.1177/0962280216669183.
3. Wan, X.; Wang, W.; Liu, J.; Tong, T. Estimating the sample mean and standard deviation from the sample size, median, range and/or interquartile range. BMC Med. Res. Methodol. **2014**, *14*, 135. https://doi.org/10.1186/1471-2288-14-135.

© 2021 by the authors. Licensee MDPI, Basel, Switzerland. This article is an open access article distributed under the terms and conditions of the Creative Commons Attribution (CC BY) license (http://creativecommons.org/licenses/by/4.0/).
